# Characterisation of Indian gut microbiome for B-vitamin production and its comparison with Chinese cohort

**DOI:** 10.1017/S0007114523002179

**Published:** 2024-02-28

**Authors:** Nisha Chandel, Pramod R. Somvanshi, Vivek Thakur

**Affiliations:** Department of Systems and Computational Biology, University of Hyderabad, Gachibowli, Hyderabad, 500046, India

**Keywords:** Human gut microbiome, B-vitamins biosynthesis, Cooperative interactions, Abundance and Prevalence, Indian and Chinese cohorts, Lifestyle, Diet

## Abstract

The human gut microbiota can biosynthesize essential micronutrients such as B-vitamins and is also known for its metabolic cooperative behaviour. The present study characterises such B-vitamin biosynthesizers, their biosynthetic pathways, explores their prevalence and abundance, examines how lifestyle or diet affects them in multiple Indian cohorts and compares it with the Chinese cohort. To achieve this, publicly available faecal metagenome data of healthy individuals from multiple Indian (two urban and three tribal populations) and a Chinese cohort were analysed. The distribution of prevalence and abundance of B-vitamin biosynthesizers showed similar profiles to that of the entire gut community of the Indian cohort, and there were 28 B-vitamin biosynthesizers that had modest or higher prevalence and abundance. The omnivorous diet affected only the prevalence of a few B-vitamin biosynthesizers; however, lifestyle and/or location affected both prevalence and abundance. A comparison with the Chinese cohort showed that fourteen B-vitamin biosynthesizers were significantly more prevalent and abundant in Chinese as compared with Indian samples (False Discovery Rate (FDR) <= 0·05). The metabolic potential of the entire gut community for B-vitamin production showed that within India, the tribal cohort has a higher abundance of B-vitamin biosynthesis pathways as compared with two urban cohorts namely, Bhopal and Kasargod, and comparison with the Chinese cohort revealed a higher abundance in the latter group. Potential metabolic cooperative behaviour of the Indian gut microbiome for biosynthesis of the B-vitamins showed multiple pairs of species showed theoretical complementarity for complete biosynthetic pathways genes of thiamine, riboflavin, niacin and pantothenate.

The human gut microbiome is an ecological community of microorganisms that have mutualistic or pathogenic relationships among themselves as well as with the host^(1,2)^. The beneficial role of human gut microbiome, particularly Bacteria, includes the fermentation of carbohydrates, which skip the host digestion machinery, and complex carbohydrates to SCFA, which are the energy sources of the host. Protein metabolism, polyphenol metabolism, antimicrobial protection, immunomodulation and production of micronutrients, such as vitamin K and B-vitamins, are other crucial roles of gut microbiota^(3–5)^.

The B-vitamins such as thiamine (B_1_), riboflavin (B_2_), niacin (B_3_), pantothenate (B_5_), pyridoxine (B_6_), biotin (B_7_), folate (B_9_) and cobalamin (B_12_) act as co-factor/ co-enzyme for several enzymatic reactions; thus, their deficiency leads to various diseases in humans such as beriberi, pellagra, immune diseases, etc. (see review by *Yoshii et al.*)^(6)^. These vitamins are water-soluble and cannot be stored in the body for later use (see review by *Lykstad et al.*)^(7)^. Since humans are incapable of synthesising them, these need to be included in the diet as per the daily requirement of the body (online Supplementary Table S1).

Different measures have been taken into account to alleviate B-vitamin deficiencies such as biofortification of staple crops and food fortification, and the latter has almost eradicated the severe vitamin deficiency diseases, but the subclinical deficiency, also known as hidden hunger, persists in developing countries^(8)^. According to a recent survey, the prevalence of B_12_ and B_9_ deficiency in Indian adolescents was 31 % and 37 %, respectively^(9)^. The human gut bacterial species are known to be the natural source of B-vitamins to the human host; hence, they are gaining much attention for use in food fortification programmes to improve vitamin content in a natural way. A study estimates at least one-third of the dietary reference intake could be contributed by the gut microbiome^(10)^, but to what extent they actually get absorbed in the colon is still not known. For instance, the presence of B-vitamin transporters except for B_12_ in the colon suggests the utilisation of bacteria-derived vitamins by the human host (online Supplementary Table S2). However, having no transporters for B_12_ absorption in the colon makes it unavailable to the host.

Characterisation of overall taxonomic groups in the gut, their abundance, prevalence and functional capability has already been reported^(11)^. Metagenomic analysis of microbe-mediated vitamin metabolism in the human gut microbiome showed the prevalence of B-vitamin metabolism genes across most abundant phyla such as Firmicutes, Bacteroidetes, Actinobacteria, Proteobacteria, Fusobacteria and Verrucomicrobia^(12)^. However, not all human gut microbiome species are capable of biosynthesis of B-vitamins. *Sharma et al.*^(13)^ reported that both *in vitro* and *in vivo* model studies showed an unaltered segment of auxotrophs in both B-vitamin-depleted and repleted conditions, which suggests community-wide sharing of B-vitamins^(14)^. But to what extent actual vitamin excretion or cell lysis contributes to this sharing is unknown. Another study by *Magnusdottir et al.*^(10)^ also showed several pairs of organisms whose B-vitamin biosynthesis pathways complemented the other species, thus providing a theoretical evidence of sharing. For instance, several genomes of phyla Proteobacteria and Bacteroidetes contained all B-vitamins biosynthesis pathways except B_12_, and in complementary pattern, 8 Firmicute genomes showed the presence of only B_12_ biosynthesis pathway^(10)^. However, complementarity/cooperativity of B-vitamin biosynthesis pathways at the gene level between pairs of species in the gut community has not been reported yet.

India is the second-largest populated country, spread across different geographical locations, having huge diversity in habitat, lifestyle, ethnicity and dietary habits, which are among the responsible factors for shaping gut microbial composition^(15)^. Reports of unique genes and microbes in the Indian gut microbial community correctly reflect the uniqueness of the Indian gut microbiome^(16)^. Despite these evidences, the status of B-vitamin biosynthesizing species in the Indian population is unexplored. *Das et al.*^(12)^ have recently examined the metagenomics and metatranscriptomics data from four countries, namely China, America, Denmark and Spain, with different health and diseased states for genes involved in vitamin biosynthesis pathways, transport and the corresponding species abundance. Healthy individuals from the Chinese population showed a higher abundance of B-vitamin biosynthesis genes compared with the other three western countries^(12)^. These observations suggest the population-specific trend in B-vitamin biosynthesizing species and B-vitamin biosynthesis pathway’s prevalence and abundance, which is yet to be explored in the Indian population.

Therefore, to get deeper insights into the status of B-vitamin biosynthesizing species and metabolism pathways, publicly available gut metagenome data from multiple healthy Indian cohorts were analysed and the results were compared with the healthy Chinese cohort mentioned above. The analysis addressed the following key questions: (a) assuming faecal microbiota as a proxy of the gut microbiome, how prevalent and abundant the B-vitamin biosynthesizing species and biosynthesis pathways are in Indian cohorts as compared to the Chinese; (b) if prevalence and abundance of B-vitamin biosynthesising species and/or its biosynthesis pathways are affected by diet, lifestyle and/or location; and (c) If species pairs exist in a gut community which can potentially interact to complete the biosynthesis of B-vitamins.

## Methods

### Known B-vitamin biosynthesizers from human gut

The microbial species/strains, for which either experimental or computational evidence for B-vitamin biosynthesis was available, were searched at NCBI-PubMed and Google scholar databases with following combination of keywords: human AND (‘B vitamin’ OR B-vitamin) AND (‘gut associated’ OR gut) AND (biosynthesizing OR bio-synthesizing OR ‘bio synthesizing’ OR producer OR supplier) AND bacteria. Search results were restricted to bacterial species that were from humans or associated with humans. Initially, 407 B-vitamin biosynthesizing species and/or strains were identified from the seventeen research/review articles selected from the literature search (online Supplementary Table S3), and a majority of the species were from Magnúsdóttir *et al.*^(10)^ and Sharma *et al.*^(13)^. Although strain-level information was available for some of the species, however, to ensure uniformity, this analysis was restricted at the species level, resulting in 284 species.

### Gut metagenome sequences from Indian and Chinese cohorts

**Fig. 1.**
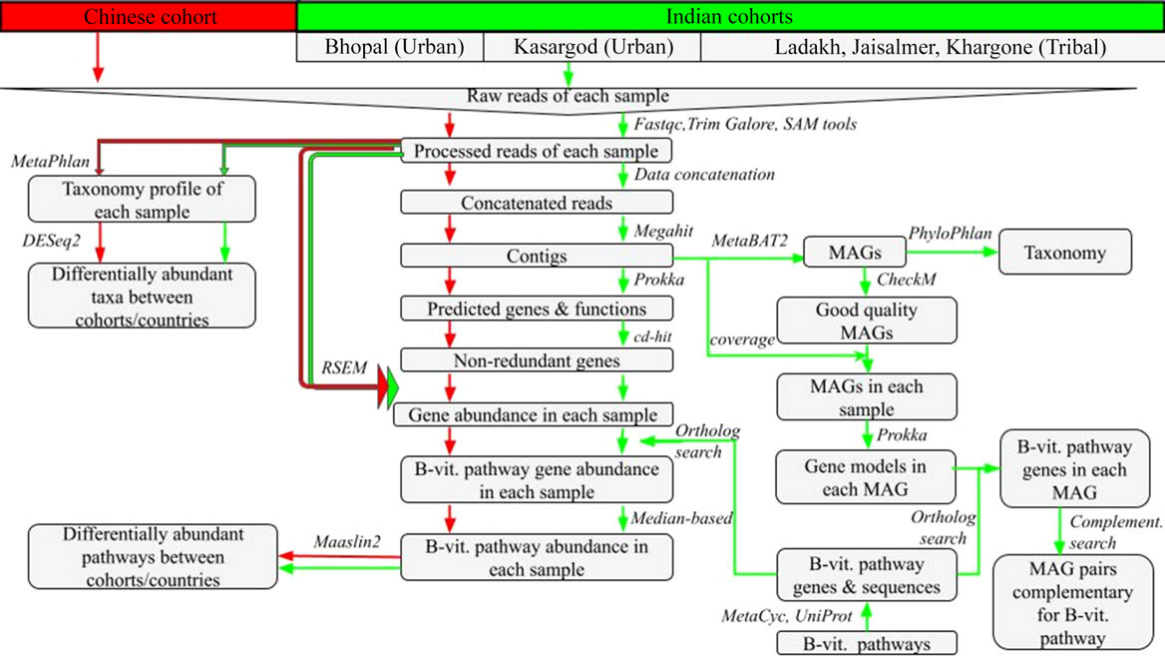
Workflow showing metagenome data analysis. Green and red colour represents steps followed in the analysis of Indian and Chinese data, respectively. The Metagenome-assembled genomes are abbreviated as MAGs.

Publicly available gut metagenomic samples from Indian and Chinese cohorts were used in this study. The Indian metagenomic data came from two studies^(15,16)^: samples from first study were from two urban populations, namely Bhopal (a city from central highlands) and Kasargod (a coastal city from south-western ghats), whereas the samples for the second study were from three tribal populations hailing from Ladakh (a cold desert from trans-Himalayan regions), Jaisalmer (a hot desert from western India) and Khargone (Satpura mountain range in central India) (Fig. 1). The samples of three tribal locations were considered as one group as a previous study from India reported no significant differences in *β* diversity measures between the tribal groups from distinct geographical regions^(17)^. Since three groups of samples/cohorts differed for biogeographical locations (and climate as well), so the effect of ‘location’ on abundance or prevalence of B-vitamin biosynthesizers or pathways was examined by comparisons among three of them. Moreover, these samples also differed in terms of mode of living and ecologies (i.e. tribal *v*. urban), so the effect of ‘lifestyle’ as a factor was inferred from the comparisons mentioned above.

The metagenomic sequences of above mentioned Indian samples were retrieved from the NCBI-SRA repository having BioProject accessions PRJNA397112^(15)^ and PRJNA531203^(16)^, with 110 and 31 samples, respectively. All samples were from healthy subjects belonging to different age groups and followed different dietary patterns (details in references^(15–16)^). In short, the dietary categories in the former study (PRJNA397112) were only vegetarian and omnivorous, whereas in the latter study (PRJNA531203), multiple dietary categories were documented, namely ovo-vegetarian, non-vegetarian, occasional poultry/red-meat/fish and vegetarian diet. To ensure consistency, we categorised them as vegetarians and omnivores (online Supplementary Table S4). The findings from Indian samples were also compared with metagenomic samples from a Chinese cohort of fifty healthy subjects, and their sequences were retrieved from the NCBI-SRA repository with BioProject accession no. PRJNA422434^(18)^. No dietary information was available for these Chinese samples. Metadata for all samples used in this study are provided in online Supplementary Table S4.

### Pre-processing of metagenome sequences

Metagenomic sequence data of all samples were Illumina paired-end reads and were checked for quality using FastQC^(19)^; reads with ambiguous bases or low-quality bases (phred score < 20) or with an adapter content were removed using Trim Galore^(20)^. Further, the host contamination was removed from the filtered reads after aligning reads to the human genome using Bowtie2^(21)^ and SAMtools^(22)^. The pre-processed reads were used for downstream analysis.

### Taxonomy profiling, prevalence and abundance analysis

MetaPhlAn 4·0 (Metagenomic Phylogenetic Analysis)^(23)^ was used for the compositional profiling of the samples using the processed sequence data, wherein the abundance of a species represents the number of individuals of that particular species. The term prevalence represents the proportion of samples in which a species is present (thus having read count >0). In order to filter out B-vitamin biosynthesizing species having very low abundance, we considered 0·1 % as the cut-off value for mean relative abundance, which was in a similar range as in a large number of studies, which considered relative abundance <1 % to exclude species from the analysis^(24)^. For prevalence, a stringent cut-off of >=50 % was used, with a rationale that if a potential B-vitamin biosynthesizing species is found in the minority population, then its importance from the perspective of public health would be diminished.

### Metabolic pathway analysis of B-vitamins

For B-vitamin biosynthesis pathway analysis, processed reads from all samples were assembled *de novo* using MEGAHIT^(25)^ to form a co-assembly (Fig. 1). Contigs with length <500 bases were removed. The contigs/scaffolds of the co-assembly were annotated using Prokka^(26)^. In order to remove redundancy among predicted gene sequences, they were clustered at 95 % identity threshold using CD-HIT tool^(27)^. To estimate gene abundance in each sample, pre-processed reads from each sample were mapped back to the non-redundant gene sequences using Bowtie2^(21)^, and read counts were obtained by RSEM tool^(28)^.

Genes involved in the B-vitamin biosynthesis pathways were obtained from MetaCyc^(29)^ with pathways IDs *THISYN-PWY* (for Thiamine), *RIBOSYN2-PWY* (Riboflavin), *PYRIDNUCSYN-PWY* (Niacin), *PANTO-PWY* (Pantothenate), *PYRIDOXSYN-PWY* (Pyridoxal phosphate), *BIOTIN-BIOSYNTHESIS-PWY* (Biotin), *FOLSYN-PWY* (Folate) and *PWY-5507* (Cobalamin), and their respective sequences were downloaded from UniProt^(30)^. Orthologs of B-vitamin biosynthesis pathway genes were identified among the genes supported by reads from the respective sample. Ortholog search was based on functional annotation and/or Reciprocal Best BLAST^(31)^. The orthologs of B-vitamin pathway genes could be identified using both approaches of orthology search, except for one gene from B_7_ pathway (UniProt id P0A6E9) and two genes from B_12_ (UniProt id Q05590 and P31570), which could be identified only through Reciprocal Best BLAST approach. The median value of normalised abundances of all genes of a pathway of a given sample was used as an estimate of pathway abundance (Fig. 1).

### Cooperative interactions for B-vitamin biosynthesis

The contigs from co-assembly were binned to obtain metagenome-assembled genomes (MAGs) using MetaBat2^(32)^ (Fig. 1). Their quality was assessed with CheckM^(33)^. MAGs with contamination >5 % and completeness <90 % were discarded. Good quality MAGs were annotated using Prokka^(26)^, and their taxonomy was predicted using PhyloPhlan^(34)^. MAGs with non-zero coverage values of contigs in each sample were considered for further analysis. All pairwise combinations of MAGs in each sample were made to form a theoretically complete biosynthesis pathway. In case of B_6_, B_7_, B_9_ and B_12_ pathways, one or two genes were either missing or not detected, so the MAG pairs were constructed with relaxed pathway completeness threshold of 85%.

### Statistical analysis

All statistical analyses were performed in R (version 4.2.2; www.r-project.org). Un-normalised species abundances between the dietary habits, or between locations, or between the countries were tested for differential abundance using DESeq2, the latter is based on a negative binomial regression model appropriate for overdispersed count data^(35)^. The effect of confounders such as age, gender, etc. was controlled in DESeq2. The multiple comparisons with (Benjamini and Hochberg procedure) adjusted *P*-values <= 0·05 were considered significant. For testing which variables (diet, lifestyle, country, etc.) can significantly affect the B-vitamin pathway abundance, a linear regression model was applied on log-transformed normalised counts using MaAsLin2^(36)^. As earlier, the confounders listed in the metadata were controlled, and the model included interaction between location and diet, as interactions among other variables were deemed less important for this analysis. Species prevalence comparisons for two and three groups were done using proportionality tests (*prop.test()* function in R) and ANOVA, respectively. Tukey’s Honestly Significant Difference (HSD) test was performed on significant results from ANOVA test. Results with *adjusted P-value* <=0·05 for multiple comparisons were considered significant. All figures were generated using the ggplot2, UpsetR and VennDiagram packages in R.

## Results

### One-tenth of (the literature reported) human gut bacteria can potentially synthesize all B-vitamins

Majority of the 284 B-vitamin biosynthesizing species identified from the literature search belong to phylum Firmicutes, Bacteroidetes, Actinobacteria, and Proteobacteria, and had a few members from Actinomycetota, Fusobacteria, Lentisphaerae and Verrucomicrobia (online Supplementary Fig. S1(a), online Supplementary Table S3). Of the 284, 18 had experimental evidence, 246 had been predicted computationally, and 20 had both experimental as well as computational evidence of B-vitamin biosynthesis (online Supplementary Fig. S1(b)). Species such as *Klebsiella pneumoniae 1 162 281* and *Salmonella enterica subsp. enterica serovar Typhimurium str. TN061786* were experimentally proven to be the biosynthesizers of all B-vitamins, and vitamin B_9_ was biosynthesized by the majority (∼ 79 %) of the experimentally proven species (online Supplementary Fig. S1(c). Approximately 9 % of the total identified species could potentially synthesise all the vitamins, while vitamins B_6_ and B_7_ were potentially synthesised by the most and the least number of species respectively (online Supplementary Fig. S1(d)). The complete list of species/strains with their metabolic potential is provided in online Supplementary Table S3.

### 28 B-vitamin biosynthesizing species had modest or higher prevalence and abundance in Indian cohorts

**Fig. 2.**
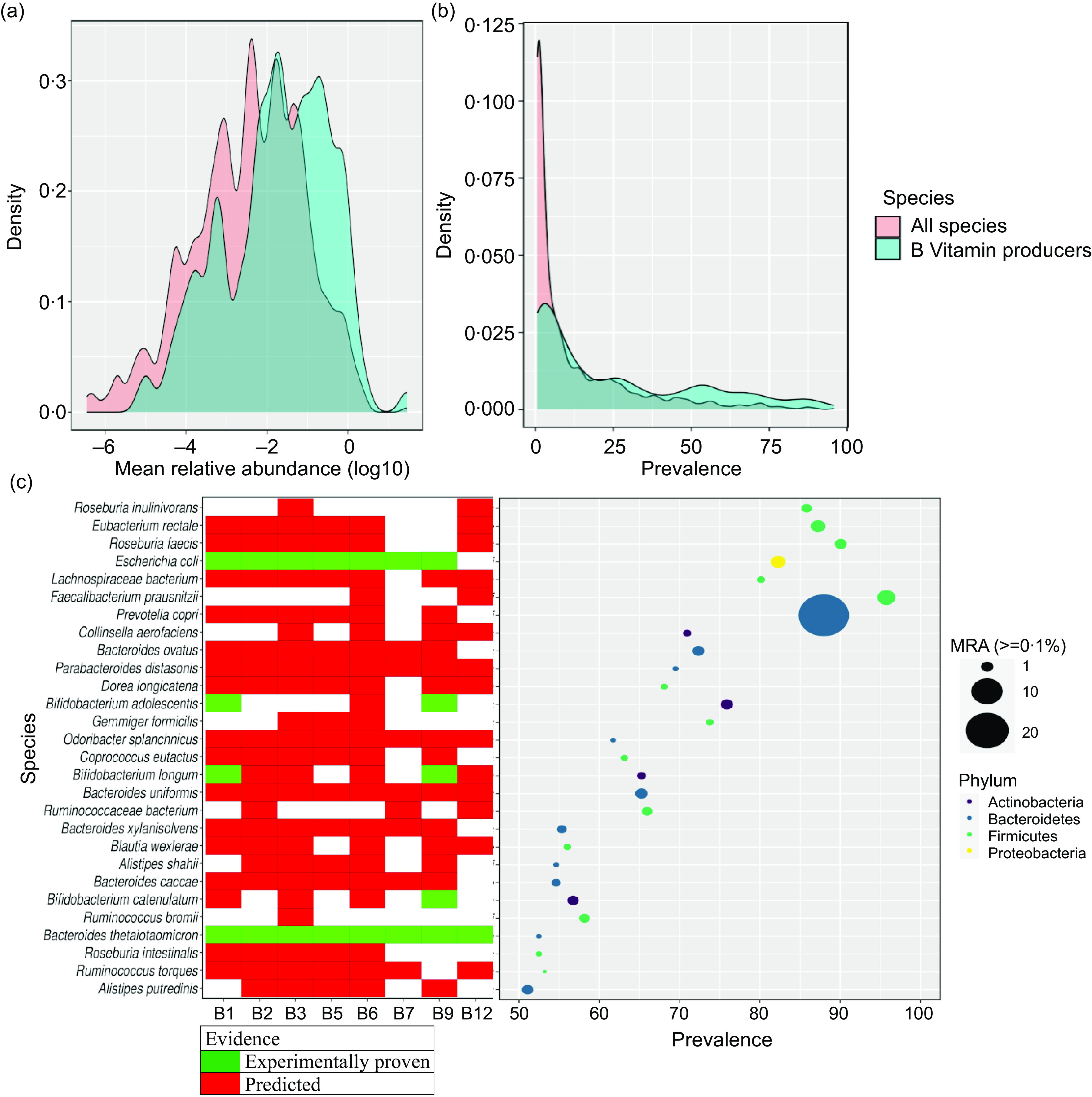
Abundance and prevalence of B-vitamin biosynthesizing species. (a) Distribution of mean relative abundance (MRA) (the x-axis is log-scaled with base 10) of the B-vitamin biosynthesizers against all gut microbes identified in the literature, (b) distribution of prevalence, (c) prevalence and abundance of selected B-vitamin biosynthesizers in Indian cohorts (see methods), and their biosynthetic profile. The white cells represent absence of a particular B-vitamin biosynthesis pathway in the corresponding species.

Compositional profiling of Indian gut metagenome samples revealed a total of 918 species, out of which 127 were (literature reported) B-vitamin biosynthesizing species (online Supplementary Table S5). Prevalence and abundance of all species and its subset of 127 B-vitamin biosynthesizers showed a skewed distribution (*P-value* <0·05), wherein the majority of the species had a prevalence and mean relative abundance less than 25 % and 0·01% respectively (Fig. 2(a–b)). Among the B-vitamin biosynthesizers, only 28 species had mean relative abundance >=0·1 % and prevalence of ≥50 %; henceforth, these thresholds are referred to as ‘modest or higher values of abundance and/or prevalence’ (Fig. 2(c)) (see Methods). The species *Prevotella copri* and *Faecalibacterium prausnitzii* had the highest abundance and prevalence, respectively. Both of these species were computationally predicted as B-vitamin biosynthesizers, where the former could synthesise all vitamins except B_7_ and B_12_, whereas the latter could synthesise only B_6_ and B_12_ (Fig. 2(c)). The enrichment of genus *Prevotella* is also well known in the Indian population^(15,16)^.

### Omnivorous diet significantly increased the prevalence of a few of the species, but had a marginal effect on the abundance

**Fig. 3.**
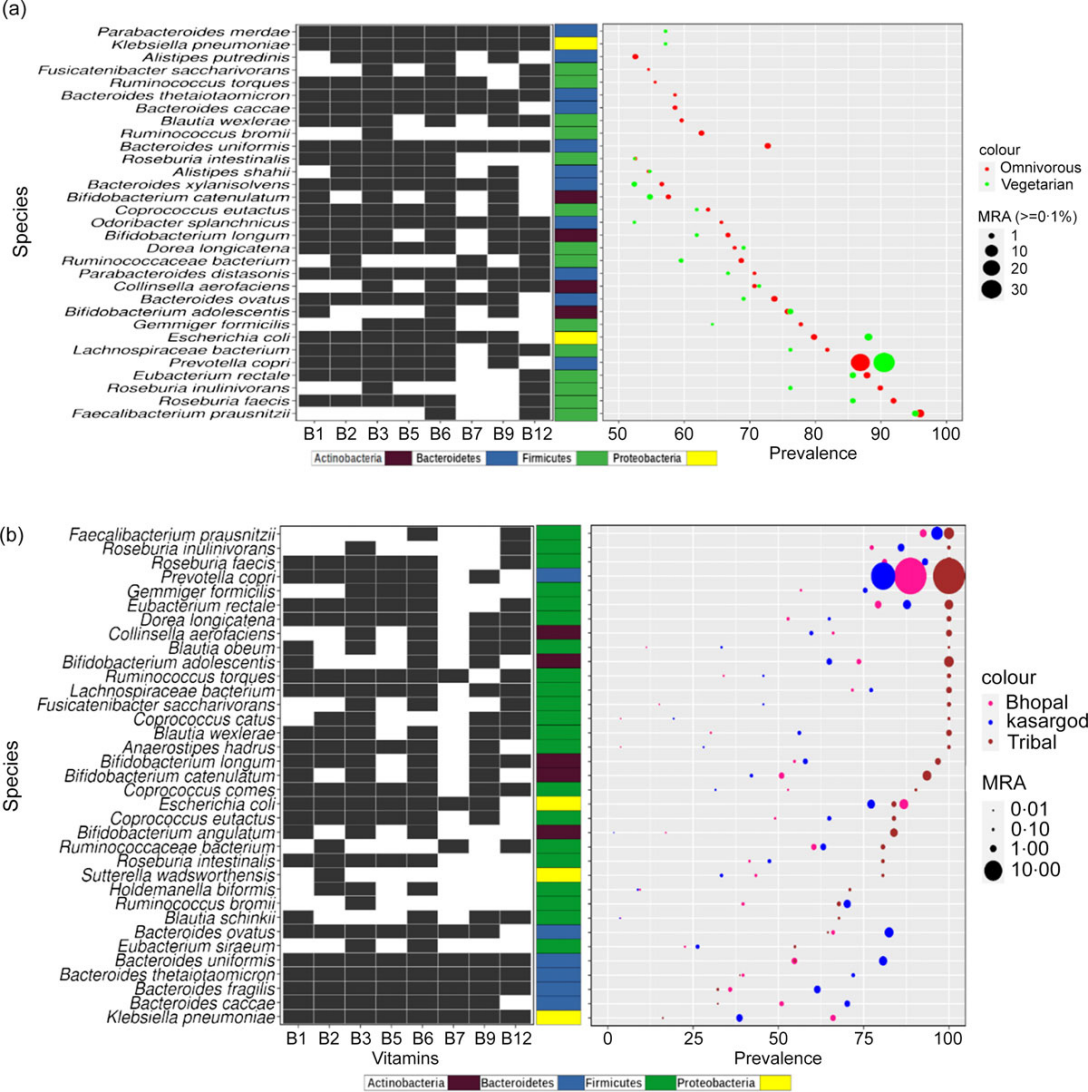
Effect of diet and location and/or lifestyle on prevalence and abundance (a) Species (*n* 31) with modest or higher prevalence and abundance in different dietary groups along with their B-vitamin biosynthesis profile, (b) species which are significantly prevalent and/or abundant species (*adj P-value<= 0·05*) in any of the three locations, and their B-vitamin biosynthetic potential. For each B-vitamin, the dark and white cells represent the presence and absence of a particular B-vitamin biosynthesis pathway in the corresponding species, respectively.

Gut microbiome composition is affected by the diet of the host^(37)^. To investigate the dietary effect on the B-vitamin biosynthesizing species, first, the species showing at least modest prevalence and abundance in each of the two dietary groups (vegetarian and omnivorous) were identified (see methods), and a total of 31 such B-vitamin biosynthesizers were obtained (Fig. 3(a)). While the prevalence of almost two-thirds (68 %) of these species was the same in both dietary groups, the remaining one-fourth (28 %) showed higher prevalence in the omnivorous group (with an increase of >10 %), whereas a similar increase in prevalence in the vegetarian group was observed only in a couple of cases (4 %) (Fig. 3(a), online Supplementary Table S6). A proportionality test for the significantly prevalent species between the two dietary groups showed a higher prevalence of *Fusicatenibacter saccharivorans*, *Bacteroides thetaiotaomicron* and *Bacteroides uniformis* in the omnivorous group (*P-value*<=0·05). However, none of the modestly prevalent and abundant species showed differential abundance between the two groups, though thirteen among the very low relative abundance (≤0·1 %) were differentially abundant, eleven being more abundant in the vegetarian group (online Supplementary Table S7, online Supplementary Fig. S2(a)).

### Tribal cohort showed a higher prevalence and abundance of several B-vitamin biosynthesizing species compared to urban cohorts

Among other factors, geographical location and lifestyle also influence the structure of the gut microbial community^(38)^. The Indian cohorts included in this study were from three different geographical locations, and they also differed in their lifestyle namely, urban *v*. tribal (see methods). First, identification of species with modest or higher prevalence and abundance in the three individual groups resulted in a total of 41 B-vitamin biosynthesizers, where 15 species were common across all three groups (online Supplementary Fig. S2(b), online Supplementary Table S8). About one-third of these species had 100 % prevalence in the tribal cohort (online Supplementary Fig. S2(c), online Supplementary Table S8), and the highest number of unique species (*n* 12) were also in the tribal cohort (Fig. 3(b), online Supplementary Fig. S2(c)).

When difference in prevalence was statistically tested by ANOVA, three-fourth of the species (*n* 31) showed significant differences *(P-value <= 0·05)*, where two-third of the species were Firmicutes, and the majority being significantly prevalent in the tribal cohort. About 80 % of differentially prevalent species did not have the potential to biosynthesize vitamin B_7_, and for those who have, the majority of them were significantly prevalent in Kasargod (urban) samples (Fig. 3(b), online Supplementary Table S9).

Comparison of the species abundance of the Tribal cohort with the other two showed a significantly higher abundance of *Blautia schinkii*, *Coprococcus cactus*, *Bifidobacterium catenulatum* and *Fusicatenibacter saccharivorans* in Tribal, and these species were also highly prevalent. On the other hand, *Klebsiella pneumoniae*, *Bacteroides caceae*, *B*. *ovatus* and *B. uniformis* were highly abundant in the urban (Bhopal and Kasargod) as compared to Tribal cohort. Except for the vitamin B_12_ in some, those abundant species had the potential to produce the remaining seven B-vitamins (Fig. 3(b), online Supplementary Fig. S3(d)). The comparison between two urban cohorts showed four species to be highly abundant in Kasargod with respect to Bhopal (namely, *Holdemanella biformis*, *Anaerostipes hardus*, *Blautia schinkii* and *Coprococcus catus*) (Fig. 3(b), online Supplementary Fig. S3, online Supplementary Table S10).

### Pathway abundances for several B-vitamins were significantly higher in the tribal as compared to urban cohorts

**Fig. 4.**
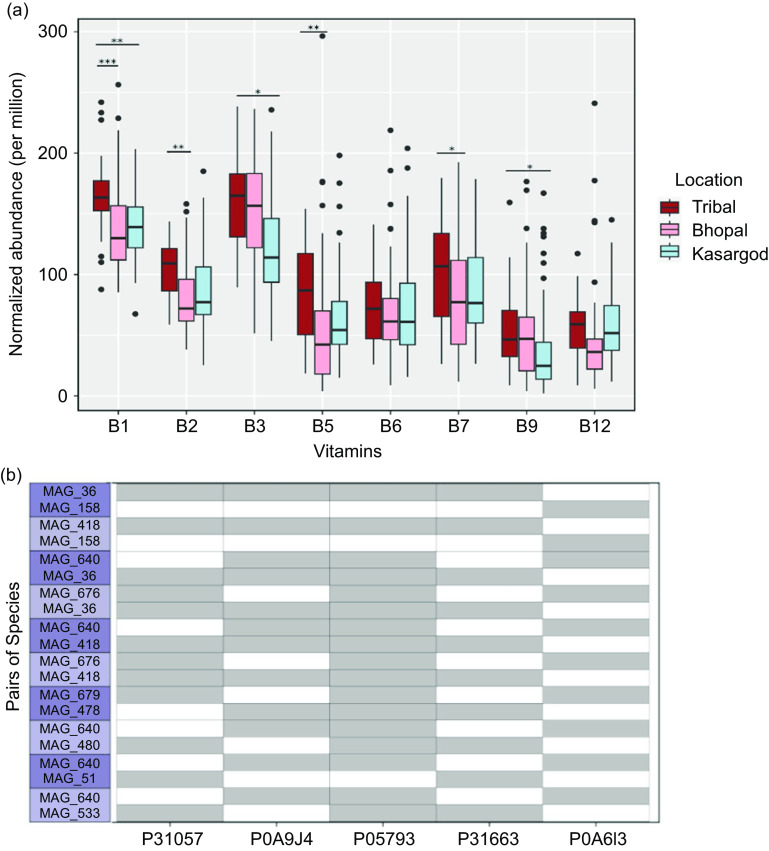
B-vitamin biosynthetic pathway abundance and cooperativity in Indian cohorts. (a) Effect of location/lifestyle on the abundance of B-vitamin biosynthetic pathways. (False Discovery Rate (FDR) = 0 *** 0·001 ** 0·01 * 0·05). (b) Ten randomly picked pairs of MAG/species that can potentially cooperate to biosynthesize pantothenate. On the X-axis, UniProt IDs of enzymes involved in pantothenate biosynthesis are shown (for details about the genes, see online Supplementary Table S12).

Beyond the known/potential B-vitamin biosynthesizing species, the pathway analysis showed that all eight B-vitamin biosynthesis pathways were present in the Indian cohorts. While B_1_ and B_3_ were the two most abundant biosynthesis pathways, the B_9_ and B_12_ were the two least abundant ones (Fig. 4(a)). Further, the abundance of the biosynthetic pathways didn’t differ with different dietary habits. On the other hand, the location/lifestyle significantly affected the abundance of at least six of them (B_1_, B_2_, B_3_, B_5_, B_7_ and B_9_). The abundances were significantly higher in tribal samples compared with either both urban locations or just one of them (online Supplementary Table S11–S12).

### Cooperative interactions for B-vitamin biosynthesis

Binning of contigs from co-assembly resulted in 730 MAGs, and only 155 survived on filtering for contamination (<=5 %) and completeness (>=90 %). Taxonomic annotation of MAGs classified 60 of them at the species level, and the remaining at the family and phylum level (online Supplementary Table S13). While almost all genes of the B-vitamin pathways were identified at the sample level (Fig. 4(a)), however, at an individual (MAG) level, the B-vitamin pathways were typically incomplete (with an exception for B_3_). Therefore, theoretical cooperative interactions between all pairs of MAGs for B-vitamin biosynthesis were searched, which showed that the MAG pairs for B_1_ and B_3_ were observed in almost all samples, whereas the pairs for B_7_ were observed in just 25 % of samples (online Supplementary Fig. S4(a)). While the samples varied for the combination of B-vitamins for which MAG pairs were available, a subset of B_1_, B_3_, B_5_ and B_6_ was the most frequently observed combination across the samples (*n* 28) (online Supplementary Fig. S4(b)). Whether these MAG pairs biologically make sense, we examined a few from many MAG pairs generated for the pantothenate biosynthetic pathway (Fig. 4(b); for other pathways see online Supplementary Fig. S5). For this vitamin, at least two transporters are known – one for uptake of its precursors (panS)^(39)^ and another for pantothenate transport (panF)^(40)^. Pantothenate precursor transporter (panS) was found in MAGs having partial or complete biosynthetic pathways but not in MAGs with the entire pathway missing. Similarly, we found the pantothenate transporter (panF) present only in those MAGs with complete biosynthetic pathway (online Supplementary Fig. S6).

### Chinese cohort showed a higher prevalence and abundance of B-vitamin biosynthesizers compared to the Indian cohorts

**Fig. 5.**
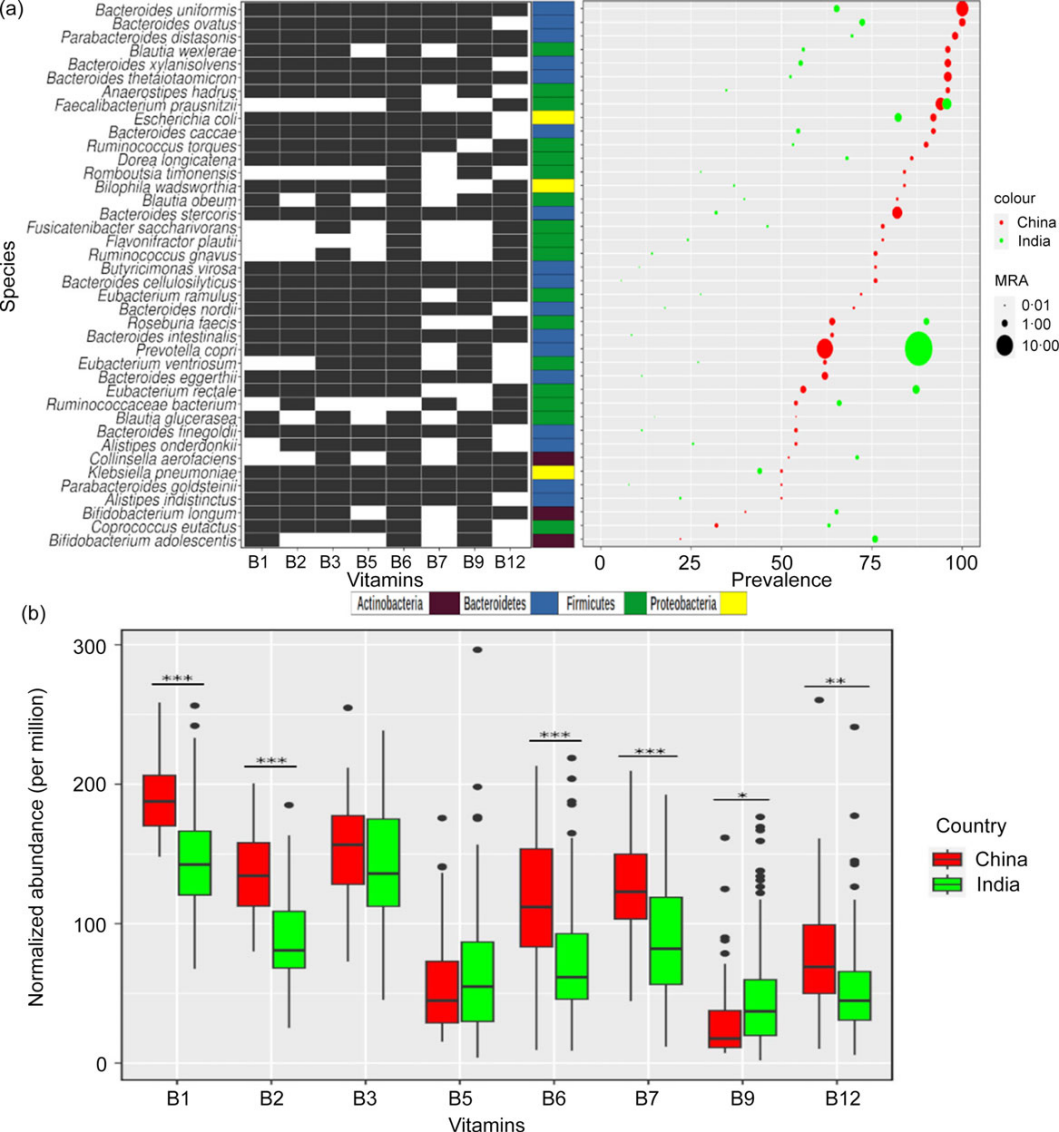
Comparison of B-vitamin biosynthesing species and pathways between Indian and Chinese cohorts. (a) B-vitamin biosynthesizing species which are differentially abundant and/or prevalent between the two cohorts (*P-adj* < = 0·05), along with their biosynthetic potential. For each B-vitamin, the dark and white cells represent the presence and absence of particular B-vitamin biosynthesis pathways in the corresponding species, respectively. (b) Differentially abundant pathways between two cohorts (P-adj = 0 *** 0·001 ** 0·01 * 0·05).

A population-level study showed the Chinese cohort having a higher abundance of B-vitamin metabolism genes as compared to a few western populations^(12)^. Therefore, the findings related to B-vitamin biosynthesizers and the biosynthetic pathways of Indian cohorts were compared with those observed in the Chinese cohort. Taxonomic profiling of the Chinese cohort showed the presence of 827 bacterial species or strains, out of which 126 were B-vitamin biosynthesizing species (online Supplementary Fig. S7(a)), and 47 species had modest or higher prevalence and abundance (see methods) as against 28 species observed in Indian samples. Out of these 47 B-vitamin biosynthesizing species, 23 were common in both cohorts with evident differences in their prevalence and abundance (online Supplementary Fig. S7(b)). The statistical testing of differences in prevalence between the two countries showed 33 significant species (Fig. 5(a), online Supplementary Table S14).

The differential abundance testing of B-vitamin biosynthesizing species between the two countries showed 25 of the species having significant differences such that, 11 species were differentially abundant, and the remaining 14 were depleted in the Indian samples compared to the Chinese (Fig. 5(a), online Supplementary Table S15. Species *Roseburia faecis*, *Prevotella copri*, *Eubacterium rectale* and *Bifidobacterium adolescentis* were both differentially abundant and prevalent in the Indian samples. Around 80 % of significantly more abundant species in Chinese samples were from phylum Bacteroidetes, all of the species can potentially biosynthesize B-vitamins with an exception of B_12_ in a few (Fig. 5(a)).

Next, the statistical testing of pathway abundance between the two countries showed that abundances of B_1_, B_2_, B_6_, B_7_ and B_12_ were significantly higher in the Chinese cohort, however, B_9_ was differentially abundant in Indian samples as compared to the Chinese (Fig. 5(b), online Supplementary Table S16). The abundance profiles of remaining pathways were similar in both populations.

## Discussion

The present study has used publicly available gut metagenome data of healthy individuals to gain an understanding of the prevalence and abundance of B-vitamin biosynthesizing species and biosynthetic pathways in the Indian cohorts. The findings from the multiple cohorts sampled across India confirmed the presence of a modest fraction of known/potential B-vitamin biosythesising species (∼45 %). Their distribution of prevalence and abundance didn’t show any significant deviation from that of the rest of the gut microbial community (Fig. 2(a–b). On the other hand, the gut microbial community of the Chinese cohort, as compared to cohorts from other countries, stood out for both B-vitamin pathways^(12)^ and B-vitamin biosynthesizers, though, with a caveat that the effect of diet as a confounder couldn’t be controlled for Chinese cohorts.

Among the B-vitamins biosynthesizing species having modest or higher abundance and prevalence in the Indian cohorts, the occurrence rate of 68 to 89 % of all B-vitamin pathways (except for B_7,_ with occurrence 35 %) was higher compared to the theoretical estimates of B-vitamin pathway occurrences in 256 human gut microbiota species, which reportedly ranged between 40 to 65 %^(10)^ (Fig. 2(c)). This could mean enhanced contribution of gut microbiomes in meeting the daily reference intake values than estimated^(10)^. However, relatively lower occurrence of B_7_ pathways can be a matter of interest for public health officials.

How the overall composition of human gut microbiota is affected by factors like diet, lifestyle and geography has been examined by multiple studies^(38,41)^; however, a similar understanding was unavailable for B-vitamin biosynthesizing gut bacteria or B-vitamin pathways. One of the most relevant works on the aforementioned problem is by *Das et al.*^(12)^, who investigated the effect of country-associated factors as well as health status on the B-vitamin pathways occurrence. The present study examines the effect of a few additional factors like diet, lifestyle and/or geographical location on B-vitamin biosynthesizers and biosynthetic pathways.

Regarding diet as a factor, the increase in the prevalence of a few species in the omnivorous group contrasted with the findings from a study on the impact of ‘plant-based’ and ‘animal-based’ diets in children of two distinct geographies, where higher microbial richness and biodiversity were observed in the ‘plant-based’ group^(41)^. Another study, however, found that the animal-based diet positively affected the abundance more often than the plant-based diet, wherein the abundance of 22 clusters of bacteria level phylotypes significantly changed while on the animal-based diet, compared to only 3 clusters for the plant-based diet^(42)^. Beyond the microbiome level, the animal-based diet was also associated with increased expression of genes for vitamin biosynthesis^(42)^. It was interesting to compare the findings of the present study with serum B_9_ and B_12_ levels of 2–3 dietary groups available from the national nutritional survey data (CNNS-2018). The B_12_ deficiency rate was found lower in non-vegetarians across all three age groups (till adolescents), however, the B_9_ deficiency levels were higher in the non-vegetarians (online Supplementary Table S17). The findings from this study as well as from the literature indicate that the vegetarian diet may be associated with overall higher species diversity, but at the micro-level involving only the B-vitamin biosynthesizers, it is the non-vegetarian diet that has a relatively greater impact on their prevalence and/or abundance.

The effect of lifestyle and/or geographical locations on moderately or highly prevalent and abundant B-vitamin biosynthesizing species showed roughly one-third of them common between urban and tribal groups and another one-third specific to the tribal group (Fig. 3(b)). Earlier studies of gut microbiomes of Indian cohorts have examined the overall differences between tribal and urban microbiomes and reported higher richness and diversity in the former and also observed a higher abundance of taxa from phylum *Bacteroides* in the urban category as against a higher abundance of taxa from phylum *Firmicutes* in the tribal category^(41)^. The above trends observed at the level of complete microbiome were very similar to that of B-vitamin biosynthesizing species, as several of the species prevalent in the tribal group also belonged to *Firmicutes*. Whether lower prevalence (and/or abundance) of B-vitamin biosynthesizing species in the urban cohorts reflects in serum B-vitamin levels, serum B_9_ and B_12_ levels from the same survey (CNNS-2018) data for urban and rural lifestyles (but no tribal data) were compared. While the B_12_ deficiency rate did not show any clear trend between urban and rural groups in the three age groups surveyed, however, B_9_ showed consistently higher deficiency rate in the urban group, in particular, the adolescent age group. A possible explanation for the higher B-vitamin biosynthesizing species in the tribal group can be substantially reduced exposure of the tribal population to antibiotics compared with urban^(43,44)^.

Beyond the profiling of predicted/validated B-vitamin biosynthsizers, profiling of complete biosynthetic pathways showed the presence of all B-vitamins in Indian cohort with an exception of B_7_ and B_12_ which missed one and two genes, respectively. It was earlier reported that biosynthetic pathways of B_2_, B_5_ and B_7_ were enriched in enterotype 1, which is driven by genus *Bacteroides*, while pathways for B_1_ and B_9_ were enriched in enterotype 2, driven by genus *Prevotella*. All of these pathways except B_7_ were found enriched in the Indian cohort, as the latter is composed of both enterotypes 1 and 2^(15,45)^.

Similar to the investigation of the effect of lifestyle on B-vitamin biosynthesizing species, their effect on B-vitamin biosynthesis pathways showed that the abundance of all, except B_6_ and B_12,_ was significantly higher in the tribal cohort as compared to the urban ones. The higher abundance of B_9_ biosynthetic pathway in the tribal cohort as compared to both urban cohorts was also in sync with the lower serum erythrocyte B_9_ levels in the urban population as compared to rural (no tribal data available in CNNS-2018)^(9)^ (online Supplementary Table S17).

A comparison of the occurrence of B-vitamin biosynthesizers and biosynthetic pathways in the Indian cohort against the Chinese cohort gave interesting trends. *Das et al.*^(12)^ had compared four global datasets including the Chinese cohort and reported that B-vitamin metabolic gene abundances were not only relatively higher in the Chinese cohort but also showed statistical association only with the Chinese samples^(12)^. This trend was also reflected at the level of B-vitamin biosynthetic pathways where, with the exception of B_9_, the other five B-vitamins namely B_1_, B_2_, B_6,_ B_7_ and B_12_ had a higher abundance in the Chinese cohort. There may be many factors behind this difference, and the role of dietary differences between the two populations^(45)^ could be the most relevant reason which can be investigated in the future. While both Chinese and Indian populations consume rice and vegetables, however, they do differ in choice/consumption of animal meat, cooking ingredients for seasoning (e.g. spices), cooking style and use of milk products.

Confirmation of whether the differential abundance of affected taxa also reflects into increased serum B-vitamin levels requires further fieldwork; particularly for the B_3_, B_6_, B_9_ and B_12_ whose daily reference intake is largely sourced from the gut microbiome. Further, bacterial species that could be negatively associated with B-vitamin deficiency should also be identified. Besides, the actual potential of the lead species could be tested in *in vitro* cultures, and species with higher intra-/extra-cellular B-vitamin concentration could be considered a probiotic candidate. For the cooperativity of B-vitamin biosynthesis, our analysis was limited to pairs, but in reality, it can be more complex, which needs to be substantiated by information on precursor transporters.

## Supplementary material

For supplementary material accompanying this paper visit https://doi.org/10.1017/S0007114523002179. click here to view supplementary material
