# Supplementary material for: Characterisation of Indian gut microbiome for B-vitamin production and its comparison with Chinese cohort
Source: Br J Nutr. 2023 Oct 2;131(4):686–97. doi: 10.1017/S0007114523002179 (PMC10803823; doi:10.1017/S0007114523002179)
Supplement: Supplementary file 1 [file S0007114523002179sup001.docx]

##

## Table S1. Recommended Dietary Allowances (RDAs) for all B-vitamins ^(47)^

| **Vitamins** | **Age** | **Birth to 6 months** | **7–12 months** | **1–3 years** | **4-8 years** | **9–13 years** | **14–18 years** | **19+ years** |
| --- | --- | --- | --- | --- | --- | --- | --- | --- |
| **Thiamin** | Male | 0.2 mg | 0.3mg | 0.5mg | 0.6mg | 0.9mg | 1.2mg | 1.2mg |
|  | Female | 0.2 mg | 0.3mg | 0.5mg | 0.6mg | 0.9mg | 1.0mg | 1.1mg |
|  | Pregnancy |  |  |  |  |  | 1.4mg | 1.4mg |
|  | Lactation |  |  |  |  |  | 1.4mg | 1.4mg |
| **Riboflavin** | Male | 0.3mg | 0.4 mg | 0.5mg | 0.6mg | 0.9mg | 1.3mg | 1.3mg |
|  | Female | 0.3mg | 0.4 mg | 0.5mg | 0.6mg | 0.9mg | 1.0mg | 1.1mg |
|  | Pregnancy |  |  |  |  |  | 1.4mg | 1.4mg |
|  | Lactation |  |  |  |  |  | 1.6mg | 1.6mg |
| **Niacin** | Male | 2mg | 4 mg | 6mg | 8mg | 12mg | 16mg |  |
|  | Female | 2mg | 4 mg | 6mg | 8mg | 12mg | 14mg |  |
|  | Pregnancy |  |  |  |  |  | 18mg |  |
|  | Lactation |  |  |  |  |  | 17mg |  |
| **Pantothenate** | Male | 1.7mg | 1.8mg | 2mg | 3mg | 4mg | 5mg |  |
|  | Female | 1.7mg | 1.8mg | 2mg | 3mg | 4mg | 5mg |  |
|  | Pregnancy |  |  |  |  |  | 6 mg |  |
|  | Lactation |  |  |  |  |  | 7 mg |  |
| **Pyridoxine** | Male | 0.1mg | 0.3 mg | 0.5mg | 0.6mg | 1.0mg | 1.3mg | 1.3mg |
|  | Female | 0.1mg | 0.3 mg | 0.5mg | 0.6mg | 1.0mg | 1.2mg | 1.3mg |
|  | Pregnancy |  |  |  |  |  | 1.9mg | 1.9mg |
|  | Lactation |  |  |  |  |  | 2.0mg | 2.0mg |
| **Biotin** | Male | 5 mcg | 6 mcg | 8 mcg | 12 mcg | 20 mcg | 25 mcg | 30 mcg |
|  | Female | 5 mcg | 6 mcg | 8 mcg | 12 mcg | 20 mcg | 25 mcg | 30 mcg |
|  | Pregnancy |  |  |  |  |  | 30 mcg | 30 mcg |
|  | Lactation |  |  |  |  |  | 35 mcg | 35 mcg |
| **Folate** | Male | 65 mcg | 80 mcg | 150 mcg | 200 mcg | 300 mcg | 400 mcg |  |
|  | Female | 65 mcg | 80 mcg | 150 mcg | 200 mcg | 300 mcg | 400 mcg |  |
|  | Pregnancy |  |  |  |  |  | 600 mcg |  |
|  | Lactation |  |  |  |  |  | 500 mcg |  |
| **Cobalamin** | Male | 0.4 mcg | 0.5 mcg | 0.9 mcg | 1.2 mcg | 1.8 mcg | 2.4 mcg |  |
|  | Female | 0.4 mcg | 0.5 mcg | 0.9 mcg | 1.2 mcg | 1.8 mcg | 2.4 mcg |  |
|  | Pregnancy |  |  |  |  |  | 2.6 mcg |  |
|  | Lactation |  |  |  |  |  | 2.8 mcg |  |

## Table S2. B-vitamin transporters in human colon

| **Vitamin** | **Transporter** | **References** |
| --- | --- | --- |
| **Thiamin** | SLC44A4 gene (hTPPT) | (48) |
|  | SLC19A2 gene | (49) |
|  | hTHTR-1 and hTHTR-2 products of the SLC19A2 and SLC19A3 genes | (50) |
|  | carrier-mediated mechanism for TPP uptake  energy-dependent, Na+- and pH-independent | (51) |
| **Riboflavin** | hRFT1  high levels of hRFT1 in the small intestine and colon suggest that hRFT1 mediates the absorption of riboflavin at these sites | (52) |
|  | RFVT3  (RFVT-1, -2, and -3, products of the SLC52A1, SLC52A2, and SLC52A3 genes, respectively) | (53) |
| **Niacin** | specific and high-affinity carrier-mediated system for uptake of luminal nicotinic acid | (54) |
| **Biotin** | Na+-dependent, carrier-mediated mechanism  Shared by Pantothenic acid | (55-58) |
| **Pantothenate** | Na+-dependent, carrier-mediated mechanism | (55) |
| **Pyridoxine** | uptake to be pH sensitive, with an increase in uptake at 6.5 and higher pH but decreased uptake at lower incubation buffer pH  temperature dependent but was Na+ independent in nature. | (56,59) |
|  |  |  |
| **Folate** | hPCFT  (duodenum > ileum > colon) | (60) |
|  | carrier-mediated, pH-dependent, DIDS-sensitive, electroneutral transport mechanism for folate uptake in the human colonic | (61) |
| **Cobalamin** | No transporter identified |  |

## Table S3. The B-vitamin biosynthesizing species which are either experimentally known or computationally predicted in the literature.

| **Vitamins** | **Species/Strain** | **Phylum** | **Evidence** | **References** |
| --- | --- | --- | --- | --- |
| **Thiamin (B1)** | *Bifidobacterium adolescentis* | Actinobacteria | Predicted and experimentally proven | (10,62)⁠ |
|  | *Bifidobacterium angulatum DSM 20098* | Actinobacteria | Predicted | (10)⁠ |
|  | *Bifidobacterium bifidum* | Actinobacteria | Predicted and experimentally proven | (10,62) |
|  | *Bifidobacterium breve* | Actinobacteria | Experimentally proven | (62)⁠ |
|  | *Bifidobacterium breve DSM 20213 = JCM 1192* | Actinobacteria | Predicted | (10) |
|  | *Bifidobacterium catenulatum DSM 16992* | Actinobacteria | Predicted | (10) |
|  | *Bifidobacterium dentium ATCC 27678* | Actinobacteria | Predicted | (10) |
|  | *Bifidobacterium longum* | Actinobacteria | Experimentally proven | (62) |
|  | *Bifidobacterium longum DJO10A* | Actinobacteria | Predicted | (10) |
|  | *Bifidobacterium longum NCC2705* | Actinobacteria | Predicted | (10) |
|  | *Bifidobacterium infantis* | Actinobacteria | Experimentally proven | (62) |
|  | *Bifidobacterium longum subsp. infantis 157F* | Actinobacteria | Predicted | (10) |
|  | *Bifidobacterium longum subsp. infantis ATCC 15697* | Actinobacteria | Predicted | (10) |
|  | *Bifidobacterium longum subsp. infantis ATCC 55813* | Actinobacteria | Predicted | (10) |
|  | *Bifidobacterium longum subsp. infantis CCUG 52486* | Actinobacteria | Predicted | (10) |
|  | *Bifidobacterium longum subsp. longum JCM 1217* | Actinobacteria | Predicted | (10) |
|  | *Bifidobacterium pseudocatenulatum DSM 20438* | Actinobacteria | Predicted | (10) |
|  | *Corynebacterium ammoniagenes DSM 20306* | Actinobacteria | Predicted | (10) |
|  | *Eggerthella sp. 1_3_56FAA* | Actinobacteria | Predicted | (10) |
|  | *Corynebacterium pseudodiphtheriticum* | Actinomycetota | Predicted | (13)⁠ |
|  | *Alistipes indistinctus YIT 12060* | Bacteroidetes | Predicted | (10) |
|  | *Bacteroides caccae ATCC 43185* | Bacteroidetes | Predicted | (10) |
|  | *Bacteroides cellulosilyticus* | Bacteroidetes | Predicted | (13) |
|  | *Bacteroides cellulosilyticus DSM 14838* | Bacteroidetes | Predicted | (10) |
|  | *Bacteroides coprocola DSM 17136* | Bacteroidetes | Predicted | (10) |
|  | *Bacteroides coprophilus DSM 18228* | Bacteroidetes | Predicted | (10) |
|  | *Bacteroides dorei DSM 17855* | Bacteroidetes | Predicted | (10) |
|  | *Bacteroides eggerthii 1_2_48FAA* | Bacteroidetes | Predicted | (10) |
|  | *Bacteroides eggerthii DSM 20697* | Bacteroidetes | Predicted | (10) |
|  | *Bacteroides finegoldii DSM 17565* | Bacteroidetes | Predicted | (10) |
|  | *Bacteroides fragilis* | Bacteroidetes | Predicted | (13) |
|  | *Bacteroides fragilis 3_1_12* | Bacteroidetes | Predicted | (10) |
|  | *Bacteroides fragilis NCTC 9343* | Bacteroidetes | Predicted and experimentally proven | (10) |
|  | *Bacteroides fragilis YCH46* | Bacteroidetes | Predicted | (10) |
|  | *Bacteroides intestinalis DSM 17393* | Bacteroidetes | Predicted | (10) |
|  | *Bacteroides ovatus ATCC 8483* | Bacteroidetes | Predicted | (10) |
|  | *Bacteroides ovatus SD CC 2a* | Bacteroidetes | Predicted | (10) |
|  | *Bacteroides ovatus SD CMC 3f* | Bacteroidetes | Predicted | (10) |
|  | *Bacteroides plebeius DSM 17135* | Bacteroidetes | Predicted | (10) |
|  | *Bacteroides sp. 1_1_30* | Bacteroidetes | Predicted | (10) |
|  | *Bacteroides sp. 1_1_6* | Bacteroidetes | Predicted | (10) |
|  | *Bacteroides sp. 2_1_16* | Bacteroidetes | Predicted | (10) |
|  | *Bacteroides sp. 2_1_22* | Bacteroidetes | Predicted | (10) |
|  | *Bacteroides sp. 2_1_33B* | Bacteroidetes | Predicted | (10) |
|  | *Bacteroides sp. 2_1_7* | Bacteroidetes | Predicted | (10) |
|  | *Bacteroides sp. 2_2_4* | Bacteroidetes | Predicted | (10) |
|  | *Bacteroides sp. 3_1_19* | Bacteroidetes | Predicted | (10) |
|  | *Bacteroides sp. 3_1_23* | Bacteroidetes | Predicted | (10) |
|  | *Bacteroides sp. 3_1_33FAA* | Bacteroidetes | Predicted | (10) |
|  | *Bacteroides sp. 3_1_40A* | Bacteroidetes | Predicted | (10) |
|  | *Bacteroides sp. 3_2_5* | Bacteroidetes | Predicted | (10) |
|  | *Bacteroides sp. 4_1_36* | Bacteroidetes | Predicted | (10) |
|  | *Bacteroides sp. 4_3_47FAA* | Bacteroidetes | Predicted | (10) |
|  | *Bacteroides sp. 9_1_42FAA* | Bacteroidetes | Predicted | (10) |
|  | *Bacteroides sp. D1* | Bacteroidetes | Predicted | (10) |
|  | *Bacteroides sp. D2* | Bacteroidetes | Predicted | (10) |
|  | *Bacteroides sp. D20* | Bacteroidetes | Predicted | (10) |
|  | *Bacteroides sp. D22* | Bacteroidetes | Predicted | (10) |
|  | *Bacteroides stercoris* | Bacteroidetes | Predicted | (13) |
|  | *Bacteroides stercoris ATCC 43183* | Bacteroidetes | Predicted | (10) |
|  | *Bacteroides thetaiotaomicron CL09T03C10* | Bacteroidetes | Predicted | (10) |
|  | *Bacteroides thetaiotaomicron dnLKV9* | Bacteroidetes | Predicted | (10) |
|  | *Bacteroides thetaiotaomicron VPI-5482* | Bacteroidetes | Predicted and experimentally proven | (10) |
|  | *Bacteroides uniformis* | Bacteroidetes | Predicted | (13) |
|  | *Bacteroides uniformis ATCC 8492* | Bacteroidetes | Predicted | (10) |
|  | *Bacteroides vulgatus* | Bacteroidetes | Predicted | (13) |
|  | *Bacteroides vulgatus ATCC 8482* | Bacteroidetes | Predicted and experimentally proven | (10) |
|  | *Bacteroides vulgatus PC510* | Bacteroidetes | Predicted | (10) |
|  | *Bacteroides xylanisolvens SD CC 1b* | Bacteroidetes | Predicted | (10) |
|  | *Bacteroides xylanisolvens XB1A* | Bacteroidetes | Predicted | (10) |
|  | *Parabacteroides goldsteinii* | Bacteroidetes | Predicted | (13) |
|  | *Parabacteroides distasonis* | Bacteroidetes | Predicted | (10,13) |
|  | *Parabacteroides johnsonii DSM 18315* | Bacteroidetes | Predicted | (10) |
|  | *Parabacteroides merdae* | Bacteroidetes | Predicted | (13) |
|  | *Parabacteroides merdae ATCC 43184* | Bacteroidetes | Predicted | (10) |
|  | *Parabacteroides sp. D13* | Bacteroidetes | Predicted | (10) |
|  | *Prevotella copri DSM 18205* | Bacteroidetes | Predicted | (6,10) |
|  | *Alistipes ihumii* | Bacteroidetes | Predicted | (13) |
|  | *Alistipes indistinctus* | Bacteroidetes | Predicted | (13) |
|  | *Bacteroides caccae* | Bacteroidetes | Predicted | (13) |
|  | *Bacteroides caecimuris* | Bacteroidetes | Predicted | (13) |
|  | *Bacteroides cellulosilyticus* | Bacteroidetes | Predicted | (13) |
|  | *Bacteroides dorei* | Bacteroidetes | Predicted | (13) |
|  | *Bacteroides faecichinchillae* | Bacteroidetes | Predicted | (13) |
|  | *Bacteroides faecis* | Bacteroidetes | Predicted | (13) |
|  | *Bacteroides finegoldii* | Bacteroidetes | Predicted | (13) |
|  | *Bacteroides fragilis* | Bacteroidetes | Predicted | (13) |
|  | *Bacteroides nordii* | Bacteroidetes | Predicted | (13) |
|  | *Bacteroides oleiciplenus* | Bacteroidetes | Predicted | (13) |
|  | *Bacteroides ovatus* | Bacteroidetes | Predicted | (13) |
|  | *Bacteroides salyersiae* | Bacteroidetes | Predicted | (13) |
|  | *Bacteroides stercorirosoris* | Bacteroidetes | Predicted | (13) |
|  | *Bacteroides stercoris* | Bacteroidetes | Predicted | (13) |
|  | *Bacteroides thetaiotaomicron* | Bacteroidetes | Predicted | (13) |
|  | *Bacteroides uniformis* | Bacteroidetes | Predicted | (13) |
|  | *Bacteroides vulgatus* | Bacteroidetes | Predicted | (13) |
|  | *Bacteroides xylanisolvens* | Bacteroidetes | Predicted | (13) |
|  | *Barnesiella intestinihominis* | Bacteroidetes | Predicted | (13) |
|  | *Butyricimonas virosa* | Bacteroidetes | Predicted | (13) |
|  | *Coprobacter fastidiosus* | Bacteroidetes | Predicted | (13) |
|  | *Muribaculum intestinale* | Bacteroidetes | Predicted | (13) |
|  | *Odoribacter splanchnicus* | Bacteroidetes | Predicted | (13) |
|  | *Parabacteroides distasonis* | Bacteroidetes | Predicted | (13) |
|  | *Parabacteroides goldsteinii* | Bacteroidetes | Predicted | (13) |
|  | *Parabacteroides gordonii* | Bacteroidetes | Predicted | (13) |
|  | *Parabacteroides merdae* | Bacteroidetes | Predicted | (13) |
|  | *Paraprevotella clara* | Bacteroidetes | Predicted | (13) |
|  | *Tidjanibacter massiliensis* | Bacteroidetes | Predicted | (13) |
|  | *Lactobacillus curvatus* | Firmicutes | Experimentally proven | (63)⁠ |
|  | *Lactobacillus plantarum* | Firmicutes | Experimentally proven | (63)⁠ |
|  | *Lactococcus lactis subsp. cremoris* | Firmicutes | Experimentally proven | (63)⁠ |
|  | *Pediococcus parvulus* | Firmicutes | Experimentally proven | (63)⁠ |
|  | *Leconostoc mesenteroides subsp. cremoris* | Firmicutes | Experimentally proven | (63)⁠ |
|  | *Acidaminococcus sp. D21* | Firmicutes | Predicted | (10) |
|  | *Anaerococcus hydrogenalis DSM 7454* | Firmicutes | Predicted | (10) |
|  | *Anaerofustis stercorihominis DSM 17244* | Firmicutes | Predicted | (10) |
|  | *Anaerostipes caccae DSM 14662* | Firmicutes | Predicted | (10) |
|  | *Anaerostipes sp. 3_2_56FAA* | Firmicutes | Predicted | (10) |
|  | *Bacillus subtilis subsp. subtilis str. 168* | Firmicutes | Predicted | (10) |
|  | *Blautia hansenii DSM 20583* | Firmicutes | Predicted | (10) |
|  | *Butyrivibrio fibrisolvens 16/4* | Firmicutes | Predicted | (10) |
|  | *Clostridiales bacterium 1_7_47FAA* | Firmicutes | Predicted | (10) |
|  | *Clostridium bartlettii DSM 16795* | Firmicutes | Predicted | (10) |
|  | *Clostridium difficile CD196* | Firmicutes | Predicted and experimentally proven | (10) |
|  | *Clostridium difficile NAP07* | Firmicutes | Predicted and experimentally proven | (10) |
|  | *Clostridium difficile NAP08* | Firmicutes | Predicted and experimentally proven | (10) |
|  | *Clostridium hiranonis DSM 13275* | Firmicutes | Predicted | (10) |
|  | *Clostridium hylemonae DSM 15053* | Firmicutes | Predicted | (10) |
|  | *Clostridium methylpentosum DSM 5476* | Firmicutes | Predicted | (10) |
|  | *Clostridium scindens ATCC 35704* | Firmicutes | Predicted | (10) |
|  | *Clostridium sp. L2-50* | Firmicutes | Predicted | (10) |
|  | *Clostridium sp. SS2/1* | Firmicutes | Predicted | (10) |
|  | *Clostridium sporogenes ATCC 15579* | Firmicutes | Predicted | (10) |
|  | *Coprococcus comes ATCC 27758* | Firmicutes | Predicted | (10) |
|  | *Coprococcus eutactus ATCC 27759* | Firmicutes | Predicted | (10) |
|  | *Dorea longicatena DSM 13814* | Firmicutes | Predicted | (10) |
|  | *Eubacterium hallii DSM 3353* | Firmicutes | Predicted | (10) |
|  | *Eubacterium rectale DSM 17629* | Firmicutes | Predicted | (10) |
|  | *Eubacterium rectale M104/1* | Firmicutes | Predicted | (10) |
|  | *Lachnospiraceae bacterium 5_1_63FAA* | Firmicutes | Predicted | (10) |
|  | *Megamonas hypermegale ART12/1* | Firmicutes | Predicted | (10) |
|  | *Mitsuokella multacida DSM 20544* | Firmicutes | Predicted | (10) |
|  | *Phascolarctobacterium sp. YIT 12067* | Firmicutes | Predicted | (10) |
|  | *Roseburia intestinalis L1-82* | Firmicutes | Predicted | (10) |
|  | *Ruminococcus lactaris ATCC 29176* | Firmicutes | Predicted | (10) |
|  | *Ruminococcus obeum A2-162* | Firmicutes | Predicted | (10) |
|  | *Ruminococcus sp. 5_1_39BFAA* | Firmicutes | Predicted | (10) |
|  | *Ruminococcus sp. SR1/5* | Firmicutes | Predicted | (10) |
|  | *Ruminococcus torques L2-14* | Firmicutes | Predicted | (10) |
|  | *Veillonella sp. 3_1_44* | Firmicutes | Predicted | (10) |
|  | *[Clostridium] amygdalinum* | Firmicutes | Predicted | (13) |
|  | *[Clostridium] citroniae* | Firmicutes | Predicted | (13) |
|  | *[Clostridium] methylpentosum* | Firmicutes | Predicted | (13) |
|  | *[Clostridium] scindens* | Firmicutes | Predicted | (13) |
|  | *[Eubacterium] eligens* | Firmicutes | Predicted | (13) |
|  | *[Eubacterium] hallii* | Firmicutes | Predicted | (13) |
|  | *[Eubacterium] rectale* | Firmicutes | Predicted | (13) |
|  | *Anaerobium acetethylicum* | Firmicutes | Predicted | (13) |
|  | *Anaerofustis stercorihominis* | Firmicutes | Predicted | (13) |
|  | *Anaerostipes hadrus* | Firmicutes | Predicted | (13) |
|  | *Anaerotignum lactatifermentans* | Firmicutes | Predicted | (13) |
|  | *Anaerotignum propionicum* | Firmicutes | Predicted | (13) |
|  | *Blautia glucerasea* | Firmicutes | Predicted | (13) |
|  | *Blautia luti* | Firmicutes | Predicted | (13) |
|  | *Blautia obeum* | Firmicutes | Predicted | (13) |
|  | *Blautia schinkii* | Firmicutes | Predicted | (13) |
|  | *Blautia wexlerae* | Firmicutes | Predicted | (13) |
|  | *Caecibacter massiliensis* | Firmicutes | Predicted | (13) |
|  | *Christensenella massiliensis* | Firmicutes | Predicted | (13) |
|  | *Clostridium perfringens* | Firmicutes | Predicted | (13) |
|  | *Coprococcus comes* | Firmicutes | Predicted | (13) |
|  | *Coprococcus eutactus* | Firmicutes | Predicted | (13) |
|  | *Dorea longicatena* | Firmicutes | Predicted | (13) |
|  | *Emergencia timonensis* | Firmicutes | Predicted | (13) |
|  | *Eubacterium callanderi* | Firmicutes | Predicted | (13) |
|  | *Eubacterium limosum* | Firmicutes | Predicted | (13) |
|  | *Eubacterium ramulus* | Firmicutes | Predicted | (13) |
|  | *Peptococcus niger* | Firmicutes | Predicted | (13) |
|  | *Pseudobutyrivibrio ruminis* | Firmicutes | Predicted | (13) |
|  | *Robinsoniella peoriensis* | Firmicutes | Predicted | (13) |
|  | *Roseburia faecis* | Firmicutes | Predicted | (13) |
|  | *Ruminococcus albus* | Firmicutes | Predicted | (13) |
|  | *Ruminococcus faecis* | Firmicutes | Predicted | (13) |
|  | *Ruminococcus gauvreauii* | Firmicutes | Predicted | (13) |
|  | *Fusobacterium gonidiaformans ATCC 25563* | Fusobacteria | Predicted | (10) |
|  | *Fusobacterium mortiferum ATCC 9817* | Fusobacteria | Predicted | (10) |
|  | *Fusobacterium sp. 1_1_41FAA* | Fusobacteria | Predicted | (10) |
|  | *Fusobacterium sp. 2_1_31* | Fusobacteria | Predicted | (10) |
|  | *Fusobacterium sp. 3_1_27* | Fusobacteria | Predicted | (10) |
|  | *Fusobacterium sp. 3_1_33* | Fusobacteria | Predicted | (10) |
|  | *Fusobacterium sp. 3_1_36A2* | Fusobacteria | Predicted | (10) |
|  | *Fusobacterium sp. 3_1_5R* | Fusobacteria | Predicted | (10) |
|  | *Fusobacterium sp. 4_1_13* | Fusobacteria | Predicted | (10) |
|  | *Fusobacterium sp. 7_1* | Fusobacteria | Predicted | (10) |
|  | *Fusobacterium sp. D11* | Fusobacteria | Predicted | (10) |
|  | *Fusobacterium sp. D12* | Fusobacteria | Predicted | (10) |
|  | *Fusobacterium ulcerans ATCC 49185* | Fusobacteria | Predicted | (10) |
|  | *Fusobacterium varium ATCC 27725* | Fusobacteria | Predicted | (10) |
|  | *Victivallis vadensis* | Lentisphaerae | Predicted | (13) |
|  | *Acinetobacter junii SH205* | Proteobacteria | Predicted | (10) |
|  | *Bilophila wadsworthia 3_1_6* | Proteobacteria | Predicted | (10) |
|  | *Campylobacter coli JV20* | Proteobacteria | Predicted | (10) |
|  | *Campylobacter upsaliensis JV21* | Proteobacteria | Predicted | (10) |
|  | *Citrobacter sp. 30_2* | Proteobacteria | Predicted | (10) |
|  | *Citrobacter youngae ATCC 29220* | Proteobacteria | Predicted | (10) |
|  | *Desulfovibrio piger ATCC 29098* | Proteobacteria | Predicted | (10) |
|  | *Desulfovibrio sp. 3_1_syn3* | Proteobacteria | Predicted | (10) |
|  | *Enterobacter cancerogenus ATCC 35316* | Proteobacteria | Predicted | (10) |
|  | *Enterobacteriaceae bacterium 9_2_54FAA* | Proteobacteria | Predicted | (10) |
|  | *Escherichia coli O157:H7 str. Sakai* | Proteobacteria | Predicted | (10) |
|  | *Escherichia coli SE11* | Proteobacteria | Predicted | (10) |
|  | *Escherichia coli str. K-12 substr. MG1655* | Proteobacteria | Predicted and experimentally proven | (10) |
|  | *Escherichia coli UTI89* | Proteobacteria | Predicted | (10) |
|  | *Escherichia sp. 1_1_43* | Proteobacteria | Predicted | (10) |
|  | *Escherichia sp. 3_2_53FAA* | Proteobacteria | Predicted | (10) |
|  | *Escherichia sp. 4_1_40B* | Proteobacteria | Predicted | (10) |
|  | *Klebsiella pneumoniae 1162281* | Proteobacteria | Predicted and experimentally proven | (10) |
|  | *Klebsiella sp. 1_1_55* | Proteobacteria | Predicted | (10) |
|  | *Oxalobacter formigenes HOxBLS* | Proteobacteria | Predicted | (10) |
|  | *Proteus penneri ATCC 35198* | Proteobacteria | Predicted | (10) |
|  | *Providencia alcalifaciens DSM 30120* | Proteobacteria | Predicted | (10) |
|  | *Providencia rettgeri DSM 1131* | Proteobacteria | Predicted | (10) |
|  | *Providencia rustigianii DSM 4541* | Proteobacteria | Predicted | (10) |
|  | *Providencia stuartii ATCC 25827* | Proteobacteria | Predicted | (10) |
|  | *Ralstonia sp. 5_7_47FAA* | Proteobacteria | Predicted | (10) |
|  | *Salmonella enterica subsp. enterica serovar Typhimurium str.* | Proteobacteria | Predicted and experimentally proven | (10) |
|  | *Succinatimonas hippei YIT 12066* | Proteobacteria | Predicted | (10) |
|  | *Desulfovibrio piger* | Proteobacteria | Predicted | (13) |
|  | *Escherichia fergusonii* | Proteobacteria | Predicted | (13) |
|  | *Akkermansia muciniphila* | Verrucomicrobiota | Predicted | (13) |
|  |  |  |  |  |
|  |  |  |  |  |
|  |  |  |  |  |
|  |  |  |  |  |
|  |  |  |  |  |
| **Riboflavin (B2)** | *Bifidobacterium longum subsp. infantis ATCC 15697* | Actinobacteria | Predicted | (10) |
|  | *Corynebacterium ammoniagenes DSM 20306* | Actinobacteria | Predicted | (10) |
|  | *Adlercreutzia equolifaciens* | Actinomycetota | Predicted | (13) |
|  | *Corynebacterium pseudodiphtheriticum* | Actinomycetota | Predicted | (13) |
|  | *Alistipes indistinctus YIT 12060* | Bacteroidetes | Predicted | (10) |
|  | *Bacteroides caccae ATCC 43185* | Bacteroidetes | Predicted | (10) |
|  | *Bacteroides cellulosilyticus* | Bacteroidetes | Predicted | (13) |
|  | *Bacteroides cellulosilyticus DSM 14838* | Bacteroidetes | Predicted | (10) |
|  | *Bacteroides coprocola DSM 17136* | Bacteroidetes | Predicted | (10) |
|  | *Bacteroides coprophilus DSM 18228* | Bacteroidetes | Predicted | (10) |
|  | *Bacteroides dorei DSM 17855* | Bacteroidetes | Predicted | (10) |
|  | *Bacteroides eggerthii 1_2_48FAA* | Bacteroidetes | Predicted | (10) |
|  | *Bacteroides eggerthii DSM 20697* | Bacteroidetes | Predicted | (10) |
|  | *Bacteroides finegoldii DSM 17565* | Bacteroidetes | Predicted | (10) |
|  | *Bacteroides fragilis* | Bacteroidetes | Predicted | (13) |
|  | *Bacteroides fragilis 3_1_12* | Bacteroidetes | Predicted | (10) |
|  | *Bacteroides fragilis NCTC 9343* | Bacteroidetes | Predicted and experimentally proven | (10) |
|  | *Bacteroides fragilis YCH46* | Bacteroidetes | Predicted | (10) |
|  | *Bacteroides intestinalis DSM 17393* | Bacteroidetes | Predicted | (10) |
|  | *Bacteroides ovatus ATCC 8483* | Bacteroidetes | Predicted | (10) |
|  | *Bacteroides ovatus SD CC 2a* | Bacteroidetes | Predicted | (10) |
|  | *Bacteroides ovatus SD CMC 3f* | Bacteroidetes | Predicted | (10) |
|  | *Bacteroides plebeius DSM 17135* | Bacteroidetes | Predicted | (10) |
|  | *Bacteroides sp. 1_1_30* | Bacteroidetes | Predicted | (10) |
|  | *Bacteroides sp. 1_1_6* | Bacteroidetes | Predicted | (10) |
|  | *Bacteroides sp. 2_1_16* | Bacteroidetes | Predicted | (10) |
|  | *Bacteroides sp. 2_1_22* | Bacteroidetes | Predicted | (10) |
|  | *Bacteroides sp. 2_1_33B* | Bacteroidetes | Predicted | (10) |
|  | *Bacteroides sp. 2_1_7* | Bacteroidetes | Predicted | (10) |
|  | *Bacteroides sp. 2_2_4* | Bacteroidetes | Predicted | (10) |
|  | *Bacteroides sp. 3_1_19* | Bacteroidetes | Predicted | (10) |
|  | *Bacteroides sp. 3_1_23* | Bacteroidetes | Predicted | (10) |
|  | *Bacteroides sp. 3_1_33FAA* | Bacteroidetes | Predicted | (10) |
|  | *Bacteroides sp. 3_1_40A* | Bacteroidetes | Predicted | (10) |
|  | *Bacteroides sp. 3_2_5* | Bacteroidetes | Predicted | (10) |
|  | *Bacteroides sp. 4_1_36* | Bacteroidetes | Predicted | (10) |
|  | *Bacteroides sp. 4_3_47FAA* | Bacteroidetes | Predicted | (10) |
|  | *Bacteroides sp. 9_1_42FAA* | Bacteroidetes | Predicted | (10) |
|  | *Bacteroides sp. D1* | Bacteroidetes | Predicted | (10) |
|  | *Bacteroides sp. D2* | Bacteroidetes | Predicted | (10) |
|  | *Bacteroides sp. D20* | Bacteroidetes | Predicted | (10) |
|  | *Bacteroides sp. D22* | Bacteroidetes | Predicted | (10) |
|  | *Bacteroides stercoris* | Bacteroidetes | Predicted | (13) |
|  | *Bacteroides stercoris ATCC 43183* | Bacteroidetes | Predicted | (10) |
|  | *Bacteroides thetaiotaomicron CL09T03C10* | Bacteroidetes | Predicted | (10) |
|  | *Bacteroides thetaiotaomicron dnLKV9* | Bacteroidetes | Predicted | (10) |
|  | *Bacteroides thetaiotaomicron VPI-5482* | Bacteroidetes | Predicted and experimentally proven | (10) |
|  | *Bacteroides uniformis* | Bacteroidetes | Predicted | (13) |
|  | *Bacteroides uniformis ATCC 8492* | Bacteroidetes | Predicted | (10) |
|  | *Bacteroides vulgatus* | Bacteroidetes | Predicted | (13) |
|  | *Bacteroides vulgatus ATCC 8482* | Bacteroidetes | Predicted and experimentally proven | (10) |
|  | *Bacteroides vulgatus PC510* | Bacteroidetes | Predicted | (10) |
|  | *Bacteroides xylanisolvens SD CC 1b* | Bacteroidetes | Predicted | (10) |
|  | *Bacteroides xylanisolvens XB1A* | Bacteroidetes | Predicted | (10) |
|  | *Parabacteroides goldsteinii* | Bacteroidetes | Predicted | (13) |
|  | *Parabacteroides distasonis* | Bacteroidetes | Predicted | (10,13) |
|  | *Parabacteroides johnsonii DSM 18315* | Bacteroidetes | Predicted | (10) |
|  | *Parabacteroides merdae* | Bacteroidetes | Predicted | (13) |
|  | *Parabacteroides merdae ATCC 43184* | Bacteroidetes | Predicted | (10) |
|  | *Parabacteroides sp. D13* | Bacteroidetes | Predicted | (10) |
|  | *Prevotella copri DSM 18205* | Bacteroidetes | Predicted | (10) |
|  | *Prevotella salivae DSM 15606* | Bacteroidetes | Predicted | (10) |
|  | *Alistipes finegoldii* | Bacteroidetes | Predicted | (13) |
|  | *Alistipes obesi* | Bacteroidetes | Predicted | (13) |
|  | *Alistipes onderdonkii* | Bacteroidetes | Predicted | (13) |
|  | *Alistipes putredinis* | Bacteroidetes | Predicted | (13) |
|  | *Alistipes shahii* | Bacteroidetes | Predicted | (13) |
|  | *Alistipes timonensis* | Bacteroidetes | Predicted | (13) |
|  | *Alistipes ihumii* | Bacteroidetes | Predicted | (13) |
|  | *Alistipes indistinctus* | Bacteroidetes | Predicted | (13) |
|  | *Bacteroides caccae* | Bacteroidetes | Predicted | (13) |
|  | *Bacteroides caecimuris* | Bacteroidetes | Predicted | (13) |
|  | *Bacteroides cellulosilyticus* | Bacteroidetes | Predicted | (13) |
|  | *Bacteroides dorei* | Bacteroidetes | Predicted | (13) |
|  | *Bacteroides faecichinchillae* | Bacteroidetes | Predicted | (13) |
|  | *Bacteroides faecis* | Bacteroidetes | Predicted | (13) |
|  | *Bacteroides finegoldii* | Bacteroidetes | Predicted | (13) |
|  | *Bacteroides fragilis* | Bacteroidetes | Predicted | (13) |
|  | *Bacteroides nordii* | Bacteroidetes | Predicted | (13) |
|  | *Bacteroides oleiciplenus* | Bacteroidetes | Predicted | (13) |
|  | *Bacteroides ovatus* | Bacteroidetes | Predicted | (13) |
|  | *Bacteroides salyersiae* | Bacteroidetes | Predicted | (13) |
|  | *Bacteroides stercorirosoris* | Bacteroidetes | Predicted | (13) |
|  | *Bacteroides stercoris* | Bacteroidetes | Predicted | (13) |
|  | *Bacteroides thetaiotaomicron* | Bacteroidetes | Predicted | (13) |
|  | *Bacteroides uniformis* | Bacteroidetes | Predicted | (13) |
|  | *Bacteroides vulgatus* | Bacteroidetes | Predicted | (13) |
|  | *Bacteroides xylanisolvens* | Bacteroidetes | Predicted | (13) |
|  | *Barnesiella intestinihominis* | Bacteroidetes | Predicted | (13) |
|  | *Butyricimonas virosa* | Bacteroidetes | Predicted | (13) |
|  | *Coprobacter fastidiosus* | Bacteroidetes | Predicted | (13) |
|  | *Muribaculum intestinale* | Bacteroidetes | Predicted | (13) |
|  | *Odoribacter splanchnicus* | Bacteroidetes | Predicted | (13) |
|  | *Parabacteroides distasonis* | Bacteroidetes | Predicted | (13) |
|  | *Parabacteroides goldsteinii* | Bacteroidetes | Predicted | (13) |
|  | *Parabacteroides gordonii* | Bacteroidetes | Predicted | (13) |
|  | *Parabacteroides merdae* | Bacteroidetes | Predicted | (13) |
|  | *Paraprevotella clara* | Bacteroidetes | Predicted | (13) |
|  | *Tidjanibacter massiliensis* | Bacteroidetes | Predicted | (13) |
|  | *Acidaminococcus sp. D21* | Firmicutes | Predicted | (10) |
|  | *Anaerococcus hydrogenalis DSM 7454* | Firmicutes | Predicted | (10) |
|  | *Anaerostipes caccae DSM 14662* | Firmicutes | Predicted | (10) |
|  | *Anaerostipes sp. 3_2_56FAA* | Firmicutes | Predicted | (10) |
|  | *Bacillus subtilis* | Firmicutes | Experimentally proven | (64)⁠ |
|  | *Bacillus subtilis subsp. subtilis str. 168* | Firmicutes | Predicted | (10) |
|  | *Blautia hansenii DSM 20583* | Firmicutes | Predicted | (10) |
|  | *Butyrivibrio crossotus DSM 2876* | Firmicutes | Predicted | (10) |
|  | *Butyrivibrio fibrisolvens 16/4* | Firmicutes | Predicted | (10) |
|  | *Clostridiales bacterium 1_7_47FAA* | Firmicutes | Predicted | (10) |
|  | *Clostridium bartlettii DSM 16795* | Firmicutes | Predicted | (10) |
|  | *Clostridium bolteae ATCC BAA-613* | Firmicutes | Predicted | (10) |
|  | *Clostridium clostridioforme 2_1_49FAA* | Firmicutes | Predicted | (10) |
|  | *Clostridium difficile CD196* | Firmicutes | Predicted and experimentally proven | (10) |
|  | *Clostridium difficile NAP07* | Firmicutes | Predicted and experimentally proven | (10) |
|  | *Clostridium difficile NAP08* | Firmicutes | Predicted and experimentally proven | (10) |
|  | *Clostridium hiranonis DSM 13275* | Firmicutes | Predicted | (10) |
|  | *Clostridium sp. L2-50* | Firmicutes | Predicted | (10) |
|  | *Clostridium sp. M62/1* | Firmicutes | Predicted | (10) |
|  | *Clostridium sp. SS2/1* | Firmicutes | Predicted | (10) |
|  | *Clostridium sporogenes ATCC 15579* | Firmicutes | Predicted | (10) |
|  | *Clostridium symbiosum WAL-14163* | Firmicutes | Predicted | (10) |
|  | *Clostridium symbiosum WAL-14673* | Firmicutes | Predicted | (10) |
|  | *Coprobacillus sp. 29_1* | Firmicutes | Predicted | (10) |
|  | *Coprococcus comes ATCC 27758* | Firmicutes | Predicted | (10) |
|  | *Coprococcus eutactus ATCC 27759* | Firmicutes | Predicted | (10) |
|  | *Dorea longicatena DSM 13814* | Firmicutes | Predicted | (10) |
|  | *Erysipelotrichaceae bacterium 3_1_53* | Firmicutes | Predicted | (10) |
|  | *Eubacterium biforme DSM 3989* | Firmicutes | Predicted | (10) |
|  | *Eubacterium hallii DSM 3353* | Firmicutes | Predicted | (10) |
|  | *Eubacterium rectale DSM 17629* | Firmicutes | Predicted | (10) |
|  | *Eubacterium rectale M104/1* | Firmicutes | Predicted | (10) |
|  | *Lachnospiraceae bacterium 5_1_63FAA* | Firmicutes | Predicted | (10) |
|  | *Lachnospiraceae bacterium 8_1_57FAA* | Firmicutes | Predicted | (10) |
|  | *Lactobacillus amylolyticus DSM 11664* | Firmicutes | Predicted | (10) |
|  | *Lactobacillus antri DSM 16041* | Firmicutes | Predicted | (10) |
|  | *Lactobacillus brevis ATCC 367* | Firmicutes | Predicted | (10) |
|  | *Lactobacillus crispatus 125-2-CHN* | Firmicutes | Predicted | (10) |
|  | *Lactobacillus fermentum* | Firmicutes | Predicted and experimental evidence | (6,10,65)⁠ |
|  | *Lactobacillus fermentum IFO 3956* | Firmicutes | Predicted | (10) |
|  | *Lactobacillus plantarum CRL 2130* | Firmicutes | Experimentally proven | (6,66)⁠ |
|  | *Lactobacillus plantarum 16* | Firmicutes | Predicted | (10) |
|  | *Lactobacillus plantarum subsp. plantarum ATCC 14917* | Firmicutes | Predicted | (10) |
|  | *Lactobacillus reuteri DSM 20016* | Firmicutes | Predicted | (10) |
|  | *Lactobacillus reuteri JCM 1112* | Firmicutes | Predicted | (10) |
|  | *Lactobacillus reuteri MM2-3* | Firmicutes | Predicted | (10) |
|  | *Lactobacillus reuteri MM4-1A* | Firmicutes | Predicted | (10) |
|  | *Lactobacillus ultunensis DSM 16047* | Firmicutes | Predicted | (10) |
|  | *Listeria grayi DSM 20601* | Firmicutes | Predicted | (10) |
|  | *Megamonas hypermegale ART12/1* | Firmicutes | Predicted | (10) |
|  | *Mitsuokella multacida DSM 20544* | Firmicutes | Predicted | (10) |
|  | *Phascolarctobacterium sp. YIT 12067* | Firmicutes | Predicted | (10) |
|  | *Roseburia intestinalis L1-82* | Firmicutes | Predicted | (10) |
|  | *Ruminococcaceae bacterium D16* | Firmicutes | Predicted | (10) |
|  | *Ruminococcus obeum A2-162* | Firmicutes | Predicted | (10) |
|  | *Ruminococcus sp. 5_1_39BFAA* | Firmicutes | Predicted | (10) |
|  | *Ruminococcus sp. SR1/5* | Firmicutes | Predicted | (10) |
|  | *Ruminococcus torques ATCC 27756* | Firmicutes | Predicted | (10) |
|  | *Streptococcus equinus ATCC 9812* | Firmicutes | Predicted | (10) |
|  | *Streptococcus infantarius subsp. infantarius ATCC BAA-102* | Firmicutes | Predicted | (10) |
|  | *Veillonella sp. 3_1_44* | Firmicutes | Predicted | (10) |
|  | *Veillonella sp. 6_1_27* | Firmicutes | Predicted | (10) |
|  | *[Clostridium] celerecrescens* | Firmicutes | Predicted | (13) |
|  | *[Clostridium] clostridioforme* | Firmicutes | Predicted | (13) |
|  | *[Clostridium] symbiosum* | Firmicutes | Predicted | (13) |
|  | *[Clostridium] viride* | Firmicutes | Predicted | (13) |
|  | *Butyricicoccus pullicaecorum* | Firmicutes | Predicted | (13) |
|  | *Clostridium saudiense* | Firmicutes | Predicted | (13) |
|  | *Coprobacillus cateniformis* | Firmicutes | Predicted | (13) |
|  | *Coprococcus catus* | Firmicutes | Predicted | (13) |
|  | *Enterococcus hirae* | Firmicutes | Predicted | (13) |
|  | *Faecalicatena contorta* | Firmicutes | Predicted | (13) |
|  | *Holdemanella biformis* | Firmicutes | Predicted | (13) |
|  | *Intestinibacillus massiliensis* | Firmicutes | Predicted | (13) |
|  | *Oscillibacter ruminantium* | Firmicutes | Predicted | (13) |
|  | *Oscillibacter valericigenes* | Firmicutes | Predicted | (13) |
|  | *Peptoniphilus grossensis* | Firmicutes | Predicted | (13) |
|  | *Sporobacter termitidis* | Firmicutes | Predicted | (13) |
|  | *[Clostridium] amygdalinum* | Firmicutes | Predicted | (13) |
|  | *[Clostridium] citroniae* | Firmicutes | Predicted | (13) |
|  | *[Eubacterium] eligens* | Firmicutes | Predicted | (13) |
|  | *[Eubacterium] hallii* | Firmicutes | Predicted | (13) |
|  | *[Eubacterium] rectale* | Firmicutes | Predicted | (13) |
|  | *Anaerobium acetethylicum* | Firmicutes | Predicted | (13) |
|  | *Anaerostipes hadrus* | Firmicutes | Predicted | (13) |
|  | *Anaerotignum lactatifermentans* | Firmicutes | Predicted | (13) |
|  | *Anaerotignum propionicum* | Firmicutes | Predicted | (13) |
|  | *Blautia luti* | Firmicutes | Predicted | (13) |
|  | *Blautia wexlerae* | Firmicutes | Predicted | (13) |
|  | *Caecibacter massiliensis* | Firmicutes | Predicted | (13) |
|  | *Clostridium perfringens* | Firmicutes | Predicted | (13) |
|  | *Coprococcus comes* | Firmicutes | Predicted | (13) |
|  | *Coprococcus eutactus* | Firmicutes | Predicted | (13) |
|  | *Dorea longicatena* | Firmicutes | Predicted | (13) |
|  | *Emergencia timonensis* | Firmicutes | Predicted | (13) |
|  | *Eubacterium callanderi* | Firmicutes | Predicted | (13) |
|  | *Eubacterium limosum* | Firmicutes | Predicted | (13) |
|  | *Eubacterium ramulus* | Firmicutes | Predicted | (13) |
|  | *Peptococcus niger* | Firmicutes | Predicted | (13) |
|  | *Robinsoniella peoriensis* | Firmicutes | Predicted | (13) |
|  | *Roseburia faecis* | Firmicutes | Predicted | (13) |
|  | *Ruminococcus albus* | Firmicutes | Predicted | (13) |
|  | *Ruminococcus gauvreauii* | Firmicutes | Predicted | (13) |
|  | *Fusobacterium gonidiaformans ATCC 25563* | Fusobacteria | Predicted | (10) |
|  | *Fusobacterium mortiferum ATCC 9817* | Fusobacteria | Predicted | (10) |
|  | *Fusobacterium sp. 1_1_41FAA* | Fusobacteria | Predicted | (10) |
|  | *Fusobacterium sp. 2_1_31* | Fusobacteria | Predicted | (10) |
|  | *Fusobacterium sp. 3_1_27* | Fusobacteria | Predicted | (10) |
|  | *Fusobacterium sp. 3_1_33* | Fusobacteria | Predicted | (10) |
|  | *Fusobacterium sp. 3_1_36A2* | Fusobacteria | Predicted | (10) |
|  | *Fusobacterium sp. 3_1_5R* | Fusobacteria | Predicted | (10) |
|  | *Fusobacterium sp. 4_1_13* | Fusobacteria | Predicted | (10) |
|  | *Fusobacterium sp. 7_1* | Fusobacteria | Predicted | (10) |
|  | *Fusobacterium sp. D11* | Fusobacteria | Predicted | (10) |
|  | *Fusobacterium sp. D12* | Fusobacteria | Predicted | (10) |
|  | *Fusobacterium ulcerans ATCC 49185* | Fusobacteria | Predicted | (10) |
|  | *Fusobacterium varium ATCC 27725* | Fusobacteria | Predicted | (10) |
|  | *Victivallis vadensis* | Lentisphaerae | Predicted | (13) |
|  | *Acinetobacter junii SH205* | Proteobacteria | Predicted | (10) |
|  | *Bilophila wadsworthia 3_1_6* | Proteobacteria | Predicted | (10) |
|  | *Campylobacter coli JV20* | Proteobacteria | Predicted | (10) |
|  | *Campylobacter upsaliensis JV21* | Proteobacteria | Predicted | (10) |
|  | *Citrobacter sp. 30_2* | Proteobacteria | Predicted | (10) |
|  | *Citrobacter youngae ATCC 29220* | Proteobacteria | Predicted | (10) |
|  | *Desulfovibrio piger ATCC 29098* | Proteobacteria | Predicted | (10) |
|  | *Desulfovibrio sp. 3_1_syn3* | Proteobacteria | Predicted | (10) |
|  | *Edwardsiella tarda ATCC 23685* | Proteobacteria | Predicted | (10) |
|  | *Enterobacter cancerogenus ATCC 35316* | Proteobacteria | Predicted | (10) |
|  | *Enterobacteriaceae bacterium 9_2_54FAA* | Proteobacteria | Predicted | (10) |
|  | *Escherichia coli O157:H7 str. Sakai* | Proteobacteria | Predicted | (10) |
|  | *Escherichia coli SE11* | Proteobacteria | Predicted | (10) |
|  | *Escherichia coli str. K-12 substr. MG1655* | Proteobacteria | Predicted and experimentally proven | (10) |
|  | *Escherichia coli UTI89* | Proteobacteria | Predicted | (10) |
|  | *Escherichia sp. 3_2_53FAA* | Proteobacteria | Predicted | (10) |
|  | *Escherichia sp. 4_1_40B* | Proteobacteria | Predicted | (10) |
|  | *Helicobacter bilis ATCC 43879* | Proteobacteria | Predicted | (10) |
|  | *Helicobacter canadensis MIT 98-5491* | Proteobacteria | Predicted | (10) |
|  | *Helicobacter cinaedi CCUG 18818* | Proteobacteria | Predicted | (10) |
|  | *Helicobacter pullorum MIT 98-5489* | Proteobacteria | Predicted | (10) |
|  | *Helicobacter pylori 26695* | Proteobacteria | Predicted and experimentally proven | (10) |
|  | *Helicobacter winghamensis ATCC BAA-430* | Proteobacteria | Predicted | (10) |
|  | *Klebsiella pneumoniae 1162281* | Proteobacteria | Predicted and experimentally proven | (10) |
|  | *Klebsiella sp. 1_1_55* | Proteobacteria | Predicted | (10) |
|  | *Oxalobacter formigenes HOxBLS* | Proteobacteria | Predicted | (10) |
|  | *Oxalobacter formigenes OXCC13* | Proteobacteria | Predicted | (10) |
|  | *Proteus penneri ATCC 35198* | Proteobacteria | Predicted | (10) |
|  | *Providencia alcalifaciens DSM 30120* | Proteobacteria | Predicted | (10) |
|  | *Providencia rettgeri DSM 1131* | Proteobacteria | Predicted | (10) |
|  | *Providencia rustigianii DSM 4541* | Proteobacteria | Predicted | (10) |
|  | *Providencia stuartii ATCC 25827* | Proteobacteria | Predicted | (10) |
|  | *Ralstonia sp. 5_7_47FAA* | Proteobacteria | Predicted | (10) |
|  | *Salmonella enterica subsp. enterica serovar Typhimurium str.* | Proteobacteria | Predicted and experimentally proven | (10) |
|  | *Succinatimonas hippei YIT 12066* | Proteobacteria | Predicted | (10) |
|  | *Sutterella wadsworthensis 3_1_45B* | Proteobacteria | Predicted | (10) |
|  | *Pararhodospirillum photometricum* | Proteobacteria | Predicted | (13) |
|  | *Parasutterella excrementihominis* | Proteobacteria | Predicted | (13) |
|  | *Rhodospirillum rubrum* | Proteobacteria | Predicted | (13) |
|  | *Turicimonas muris* | Proteobacteria | Predicted | (13) |
|  | *Desulfovibrio piger* | Proteobacteria | Predicted | (13) |
|  | *Escherichia fergusonii* | Proteobacteria | Predicted | (13) |
|  | *Akkermansia muciniphila* | Verrucomicrobiota | Predicted | (13) |
|  |  |  |  |  |
|  |  |  |  |  |
|  |  |  |  |  |
|  |  |  |  |  |
|  |  |  |  |  |
|  |  |  |  |  |
| **Niacin (B3)** | *Bifidobacterium angulatum DSM 20098* | Actinobacteria | Predicted | (10) |
|  | *Bifidobacterium bifidum NCIMB 41171* | Actinobacteria | Predicted | (10) |
|  | *Bifidobacterium breve DSM 20213 = JCM 1192* | Actinobacteria | Predicted | (10) |
|  | *Bifidobacterium catenulatum DSM 16992* | Actinobacteria | Predicted | (10) |
|  | *Bifidobacterium longum DJO10A* | Actinobacteria | Predicted | (10) |
|  | *Bifidobacterium longum NCC2705* | Actinobacteria | Predicted | (10) |
|  | *Bifidobacterium longum subsp. infantis 157F* | Actinobacteria | Predicted | (10) |
|  | *Bifidobacterium longum subsp. infantis ATCC 15697* | Actinobacteria | Predicted | (10) |
|  | *Bifidobacterium longum subsp. infantis ATCC 55813* | Actinobacteria | Predicted | (10) |
|  | *Bifidobacterium longum subsp. infantis CCUG 52486* | Actinobacteria | Predicted | (10) |
|  | *Bifidobacterium longum subsp. longum JCM 1217* | Actinobacteria | Predicted | (10) |
|  | *Collinsella aerofaciens ATCC 25986* | Actinobacteria | Predicted | (10) |
|  | *Collinsella intestinalis DSM 13280* | Actinobacteria | Predicted | (10) |
|  | *Collinsella stercoris DSM 13279* | Actinobacteria | Predicted | (10) |
|  | *Corynebacterium ammoniagenes DSM 20306* | Actinobacteria | Predicted | (10) |
|  | *Collinsella aerofaciens* | Actinobacteria | Predicted | (13) |
|  | *Corynebacterium pseudodiphtheriticum* | Actinomycetota | Predicted | (13) |
|  | *Alistipes indistinctus YIT 12060* | Bacteroidetes | Predicted | (10) |
|  | *Bacteroides caccae ATCC 43185* | Bacteroidetes | Predicted | (10) |
|  | *Bacteroides cellulosilyticus* | Bacteroidetes | Predicted | (13) |
|  | *Bacteroides cellulosilyticus DSM 14838* | Bacteroidetes | Predicted | (10) |
|  | *Bacteroides coprocola DSM 17136* | Bacteroidetes | Predicted | (10) |
|  | *Bacteroides coprophilus DSM 18228* | Bacteroidetes | Predicted | (10) |
|  | *Bacteroides dorei DSM 17855* | Bacteroidetes | Predicted | (10) |
|  | *Bacteroides eggerthii 1_2_48FAA* | Bacteroidetes | Predicted | (10) |
|  | *Bacteroides eggerthii DSM 20697* | Bacteroidetes | Predicted | (10) |
|  | *Bacteroides fragilis* | Bacteroidetes | Predicted | (13) |
|  | *Bacteroides finegoldii DSM 17565* | Bacteroidetes | Predicted | (10) |
|  | *Bacteroides fragilis 3_1_12* | Bacteroidetes | Predicted | (10) |
|  | *Bacteroides fragilis NCTC 9343* | Bacteroidetes | Predicted and experimentally proven | (10) |
|  | *Bacteroides fragilis YCH46* | Bacteroidetes | Predicted | (10) |
|  | *Bacteroides intestinalis DSM 17393* | Bacteroidetes | Predicted | (10) |
|  | *Bacteroides ovatus ATCC 8483* | Bacteroidetes | Predicted | (10) |
|  | *Bacteroides ovatus SD CC 2a* | Bacteroidetes | Predicted | (10) |
|  | *Bacteroides ovatus SD CMC 3f* | Bacteroidetes | Predicted | (10) |
|  | *Bacteroides plebeius DSM 17135* | Bacteroidetes | Predicted | (10) |
|  | *Bacteroides sp. 1_1_30* | Bacteroidetes | Predicted | (10) |
|  | *Bacteroides sp. 1_1_6* | Bacteroidetes | Predicted | (10) |
|  | *Bacteroides sp. 2_1_16* | Bacteroidetes | Predicted | (10) |
|  | *Bacteroides sp. 2_1_22* | Bacteroidetes | Predicted | (10) |
|  | *Bacteroides sp. 2_1_33B* | Bacteroidetes | Predicted | (10) |
|  | *Bacteroides sp. 2_1_7* | Bacteroidetes | Predicted | (10) |
|  | *Bacteroides sp. 2_2_4* | Bacteroidetes | Predicted | (10) |
|  | *Bacteroides sp. 3_1_19* | Bacteroidetes | Predicted | (10) |
|  | *Bacteroides sp. 3_1_23* | Bacteroidetes | Predicted | (10) |
|  | *Bacteroides sp. 3_1_33FAA* | Bacteroidetes | Predicted | (10) |
|  | *Bacteroides sp. 3_1_40A* | Bacteroidetes | Predicted | (10) |
|  | *Bacteroides sp. 3_2_5* | Bacteroidetes | Predicted | (10) |
|  | *Bacteroides sp. 4_1_36* | Bacteroidetes | Predicted | (10) |
|  | *Bacteroides sp. 4_3_47FAA* | Bacteroidetes | Predicted | (10) |
|  | *Bacteroides sp. 9_1_42FAA* | Bacteroidetes | Predicted | (10) |
|  | *Bacteroides sp. D1* | Bacteroidetes | Predicted | (10) |
|  | *Bacteroides sp. D2* | Bacteroidetes | Predicted | (10) |
|  | *Bacteroides sp. D20* | Bacteroidetes | Predicted | (10) |
|  | *Bacteroides sp. D22* | Bacteroidetes | Predicted | (10) |
|  | *Bacteroides stercoris* | Bacteroidetes | Predicted | (13) |
|  | *Bacteroides stercoris ATCC 43183* | Bacteroidetes | Predicted | (10) |
|  | *Bacteroides thetaiotaomicron CL09T03C10* | Bacteroidetes | Predicted | (10) |
|  | *Bacteroides thetaiotaomicron dnLKV9* | Bacteroidetes | Predicted | (10) |
|  | *Bacteroides thetaiotaomicron VPI-5482* | Bacteroidetes | Predicted and experimentally proven | (10) |
|  | *Bacteroides uniformis* | Bacteroidetes | Predicted | (13) |
|  | *Bacteroides uniformis ATCC 8492* | Bacteroidetes | Predicted | (10) |
|  | *Bacteroides vulgatus* | Bacteroidetes | Predicted | (13) |
|  | *Bacteroides vulgatus ATCC 8482* | Bacteroidetes | Predicted and experimentally proven | (10) |
|  | *Bacteroides vulgatus PC510* | Bacteroidetes | Predicted | (10) |
|  | *Bacteroides xylanisolvens SD CC 1b* | Bacteroidetes | Predicted | (10) |
|  | *Bacteroides xylanisolvens XB1A* | Bacteroidetes | Predicted | (10) |
|  | *Parabacteroides goldsteinii* | Bacteroidetes | Predicted | (13) |
|  | *Parabacteroides distasonis* | Bacteroidetes | Predicted | (10,13) |
|  | *Parabacteroides johnsonii DSM 18315* | Bacteroidetes | Predicted | (10) |
|  | *Parabacteroides merdae* | Bacteroidetes | Predicted | (13) |
|  | *Parabacteroides merdae ATCC 43184* | Bacteroidetes | Predicted | (10) |
|  | *Parabacteroides sp. D13* | Bacteroidetes | Predicted | (10) |
|  | *Prevotella copri DSM 18205* | Bacteroidetes | Predicted | (10) |
|  | *Alistipes finegoldii* | Bacteroidetes | Predicted | (13) |
|  | *Alistipes obesi* | Bacteroidetes | Predicted | (13) |
|  | *Alistipes onderdonkii* | Bacteroidetes | Predicted | (13) |
|  | *Alistipes putredinis* | Bacteroidetes | Predicted | (13) |
|  | *Alistipes shahii* | Bacteroidetes | Predicted | (13) |
|  | *Alistipes timonensis* | Bacteroidetes | Predicted | (13) |
|  | *Alistipes ihumii* | Bacteroidetes | Predicted | (13) |
|  | *Alistipes indistinctus* | Bacteroidetes | Predicted | (13) |
|  | *Bacteroides caccae* | Bacteroidetes | Predicted | (13) |
|  | *Bacteroides caecimuris* | Bacteroidetes | Predicted | (13) |
|  | *Bacteroides cellulosilyticus* | Bacteroidetes | Predicted | (13) |
|  | *Bacteroides dorei* | Bacteroidetes | Predicted | (13) |
|  | *Bacteroides faecichinchillae* | Bacteroidetes | Predicted | (13) |
|  | *Bacteroides faecis* | Bacteroidetes | Predicted | (13) |
|  | *Bacteroides finegoldii* | Bacteroidetes | Predicted | (13) |
|  | *Bacteroides fragilis* | Bacteroidetes | Predicted | (13) |
|  | *Bacteroides nordii* | Bacteroidetes | Predicted | (13) |
|  | *Bacteroides oleiciplenus* | Bacteroidetes | Predicted | (13) |
|  | *Bacteroides ovatus* | Bacteroidetes | Predicted | (13) |
|  | *Bacteroides salyersiae* | Bacteroidetes | Predicted | (13) |
|  | *Bacteroides stercorirosoris* | Bacteroidetes | Predicted | (13) |
|  | *Bacteroides stercoris* | Bacteroidetes | Predicted | (13) |
|  | *Bacteroides thetaiotaomicron* | Bacteroidetes | Predicted | (13) |
|  | *Bacteroides uniformis* | Bacteroidetes | Predicted | (13) |
|  | *Bacteroides vulgatus* | Bacteroidetes | Predicted | (13) |
|  | *Bacteroides xylanisolvens* | Bacteroidetes | Predicted | (13) |
|  | *Barnesiella intestinihominis* | Bacteroidetes | Predicted | (13) |
|  | *Butyricimonas virosa* | Bacteroidetes | Predicted | (13) |
|  | *Coprobacter fastidiosus* | Bacteroidetes | Predicted | (13) |
|  | *Muribaculum intestinale* | Bacteroidetes | Predicted | (13) |
|  | *Odoribacter splanchnicus* | Bacteroidetes | Predicted | (13) |
|  | *Parabacteroides distasonis* | Bacteroidetes | Predicted | (13) |
|  | *Parabacteroides goldsteinii* | Bacteroidetes | Predicted | (13) |
|  | *Parabacteroides gordonii* | Bacteroidetes | Predicted | (13) |
|  | *Parabacteroides merdae* | Bacteroidetes | Predicted | (13) |
|  | *Paraprevotella clara* | Bacteroidetes | Predicted | (13) |
|  | *Tidjanibacter massiliensis* | Bacteroidetes | Predicted | (13) |
|  | *Anaerofustis stercorihominis DSM 17244* | Firmicutes | Predicted | (10) |
|  | *Anaerostipes caccae DSM 14662* | Firmicutes | Predicted | (10) |
|  | *Anaerostipes sp. 3_2_56FAA* | Firmicutes | Predicted | (10) |
|  | *Bacillus subtilis subsp. subtilis str. 168* | Firmicutes | Predicted | (10) |
|  | *Blautia hansenii DSM 20583* | Firmicutes | Predicted | (10) |
|  | *Butyrivibrio fibrisolvens 16/4* | Firmicutes | Predicted | (10) |
|  | *Catenibacterium mitsuokai DSM 15897* | Firmicutes | Predicted | (10) |
|  | *Clostridium bartlettii DSM 16795* | Firmicutes | Predicted | (10) |
|  | *Clostridium bolteae ATCC BAA-613* | Firmicutes | Predicted | (10) |
|  | *Clostridium clostridioforme 2_1_49FAA* | Firmicutes | Predicted | (10) |
|  | *Clostridium difficile CD196* | Firmicutes | Predicted and experimentally proven | (10) |
|  | *Clostridium difficile NAP07* | Firmicutes | Predicted and experimentally proven | (10) |
|  | *Clostridium difficile NAP08* | Firmicutes | Predicted and experimentally proven | (10) |
|  | *Clostridium hiranonis DSM 13275* | Firmicutes | Predicted | (10) |
|  | *Clostridium hylemonae DSM 15053* | Firmicutes | Predicted | (10) |
|  | *Clostridium leptum DSM 753* | Firmicutes | Predicted | (10) |
|  | *Clostridium methylpentosum DSM 5476* | Firmicutes | Predicted | (10) |
|  | *Clostridium nexile DSM 1787* | Firmicutes | Predicted | (10) |
|  | *Clostridium ramosum DSM 1402* | Firmicutes | Predicted | (10) |
|  | *Clostridium scindens ATCC 35704* | Firmicutes | Predicted | (10) |
|  | *Clostridium sp. L2-50* | Firmicutes | Predicted | (10) |
|  | *Clostridium sp. SS2/1* | Firmicutes | Predicted | (10) |
|  | *Clostridium spiroforme DSM 1552* | Firmicutes | Predicted | (10) |
|  | *Clostridium sporogenes ATCC 15579* | Firmicutes | Predicted | (10) |
|  | *Clostridium symbiosum WAL-14163* | Firmicutes | Predicted | (10) |
|  | *Clostridium symbiosum WAL-14673* | Firmicutes | Predicted | (10) |
|  | *Coprococcus comes ATCC 27758* | Firmicutes | Predicted | (10) |
|  | *Coprococcus eutactus ATCC 27759* | Firmicutes | Predicted | (10) |
|  | *Dorea longicatena DSM 13814* | Firmicutes | Predicted | (10) |
|  | *Erysipelotrichaceae bacterium 3_1_53* | Firmicutes | Predicted | (10) |
|  | *Eubacterium biforme DSM 3989* | Firmicutes | Predicted | (10) |
|  | *Eubacterium hallii DSM 3353* | Firmicutes | Predicted | (10) |
|  | *Eubacterium rectale DSM 17629* | Firmicutes | Predicted | (10) |
|  | *Eubacterium rectale M104/1* | Firmicutes | Predicted | (10) |
|  | *Eubacterium siraeum 70/3* | Firmicutes | Predicted | (10) |
|  | *Eubacterium siraeum DSM 15702* | Firmicutes | Predicted | (10) |
|  | *Eubacterium ventriosum ATCC 27560* | Firmicutes | Predicted | (10) |
|  | *Lachnospiraceae bacterium 5_1_63FAA* | Firmicutes | Predicted | (10) |
|  | *Lachnospiraceae bacterium 8_1_57FAA* | Firmicutes | Predicted | (10) |
|  | *Lactobacillus brevis subsp. gravesensis ATCC 27305* | Firmicutes | Predicted | (10) |
|  | *Lactobacillus ruminis ATCC 25644* | Firmicutes | Predicted | (10) |
|  | *Listeria monocytogenes str. 1/2a F6854* | Firmicutes | Predicted and experimentally proven | (10) |
|  | *Megamonas hypermegale ART12/1* | Firmicutes | Predicted | (10) |
|  | *Roseburia intestinalis L1-82* | Firmicutes | Predicted | (10) |
|  | *Roseburia inulinivorans DSM 16841* | Firmicutes | Predicted | (10) |
|  | *Ruminococcus bromii L2-63* | Firmicutes | Predicted | (10) |
|  | *Ruminococcus gnavus ATCC 29149* | Firmicutes | Predicted | (10) |
|  | *Ruminococcus lactaris ATCC 29176* | Firmicutes | Predicted | (10) |
|  | *Ruminococcus obeum A2-162* | Firmicutes | Predicted | (10) |
|  | *Ruminococcus sp. 18P13* | Firmicutes | Predicted | (10) |
|  | *Ruminococcus sp. 5_1_39BFAA* | Firmicutes | Predicted | (10) |
|  | *Ruminococcus sp. SR1/5* | Firmicutes | Predicted | (10) |
|  | *Ruminococcus torques ATCC 27756* | Firmicutes | Predicted | (10) |
|  | *Ruminococcus torques L2-14* | Firmicutes | Predicted | (10) |
|  | *Streptococcus infantarius subsp. infantarius ATCC BAA-102* | Firmicutes | Predicted | (10) |
|  | *[Clostridium] cocleatum* | Firmicutes | Predicted | (13) |
|  | *[Clostridium] glycyrrhizinilyticum* | Firmicutes | Predicted | (13) |
|  | *[Clostridium] hylemonae* | Firmicutes | Predicted | (13) |
|  | *[Clostridium] leptum* | Firmicutes | Predicted | (13) |
|  | *[Clostridium] spiroforme* | Firmicutes | Predicted | (13) |
|  | *Bariatricus massiliensis* | Firmicutes | Predicted | (13) |
|  | *Erysipelatoclostridium ramosum* | Firmicutes | Predicted | (13) |
|  | *Eubacterium coprostanoligenes* | Firmicutes | Predicted | (13) |
|  | *Eubacterium ventriosum* | Firmicutes | Predicted | (13) |
|  | *Fusicatenibacter saccharivorans* | Firmicutes | Predicted | (13) |
|  | *Gemmiger formicilis* | Firmicutes | Predicted | (13) |
|  | *Ruminococcus champanellensis* | Firmicutes | Predicted | (13) |
|  | *Sellimonas intestinalis* | Firmicutes | Predicted | (13) |
|  | *Tyzzerella nexilis* | Firmicutes | Predicted | (13) |
|  | *[Clostridium] methylpentosum* | Firmicutes | Predicted | (13) |
|  | *[Clostridium] scindens* | Firmicutes | Predicted | (13) |
|  | *Anaerofustis stercorihominis* | Firmicutes | Predicted | (13) |
|  | *Blautia glucerasea* | Firmicutes | Predicted | (13) |
|  | *Pseudobutyrivibrio ruminis* | Firmicutes | Predicted | (13) |
|  | *Ruminococcus faecis* | Firmicutes | Predicted | (13) |
|  | *[Ruminococcus] torques* | Firmicutes | Predicted | (13) |
|  | *[Clostridium] innocuum* | Firmicutes | Predicted | (13) |
|  | *Blautia obeum* | Firmicutes | Predicted | (13) |
|  | *[Clostridium] clostridioforme* | Firmicutes | Predicted | (13) |
|  | *[Clostridium] symbiosum* | Firmicutes | Predicted | (13) |
|  | *[Clostridium] viride* | Firmicutes | Predicted | (13) |
|  | *Clostridium saudiense* | Firmicutes | Predicted | (13) |
|  | *Coprococcus catus* | Firmicutes | Predicted | (13) |
|  | *Faecalicatena contorta* | Firmicutes | Predicted | (13) |
|  | *Holdemanella biformis* | Firmicutes | Predicted | (13) |
|  | *Sporobacter termitidis* | Firmicutes | Predicted | (13) |
|  | *[Eubacterium] eligens* | Firmicutes | Predicted | (13) |
|  | *[Eubacterium] hallii* | Firmicutes | Predicted | (13) |
|  | *[Eubacterium] rectale* | Firmicutes | Predicted | (13) |
|  | *Anaerobium acetethylicum* | Firmicutes | Predicted | (13) |
|  | *Anaerostipes hadrus* | Firmicutes | Predicted | (13) |
|  | *Anaerotignum propionicum* | Firmicutes | Predicted | (13) |
|  | *Blautia luti* | Firmicutes | Predicted | (13) |
|  | *Blautia wexlerae* | Firmicutes | Predicted | (13) |
|  | *Clostridium perfringens* | Firmicutes | Predicted | (13) |
|  | *Coprococcus comes* | Firmicutes | Predicted | (13) |
|  | *Coprococcus eutactus* | Firmicutes | Predicted | (13) |
|  | *Dorea longicatena* | Firmicutes | Predicted | (13) |
|  | *Eubacterium callanderi* | Firmicutes | Predicted | (13) |
|  | *Eubacterium limosum* | Firmicutes | Predicted | (13) |
|  | *Eubacterium ramulus* | Firmicutes | Predicted | (13) |
|  | *Peptococcus niger* | Firmicutes | Predicted | (13) |
|  | *Robinsoniella peoriensis* | Firmicutes | Predicted | (13) |
|  | *Roseburia faecis* | Firmicutes | Predicted | (13) |
|  | *Ruminococcus albus* | Firmicutes | Predicted | (13) |
|  | *Ruminococcus gauvreauii* | Firmicutes | Predicted | (13) |
|  | *Fusobacterium mortiferum ATCC 9817* | Fusobacteria | Predicted | (10) |
|  | *Fusobacterium sp. 1_1_41FAA* | Fusobacteria | Predicted | (10) |
|  | *Fusobacterium sp. 2_1_31* | Fusobacteria | Predicted | (10) |
|  | *Fusobacterium sp. 3_1_27* | Fusobacteria | Predicted | (10) |
|  | *Fusobacterium sp. 3_1_33* | Fusobacteria | Predicted | (10) |
|  | *Fusobacterium sp. 3_1_36A2* | Fusobacteria | Predicted | (10) |
|  | *Fusobacterium sp. 4_1_13* | Fusobacteria | Predicted | (10) |
|  | *Fusobacterium sp. 7_1* | Fusobacteria | Predicted | (10) |
|  | *Fusobacterium sp. D11* | Fusobacteria | Predicted | (10) |
|  | *Fusobacterium sp. D12* | Fusobacteria | Predicted | (10) |
|  | *Fusobacterium ulcerans ATCC 49185* | Fusobacteria | Predicted | (10) |
|  | *Fusobacterium varium ATCC 27725* | Fusobacteria | Predicted | (10) |
|  | *Victivallis vadensis* | Lentisphaerae | Predicted | (13) |
|  | *Acinetobacter junii SH205* | Proteobacteria | Predicted | (10) |
|  | *Bilophila wadsworthia 3_1_6* | Proteobacteria | Predicted | (10) |
|  | *Citrobacter sp. 30_2* | Proteobacteria | Predicted | (10) |
|  | *Citrobacter youngae ATCC 29220* | Proteobacteria | Predicted | (10) |
|  | *Desulfovibrio piger ATCC 29098* | Proteobacteria | Predicted | (10) |
|  | *Desulfovibrio sp. 3_1_syn3* | Proteobacteria | Predicted | (10) |
|  | *Enterobacter cancerogenus ATCC 35316* | Proteobacteria | Predicted | (10) |
|  | *Enterobacteriaceae bacterium 9_2_54FAA* | Proteobacteria | Predicted | (10) |
|  | *Escherichia coli O157:H7 str. Sakai* | Proteobacteria | Predicted | (10) |
|  | *Escherichia coli SE11* | Proteobacteria | Predicted | (10) |
|  | *Escherichia coli str. K-12 substr. MG1655* | Proteobacteria | Predicted and experimentally proven | (10) |
|  | *Escherichia coli UTI89* | Proteobacteria | Predicted | (10) |
|  | *Escherichia sp. 3_2_53FAA* | Proteobacteria | Predicted | (10) |
|  | *Escherichia sp. 4_1_40B* | Proteobacteria | Predicted | (10) |
|  | *Helicobacter canadensis MIT 98-5491* | Proteobacteria | Predicted | (10) |
|  | *Helicobacter cinaedi CCUG 18818* | Proteobacteria | Predicted | (10) |
|  | *Helicobacter pullorum MIT 98-5489* | Proteobacteria | Predicted | (10) |
|  | *Helicobacter pylori 26695* | Proteobacteria | Predicted and experimentally proven | (10) |
|  | *Helicobacter winghamensis ATCC BAA-430* | Proteobacteria | Predicted | (10) |
|  | *Klebsiella pneumoniae 1162281* | Proteobacteria | Predicted and experimentally proven | (10) |
|  | *Klebsiella sp. 1_1_55* | Proteobacteria | Predicted | (10) |
|  | *Oxalobacter formigenes HOxBLS* | Proteobacteria | Predicted | (10) |
|  | *Oxalobacter formigenes OXCC13* | Proteobacteria | Predicted | (10) |
|  | *Providencia alcalifaciens DSM 30120* | Proteobacteria | Predicted | (10) |
|  | *Providencia rettgeri DSM 1131* | Proteobacteria | Predicted | (10) |
|  | *Providencia rustigianii DSM 4541* | Proteobacteria | Predicted | (10) |
|  | *Providencia stuartii ATCC 25827* | Proteobacteria | Predicted | (10) |
|  | *Ralstonia sp. 5_7_47FAA* | Proteobacteria | Predicted | (10) |
|  | *Salmonella enterica subsp. enterica serovar Typhimurium str.* | Proteobacteria | Predicted and experimentally proven | (10) |
|  | *Rhodospirillum rubrum* | Proteobacteria | Predicted | (13) |
|  | *Desulfovibrio piger* | Proteobacteria | Predicted | (13) |
|  | *Escherichia fergusonii* | Proteobacteria | Predicted | (13) |
|  | *Akkermansia muciniphila* | Verrucomicrobiota | Predicted | (13) |
|  |  |  |  |  |
|  |  |  |  |  |
|  |  |  |  |  |
|  |  |  |  |  |
|  |  |  |  |  |
|  |  |  |  |  |
| **Pantothenate (B5)** | *Actinomyces odontolyticus ATCC 17982* | Actinobacteria | Predicted | (10) |
|  | *Eggerthella sp. 1_3_56FAA* | Actinobacteria | Predicted | (10) |
|  | *Corynebacterium ammoniagenes DSM 20306* | Actinobacteria | Predicted | (10) |
|  | *Adlercreutzia equolifaciens* | Actinomycetota | Predicted | (13) |
|  | *Corynebacterium pseudodiphtheriticum* | Actinomycetota | Predicted | (13) |
|  | *Prevotella salivae DSM 15606* | Bacteroidetes | Predicted | (10) |
|  | *Alistipes indistinctus YIT 12060* | Bacteroidetes | Predicted | (10) |
|  | *Bacteroides caccae ATCC 43185* | Bacteroidetes | Predicted | (10) |
|  | *Bacteroides cellulosilyticus* | Bacteroidetes | Predicted | (13) |
|  | *Bacteroides cellulosilyticus DSM 14838* | Bacteroidetes | Predicted | (10) |
|  | *Bacteroides coprocola DSM 17136* | Bacteroidetes | Predicted | (10) |
|  | *Bacteroides coprophilus DSM 18228* | Bacteroidetes | Predicted | (10) |
|  | *Bacteroides dorei DSM 17855* | Bacteroidetes | Predicted | (10) |
|  | *Bacteroides eggerthii 1_2_48FAA* | Bacteroidetes | Predicted | (10) |
|  | *Bacteroides eggerthii DSM 20697* | Bacteroidetes | Predicted | (10) |
|  | *Bacteroides finegoldii DSM 17565* | Bacteroidetes | Predicted | (10) |
|  | *Bacteroides fragilis* | Bacteroidetes | Predicted | (13) |
|  | *Bacteroides fragilis 3_1_12* | Bacteroidetes | Predicted | (10) |
|  | *Bacteroides fragilis NCTC 9343* | Bacteroidetes | Predicted and experimentally proven | (10) |
|  | *Bacteroides fragilis YCH46* | Bacteroidetes | Predicted | (10) |
|  | *Bacteroides intestinalis DSM 17393* | Bacteroidetes | Predicted | (10) |
|  | *Bacteroides ovatus ATCC 8483* | Bacteroidetes | Predicted | (10) |
|  | *Bacteroides ovatus SD CC 2a* | Bacteroidetes | Predicted | (10) |
|  | *Bacteroides ovatus SD CMC 3f* | Bacteroidetes | Predicted | (10) |
|  | *Bacteroides plebeius DSM 17135* | Bacteroidetes | Predicted | (10) |
|  | *Bacteroides sp. 1_1_30* | Bacteroidetes | Predicted | (10) |
|  | *Bacteroides sp. 1_1_6* | Bacteroidetes | Predicted | (10) |
|  | *Bacteroides sp. 2_1_16* | Bacteroidetes | Predicted | (10) |
|  | *Bacteroides sp. 2_1_22* | Bacteroidetes | Predicted | (10) |
|  | *Bacteroides sp. 2_1_33B* | Bacteroidetes | Predicted | (10) |
|  | *Bacteroides sp. 2_1_7* | Bacteroidetes | Predicted | (10) |
|  | *Bacteroides sp. 2_2_4* | Bacteroidetes | Predicted | (10) |
|  | *Bacteroides sp. 3_1_19* | Bacteroidetes | Predicted | (10) |
|  | *Bacteroides sp. 3_1_23* | Bacteroidetes | Predicted | (10) |
|  | *Bacteroides sp. 3_1_33FAA* | Bacteroidetes | Predicted | (10) |
|  | *Bacteroides sp. 3_1_40A* | Bacteroidetes | Predicted | (10) |
|  | *Bacteroides sp. 3_2_5* | Bacteroidetes | Predicted | (10) |
|  | *Bacteroides sp. 4_1_36* | Bacteroidetes | Predicted | (10) |
|  | *Bacteroides sp. 4_3_47FAA* | Bacteroidetes | Predicted | (10) |
|  | *Bacteroides sp. 9_1_42FAA* | Bacteroidetes | Predicted | (10) |
|  | *Bacteroides sp. D1* | Bacteroidetes | Predicted | (10) |
|  | *Bacteroides sp. D2* | Bacteroidetes | Predicted | (10) |
|  | *Bacteroides sp. D20* | Bacteroidetes | Predicted | (10) |
|  | *Bacteroides sp. D22* | Bacteroidetes | Predicted | (10) |
|  | *Bacteroides stercoris* | Bacteroidetes | Predicted | (13) |
|  | *Bacteroides stercoris ATCC 43183* | Bacteroidetes | Predicted | (10) |
|  | *Bacteroides thetaiotaomicron CL09T03C10* | Bacteroidetes | Predicted | (10) |
|  | *Bacteroides thetaiotaomicron dnLKV9* | Bacteroidetes | Predicted | (10) |
|  | *Bacteroides thetaiotaomicron VPI-5482* | Bacteroidetes | Predicted and experimentally proven | (10) |
|  | *Bacteroides uniformis* | Bacteroidetes | Predicted | (13) |
|  | *Bacteroides uniformis ATCC 8492* | Bacteroidetes | Predicted | (10) |
|  | *Bacteroides vulgatus* | Bacteroidetes | Predicted | (13) |
|  | *Bacteroides vulgatus ATCC 8482* | Bacteroidetes | Predicted and experimentally proven | (10) |
|  | *Bacteroides vulgatus PC510* | Bacteroidetes | Predicted | (10) |
|  | *Bacteroides xylanisolvens SD CC 1b* | Bacteroidetes | Predicted | (10) |
|  | *Bacteroides xylanisolvens XB1A* | Bacteroidetes | Predicted | (10) |
|  | *Parabacteroides goldsteinii* | Bacteroidetes | Predicted | (13) |
|  | *Parabacteroides distasonis* | Bacteroidetes | Predicted | (10,13) |
|  | *Parabacteroides johnsonii DSM 18315* | Bacteroidetes | Predicted | (10) |
|  | *Parabacteroides merdae* | Bacteroidetes | Predicted | (13) |
|  | *Parabacteroides merdae ATCC 43184* | Bacteroidetes | Predicted | (10) |
|  | *Parabacteroides sp. D13* | Bacteroidetes | Predicted | (10) |
|  | *Prevotella copri DSM 18205* | Bacteroidetes | Predicted | (10) |
|  | *Alistipes finegoldii* | Bacteroidetes | Predicted | (13) |
|  | *Alistipes obesi* | Bacteroidetes | Predicted | (13) |
|  | *Alistipes onderdonkii* | Bacteroidetes | Predicted | (13) |
|  | *Alistipes putredinis* | Bacteroidetes | Predicted | (13) |
|  | *Alistipes shahii* | Bacteroidetes | Predicted | (13) |
|  | *Alistipes timonensis* | Bacteroidetes | Predicted | (13) |
|  | *Alistipes ihumii* | Bacteroidetes | Predicted | (13) |
|  | *Alistipes indistinctus* | Bacteroidetes | Predicted | (13) |
|  | *Bacteroides caccae* | Bacteroidetes | Predicted | (13) |
|  | *Bacteroides caecimuris* | Bacteroidetes | Predicted | (13) |
|  | *Bacteroides cellulosilyticus* | Bacteroidetes | Predicted | (13) |
|  | *Bacteroides dorei* | Bacteroidetes | Predicted | (13) |
|  | *Bacteroides faecichinchillae* | Bacteroidetes | Predicted | (13) |
|  | *Bacteroides faecis* | Bacteroidetes | Predicted | (13) |
|  | *Bacteroides finegoldii* | Bacteroidetes | Predicted | (13) |
|  | *Bacteroides fragilis* | Bacteroidetes | Predicted | (13) |
|  | *Bacteroides nordii* | Bacteroidetes | Predicted | (13) |
|  | *Bacteroides oleiciplenus* | Bacteroidetes | Predicted | (13) |
|  | *Bacteroides ovatus* | Bacteroidetes | Predicted | (13) |
|  | *Bacteroides salyersiae* | Bacteroidetes | Predicted | (13) |
|  | *Bacteroides stercorirosoris* | Bacteroidetes | Predicted | (13) |
|  | *Bacteroides stercoris* | Bacteroidetes | Predicted | (13) |
|  | *Bacteroides thetaiotaomicron* | Bacteroidetes | Predicted | (13) |
|  | *Bacteroides uniformis* | Bacteroidetes | Predicted | (13) |
|  | *Bacteroides vulgatus* | Bacteroidetes | Predicted | (13) |
|  | *Bacteroides xylanisolvens* | Bacteroidetes | Predicted | (13) |
|  | *Barnesiella intestinihominis* | Bacteroidetes | Predicted | (13) |
|  | *Butyricimonas virosa* | Bacteroidetes | Predicted | (13) |
|  | *Coprobacter fastidiosus* | Bacteroidetes | Predicted | (13) |
|  | *Muribaculum intestinale* | Bacteroidetes | Predicted | (13) |
|  | *Odoribacter splanchnicus* | Bacteroidetes | Predicted | (13) |
|  | *Parabacteroides distasonis* | Bacteroidetes | Predicted | (13) |
|  | *Parabacteroides goldsteinii* | Bacteroidetes | Predicted | (13) |
|  | *Parabacteroides gordonii* | Bacteroidetes | Predicted | (13) |
|  | *Parabacteroides merdae* | Bacteroidetes | Predicted | (13) |
|  | *Paraprevotella clara* | Bacteroidetes | Predicted | (13) |
|  | *Tidjanibacter massiliensis* | Bacteroidetes | Predicted | (13) |
|  | *Acidaminococcus sp. D21* | Firmicutes | Predicted | (10) |
|  | *Anaerococcus hydrogenalis DSM 7454* | Firmicutes | Predicted | (10) |
|  | *Butyrivibrio crossotus DSM 2876* | Firmicutes | Predicted | (10) |
|  | *Enterococcus faecalis TX0104* | Firmicutes | Predicted | (10) |
|  | *Enterococcus faecalis TX1322* | Firmicutes | Predicted | (10) |
|  | *Enterococcus faecalis TX2134* | Firmicutes | Predicted | (10) |
|  | *Enterococcus faecium DO* | Firmicutes | Predicted | (10) |
|  | *Listeria grayi DSM 20601* | Firmicutes | Predicted | (10) |
|  | *Phascolarctobacterium sp. YIT 12067* | Firmicutes | Predicted | (10) |
|  | *Veillonella sp. 3_1_44* | Firmicutes | Predicted | (10) |
|  | *Veillonella sp. 6_1_27* | Firmicutes | Predicted | (10) |
|  | *Anaerofustis stercorihominis DSM 17244* | Firmicutes | Predicted | (10) |
|  | *Anaerostipes caccae DSM 14662* | Firmicutes | Predicted | (10) |
|  | *Anaerostipes sp. 3_2_56FAA* | Firmicutes | Predicted | (10) |
|  | *Bacillus subtilis subsp. subtilis str. 168* | Firmicutes | Predicted | (10) |
|  | *Butyrivibrio fibrisolvens 16/4* | Firmicutes | Predicted | (10) |
|  | *Clostridium hiranonis DSM 13275* | Firmicutes | Predicted | (10) |
|  | *Clostridium methylpentosum DSM 5476* | Firmicutes | Predicted | (10) |
|  | *Clostridium sp. L2-50* | Firmicutes | Predicted | (10) |
|  | *Clostridium sp. SS2/1* | Firmicutes | Predicted | (10) |
|  | *Clostridium sporogenes ATCC 15579* | Firmicutes | Predicted | (10) |
|  | *Coprococcus comes ATCC 27758* | Firmicutes | Predicted | (10) |
|  | *Coprococcus eutactus ATCC 27759* | Firmicutes | Predicted | (10) |
|  | *Dorea longicatena DSM 13814* | Firmicutes | Predicted | (10) |
|  | *Eubacterium hallii DSM 3353* | Firmicutes | Predicted | (10) |
|  | *Eubacterium rectale DSM 17629* | Firmicutes | Predicted | (10) |
|  | *Eubacterium rectale M104/1* | Firmicutes | Predicted | (10) |
|  | *Eubacterium ventriosum ATCC 27560* | Firmicutes | Predicted | (10) |
|  | *Lachnospiraceae bacterium 5_1_63FAA* | Firmicutes | Predicted | (10) |
|  | *Lachnospiraceae bacterium 8_1_57FAA* | Firmicutes | Predicted | (10) |
|  | *Listeria monocytogenes str. 1/2a F6854* | Firmicutes | Predicted and experimentally proven | (10) |
|  | *Megamonas hypermegale ART12/1* | Firmicutes | Predicted | (10) |
|  | *Roseburia intestinalis L1-82* | Firmicutes | Predicted | (10) |
|  | *Ruminococcus lactaris ATCC 29176* | Firmicutes | Predicted | (10) |
|  | *Ruminococcus sp. 5_1_39BFAA* | Firmicutes | Predicted | (10) |
|  | *Ruminococcus sp. SR1/5* | Firmicutes | Predicted | (10) |
|  | *Ruminococcus torques ATCC 27756* | Firmicutes | Predicted | (10) |
|  | *Ruminococcus torques L2-14* | Firmicutes | Predicted | (10) |
|  | *Streptococcus infantarius subsp. infantarius ATCC BAA-102* | Firmicutes | Predicted | (10) |
|  | *Anaerotignum lactatifermentans* | Firmicutes | Predicted | (13) |
|  | *Eubacterium ventriosum* | Firmicutes | Predicted | (13) |
|  | *Gemmiger formicilis* | Firmicutes | Predicted | (13) |
|  | *[Clostridium] methylpentosum* | Firmicutes | Predicted | (13) |
|  | *Anaerofustis stercorihominis* | Firmicutes | Predicted | (13) |
|  | *Clostridium saudiense* | Firmicutes | Predicted | (13) |
|  | *Sporobacter termitidis* | Firmicutes | Predicted | (13) |
|  | *[Eubacterium] hallii* | Firmicutes | Predicted | (13) |
|  | *[Eubacterium] rectale* | Firmicutes | Predicted | (13) |
|  | *Anaerobium acetethylicum* | Firmicutes | Predicted | (13) |
|  | *Anaerostipes hadrus* | Firmicutes | Predicted | (13) |
|  | *Anaerotignum propionicum* | Firmicutes | Predicted | (13) |
|  | *Coprococcus eutactus* | Firmicutes | Predicted | (13) |
|  | *Eubacterium ramulus* | Firmicutes | Predicted | (13) |
|  | *Peptococcus niger* | Firmicutes | Predicted | (13) |
|  | *Roseburia faecis* | Firmicutes | Predicted | (13) |
|  | *Ruminococcus albus* | Firmicutes | Predicted | (13) |
|  | *Burkholderiales bacterium 1_1_47* | Proteobacteria | Predicted | (10) |
|  | *Campylobacter coli JV20* | Proteobacteria | Predicted | (10) |
|  | *Campylobacter upsaliensis JV21* | Proteobacteria | Predicted | (10) |
|  | *Edwardsiella tarda ATCC 23685* | Proteobacteria | Predicted | (10) |
|  | *Escherichia sp. 1_1_43* | Proteobacteria | Predicted | (10) |
|  | *Helicobacter bilis ATCC 43879* | Proteobacteria | Predicted | (10) |
|  | *Proteus penneri ATCC 35198* | Proteobacteria | Predicted | (10) |
|  | *Acinetobacter junii SH205* | Proteobacteria | Predicted | (10) |
|  | *Bilophila wadsworthia 3_1_6* | Proteobacteria | Predicted | (10) |
|  | *Citrobacter sp. 30_2* | Proteobacteria | Predicted | (10) |
|  | *Citrobacter youngae ATCC 29220* | Proteobacteria | Predicted | (10) |
|  | *Desulfovibrio piger ATCC 29098* | Proteobacteria | Predicted | (10) |
|  | *Desulfovibrio sp. 3_1_syn3* | Proteobacteria | Predicted | (10) |
|  | *Enterobacter cancerogenus ATCC 35316* | Proteobacteria | Predicted | (10) |
|  | *Enterobacteriaceae bacterium 9_2_54FAA* | Proteobacteria | Predicted | (10) |
|  | *Escherichia coli O157:H7 str. Sakai* | Proteobacteria | Predicted | (10) |
|  | *Escherichia coli SE11* | Proteobacteria | Predicted | (10) |
|  | *Escherichia coli str. K-12 substr. MG1655* | Proteobacteria | Predicted and experimentally proven | (10) |
|  | *Escherichia coli UTI89* | Proteobacteria | Predicted | (10) |
|  | *Escherichia sp. 3_2_53FAA* | Proteobacteria | Predicted | (10) |
|  | *Escherichia sp. 4_1_40B* | Proteobacteria | Predicted | (10) |
|  | *Helicobacter canadensis MIT 98-5491* | Proteobacteria | Predicted | (10) |
|  | *Helicobacter cinaedi CCUG 18818* | Proteobacteria | Predicted | (10) |
|  | *Helicobacter pullorum MIT 98-5489* | Proteobacteria | Predicted | (10) |
|  | *Helicobacter pylori 26695* | Proteobacteria | Predicted and experimentally proven | (6,10) |
|  | *Helicobacter winghamensis ATCC BAA-430* | Proteobacteria | Predicted | (10) |
|  | *Klebsiella pneumoniae 1162281* | Proteobacteria | Predicted and experimentally proven | (10) |
|  | *Klebsiella sp. 1_1_55* | Proteobacteria | Predicted | (10) |
|  | *Oxalobacter formigenes HOxBLS* | Proteobacteria | Predicted | (10) |
|  | *Oxalobacter formigenes OXCC13* | Proteobacteria | Predicted | (10) |
|  | *Providencia alcalifaciens DSM 30120* | Proteobacteria | Predicted | (10) |
|  | *Providencia rettgeri DSM 1131* | Proteobacteria | Predicted | (10) |
|  | *Providencia rustigianii DSM 4541* | Proteobacteria | Predicted | (10) |
|  | *Providencia stuartii ATCC 25827* | Proteobacteria | Predicted | (10) |
|  | *Ralstonia sp. 5_7_47FAA* | Proteobacteria | Predicted | (10) |
|  | *Salmonella enterica subsp. enterica serovar Typhimurium str.* | Proteobacteria | Predicted and experimentally proven | (10) |
|  | *Pararhodospirillum photometricum* | Proteobacteria | Predicted | (13) |
|  | *Parasutterella excrementihominis* | Proteobacteria | Predicted | (13) |
|  | *Turicimonas muris* | Proteobacteria | Predicted | (13) |
|  | *Rhodospirillum rubrum* | Proteobacteria | Predicted | (13) |
|  | *Desulfovibrio piger* | Proteobacteria | Predicted | (13) |
|  | *Escherichia fergusonii* | Proteobacteria | Predicted | (13) |
|  | *Akkermansia muciniphila* | Verrucomicrobiota | Predicted | (13) |
|  |  |  |  |  |
|  |  |  |  |  |
|  |  |  |  |  |
|  |  |  |  |  |
|  |  |  |  |  |
|  |  |  |  |  |
|  |  |  |  |  |
| **Pyridoxine (B6)** | *Bifidobacterium adolescentis* | Actinobacteria | Predicted | (10) |
|  | *Bifidobacterium animalis subsp. lactis AD011* | Actinobacteria | Predicted | (10) |
|  | *Bifidobacterium dentium ATCC 27678* | Actinobacteria | Predicted | (10) |
|  | *Bifidobacterium gallicum DSM 20093* | Actinobacteria | Predicted | (10) |
|  | *Bifidobacterium pseudocatenulatum DSM 20438* | Actinobacteria | Predicted | (10) |
|  | *Gordonibacter pamelaeae 7-10-1-b* | Actinobacteria | Predicted | (10) |
|  | *Bifidobacterium angulatum DSM 20098* | Actinobacteria | Predicted | (10) |
|  | *Bifidobacterium bifidum NCIMB 41171* | Actinobacteria | Predicted | (10) |
|  | *Bifidobacterium breve DSM 20213 = JCM 1192* | Actinobacteria | Predicted | (10) |
|  | *Bifidobacterium catenulatum DSM 16992* | Actinobacteria | Predicted | (10) |
|  | *Bifidobacterium longum DJO10A* | Actinobacteria | Predicted | (10) |
|  | *Bifidobacterium longum NCC2705* | Actinobacteria | Predicted | (10) |
|  | *Bifidobacterium longum subsp. infantis 157F* | Actinobacteria | Predicted | (10) |
|  | *Bifidobacterium longum subsp. infantis ATCC 15697* | Actinobacteria | Predicted | (10) |
|  | *Bifidobacterium longum subsp. infantis ATCC 55813* | Actinobacteria | Predicted | (10) |
|  | *Bifidobacterium longum subsp. infantis CCUG 52486* | Actinobacteria | Predicted | (10) |
|  | *Bifidobacterium longum subsp. longum JCM 1217* | Actinobacteria | Predicted | (10) |
|  | *Collinsella aerofaciens ATCC 25986* | Actinobacteria | Predicted | (6,10) |
|  | *Actinomyces odontolyticus ATCC 17982* | Actinobacteria | Predicted | (10) |
|  | *Eggerthella sp. 1_3_56FAA* | Actinobacteria | Predicted | (10) |
|  | *Collinsella aerofaciens* | Actinobacteria | Predicted | (13) |
|  | *Adlercreutzia equolifaciens* | Actinomycetota | Predicted | (13) |
|  | *Corynebacterium pseudodiphtheriticum* | Actinomycetota | Predicted | (13) |
|  | *Prevotella salivae DSM 15606* | Bacteroidetes | Predicted | (10) |
|  | *Alistipes indistinctus YIT 12060* | Bacteroidetes | Predicted | (10) |
|  | *Bacteroides caccae ATCC 43185* | Bacteroidetes | Predicted | (10) |
|  | *Bacteroides cellulosilyticus* | Bacteroidetes | Predicted | (13) |
|  | *Bacteroides cellulosilyticus DSM 14838* | Bacteroidetes | Predicted | (10) |
|  | *Bacteroides dorei DSM 17855* | Bacteroidetes | Predicted | (10) |
|  | *Bacteroides eggerthii 1_2_48FAA* | Bacteroidetes | Predicted | (10) |
|  | *Bacteroides eggerthii DSM 20697* | Bacteroidetes | Predicted | (10) |
|  | *Bacteroides finegoldii DSM 17565* | Bacteroidetes | Predicted | (10) |
|  | *Bacteroides fragilis* | Bacteroidetes | Predicted | (13) |
|  | *Bacteroides fragilis 3_1_12* | Bacteroidetes | Predicted | (10) |
|  | *Bacteroides fragilis NCTC 9343* | Bacteroidetes | Predicted and experimentally proven | (10) |
|  | *Bacteroides fragilis YCH46* | Bacteroidetes | Predicted | (10) |
|  | *Bacteroides intestinalis DSM 17393* | Bacteroidetes | Predicted | (10) |
|  | *Bacteroides ovatus ATCC 8483* | Bacteroidetes | Predicted | (10) |
|  | *Bacteroides ovatus SD CC 2a* | Bacteroidetes | Predicted | (10) |
|  | *Bacteroides ovatus SD CMC 3f* | Bacteroidetes | Predicted | (10) |
|  | *Bacteroides sp. 1_1_30* | Bacteroidetes | Predicted | (10) |
|  | *Bacteroides sp. 1_1_6* | Bacteroidetes | Predicted | (10) |
|  | *Bacteroides sp. 2_1_16* | Bacteroidetes | Predicted | (10) |
|  | *Bacteroides sp. 2_1_22* | Bacteroidetes | Predicted | (10) |
|  | *Bacteroides sp. 2_1_33B* | Bacteroidetes | Predicted | (10) |
|  | *Bacteroides sp. 2_1_7* | Bacteroidetes | Predicted | (10) |
|  | *Bacteroides sp. 2_2_4* | Bacteroidetes | Predicted | (10) |
|  | *Bacteroides sp. 3_1_19* | Bacteroidetes | Predicted | (10) |
|  | *Bacteroides sp. 3_1_23* | Bacteroidetes | Predicted | (10) |
|  | *Bacteroides sp. 3_1_33FAA* | Bacteroidetes | Predicted | (10) |
|  | *Bacteroides sp. 3_1_40A* | Bacteroidetes | Predicted | (10) |
|  | *Bacteroides sp. 3_2_5* | Bacteroidetes | Predicted | (10) |
|  | *Bacteroides sp. 4_1_36* | Bacteroidetes | Predicted | (10) |
|  | *Bacteroides sp. 4_3_47FAA* | Bacteroidetes | Predicted | (10) |
|  | *Bacteroides sp. 9_1_42FAA* | Bacteroidetes | Predicted | (10) |
|  | *Bacteroides sp. D1* | Bacteroidetes | Predicted | (10) |
|  | *Bacteroides sp. D2* | Bacteroidetes | Predicted | (10) |
|  | *Bacteroides sp. D20* | Bacteroidetes | Predicted | (10) |
|  | *Bacteroides sp. D22* | Bacteroidetes | Predicted | (10) |
|  | *Bacteroides stercoris* | Bacteroidetes | Predicted | (13) |
|  | *Bacteroides stercoris ATCC 43183* | Bacteroidetes | Predicted | (10) |
|  | *Bacteroides thetaiotaomicron CL09T03C10* | Bacteroidetes | Predicted | (10) |
|  | *Bacteroides thetaiotaomicron dnLKV9* | Bacteroidetes | Predicted | (10) |
|  | *Bacteroides thetaiotaomicron VPI-5482* | Bacteroidetes | Predicted and experimentally proven | (10) |
|  | *Bacteroides uniformis* | Bacteroidetes | Predicted | (13) |
|  | *Bacteroides uniformis ATCC 8492* | Bacteroidetes | Predicted | (10) |
|  | *Bacteroides vulgatus* | Bacteroidetes | Predicted | (13) |
|  | *Bacteroides vulgatus ATCC 8482* | Bacteroidetes | Predicted and experimentally proven | (10) |
|  | *Bacteroides vulgatus PC510* | Bacteroidetes | Predicted | (10) |
|  | *Bacteroides xylanisolvens SD CC 1b* | Bacteroidetes | Predicted | (10) |
|  | *Bacteroides xylanisolvens XB1A* | Bacteroidetes | Predicted | (10) |
|  | *Parabacteroides goldsteinii* | Bacteroidetes | Predicted | (13) |
|  | *Parabacteroides distasonis* | Bacteroidetes | Predicted | (10,13) |
|  | *Parabacteroides johnsonii DSM 18315* | Bacteroidetes | Predicted | (10) |
|  | *Parabacteroides merdae* | Bacteroidetes | Predicted | (13) |
|  | *Parabacteroides merdae ATCC 43184* | Bacteroidetes | Predicted | (10) |
|  | *Parabacteroides sp. D13* | Bacteroidetes | Predicted | (10) |
|  | *Prevotella copri DSM 18205* | Bacteroidetes | Predicted | (10) |
|  | *Alistipes finegoldii* | Bacteroidetes | Predicted | (13) |
|  | *Alistipes obesi* | Bacteroidetes | Predicted | (13) |
|  | *Alistipes onderdonkii* | Bacteroidetes | Predicted | (13) |
|  | *Alistipes putredinis* | Bacteroidetes | Predicted | (13) |
|  | *Alistipes shahii* | Bacteroidetes | Predicted | (13) |
|  | *Alistipes timonensis* | Bacteroidetes | Predicted | (13) |
|  | *Alistipes ihumii* | Bacteroidetes | Predicted | (13) |
|  | *Alistipes indistinctus* | Bacteroidetes | Predicted | (13) |
|  | *Bacteroides caccae* | Bacteroidetes | Predicted | (13) |
|  | *Bacteroides caecimuris* | Bacteroidetes | Predicted | (13) |
|  | *Bacteroides cellulosilyticus* | Bacteroidetes | Predicted | (13) |
|  | *Bacteroides dorei* | Bacteroidetes | Predicted | (13) |
|  | *Bacteroides faecichinchillae* | Bacteroidetes | Predicted | (13) |
|  | *Bacteroides faecis* | Bacteroidetes | Predicted | (13) |
|  | *Bacteroides finegoldii* | Bacteroidetes | Predicted | (13) |
|  | *Bacteroides fragilis* | Bacteroidetes | Predicted | (13) |
|  | *Bacteroides nordii* | Bacteroidetes | Predicted | (13) |
|  | *Bacteroides oleiciplenus* | Bacteroidetes | Predicted | (13) |
|  | *Bacteroides ovatus* | Bacteroidetes | Predicted | (13) |
|  | *Bacteroides salyersiae* | Bacteroidetes | Predicted | (13) |
|  | *Bacteroides stercorirosoris* | Bacteroidetes | Predicted | (13) |
|  | *Bacteroides stercoris* | Bacteroidetes | Predicted | (13) |
|  | *Bacteroides thetaiotaomicron* | Bacteroidetes | Predicted | (13) |
|  | *Bacteroides uniformis* | Bacteroidetes | Predicted | (13) |
|  | *Bacteroides vulgatus* | Bacteroidetes | Predicted | (13) |
|  | *Bacteroides xylanisolvens* | Bacteroidetes | Predicted | (13) |
|  | *Barnesiella intestinihominis* | Bacteroidetes | Predicted | (13) |
|  | *Butyricimonas virosa* | Bacteroidetes | Predicted | (13) |
|  | *Coprobacter fastidiosus* | Bacteroidetes | Predicted | (13) |
|  | *Muribaculum intestinale* | Bacteroidetes | Predicted | (13) |
|  | *Odoribacter splanchnicus* | Bacteroidetes | Predicted | (13) |
|  | *Parabacteroides distasonis* | Bacteroidetes | Predicted | (13) |
|  | *Parabacteroides goldsteinii* | Bacteroidetes | Predicted | (13) |
|  | *Parabacteroides gordonii* | Bacteroidetes | Predicted | (13) |
|  | *Parabacteroides merdae* | Bacteroidetes | Predicted | (13) |
|  | *Paraprevotella clara* | Bacteroidetes | Predicted | (13) |
|  | *Tidjanibacter massiliensis* | Bacteroidetes | Predicted | (13) |
|  | *Clostridium sp. 7_2_43FAA* | Firmicutes | Predicted | (10) |
|  | *Mitsuokella multacida DSM 20544* | Firmicutes | Predicted | (10) |
|  | *Streptococcus equinus ATCC 9812* | Firmicutes | Predicted | (10) |
|  | *Clostridium bartlettii DSM 16795* | Firmicutes | Predicted | (10) |
|  | *Clostridium leptum DSM 753* | Firmicutes | Predicted | (10) |
|  | *Eubacterium siraeum 70/3* | Firmicutes | Predicted | (10) |
|  | *Eubacterium siraeum DSM 15702* | Firmicutes | Predicted | (10) |
|  | *Lactobacillus brevis subsp. gravesensis ATCC 27305* | Firmicutes | Predicted | (10) |
|  | *Lactobacillus ruminis ATCC 25644* | Firmicutes | Predicted | (10) |
|  | *Ruminococcus gnavus ATCC 29149* | Firmicutes | Predicted | (10) |
|  | *Butyrivibrio crossotus DSM 2876* | Firmicutes | Predicted | (10) |
|  | *Listeria grayi DSM 20601* | Firmicutes | Predicted | (10) |
|  | *Phascolarctobacterium sp. YIT 12067* | Firmicutes | Predicted | (10) |
|  | *Anaerostipes caccae DSM 14662* | Firmicutes | Predicted | (10) |
|  | *Anaerostipes sp. 3_2_56FAA* | Firmicutes | Predicted | (10) |
|  | *Bacillus subtilis subsp. subtilis str. 168* | Firmicutes | Predicted | (10) |
|  | *Clostridium methylpentosum DSM 5476* | Firmicutes | Predicted | (10) |
|  | *Clostridium sp. L2-50* | Firmicutes | Predicted | (10) |
|  | *Clostridium sp. SS2/1* | Firmicutes | Predicted | (10) |
|  | *Clostridium sporogenes ATCC 15579* | Firmicutes | Predicted | (10) |
|  | *Coprococcus eutactus ATCC 27759* | Firmicutes | Predicted | (10) |
|  | *Dorea longicatena DSM 13814* | Firmicutes | Predicted | (10) |
|  | *Eubacterium hallii DSM 3353* | Firmicutes | Predicted | (10) |
|  | *Eubacterium rectale DSM 17629* | Firmicutes | Predicted | (10) |
|  | *Eubacterium rectale M104/1* | Firmicutes | Predicted | (10) |
|  | *Eubacterium ventriosum ATCC 27560* | Firmicutes | Predicted | (10) |
|  | *Lachnospiraceae bacterium 5_1_63FAA* | Firmicutes | Predicted | (10) |
|  | *Listeria monocytogenes str. 1/2a F6854* | Firmicutes | Predicted and experimentally proven | (10) |
|  | *Megamonas hypermegale ART12/1* | Firmicutes | Predicted | (10) |
|  | *Roseburia intestinalis L1-82* | Firmicutes | Predicted | (10) |
|  | *Ruminococcus lactaris ATCC 29176* | Firmicutes | Predicted | (10) |
|  | *Ruminococcus torques L2-14* | Firmicutes | Predicted | (10) |
|  | *Streptococcus infantarius subsp. infantarius ATCC BAA-102* | Firmicutes | Predicted | (10) |
|  | *[Clostridium] asparagiforme* | Firmicutes | Predicted | (13) |
|  | *[Clostridium] lavalense* | Firmicutes | Predicted | (13) |
|  | *[Clostridium] saccharolyticum* | Firmicutes | Predicted | (13) |
|  | *Agathobaculum desmolans* | Firmicutes | Predicted | (13) |
|  | *Anaerofilum pentosovorans* | Firmicutes | Predicted | (13) |
|  | *Anaerotruncus colihominis* | Firmicutes | Predicted | (13) |
|  | *Anaerotruncus rubiinfantis* | Firmicutes | Predicted | (13) |
|  | *Christensenella minuta* | Firmicutes | Predicted | (13) |
|  | *Christensenella timonensis* | Firmicutes | Predicted | (13) |
|  | *Dielma fastidiosa* | Firmicutes | Predicted | (13) |
|  | *Eisenbergiella tayi* | Firmicutes | Predicted | (13) |
|  | *Flavonifractor plautii* | Firmicutes | Predicted | (13) |
|  | *Flintibacter butyricus* | Firmicutes | Predicted | (13) |
|  | *Hungatella hathewayi* | Firmicutes | Predicted | (13) |
|  | *Intestinimonas butyriciproducens* | Firmicutes | Predicted | (13) |
|  | *Lutispora thermophila* | Firmicutes | Predicted | (13) |
|  | *Negativibacillus massiliensis* | Firmicutes | Predicted | (13) |
|  | *Neglecta timonensis* | Firmicutes | Predicted | (13) |
|  | *Phocea massiliensis* | Firmicutes | Predicted | (13) |
|  | *Romboutsia timonensis* | Firmicutes | Predicted | (13) |
|  | *Turicibacter sanguinis* | Firmicutes | Predicted | (13) |
|  | *Blautia schinkii* | Firmicutes | Predicted | (13) |
|  | *Christensenella massiliensis* | Firmicutes | Predicted | (13) |
|  | *Faecalibacterium prausnitzii* | Firmicutes | Predicted | (13) |
|  | *[Clostridium] celerecrescens* | Firmicutes | Predicted | (13) |
|  | *Enterococcus hirae* | Firmicutes | Predicted | (13) |
|  | *Intestinibacillus massiliensis* | Firmicutes | Predicted | (13) |
|  | *Oscillibacter ruminantium* | Firmicutes | Predicted | (13) |
|  | *Oscillibacter valericigenes* | Firmicutes | Predicted | (13) |
|  | *[Clostridium] amygdalinum* | Firmicutes | Predicted | (13) |
|  | *Caecibacter massiliensis* | Firmicutes | Predicted | (13) |
|  | *Emergencia timonensis* | Firmicutes | Predicted | (13) |
|  | *Peptoniphilus grossensis* | Firmicutes | Predicted | (13) |
|  | *[Clostridium] cocleatum* | Firmicutes | Predicted | (13) |
|  | *[Clostridium] glycyrrhizinilyticum* | Firmicutes | Predicted | (13) |
|  | *[Clostridium] hylemonae* | Firmicutes | Predicted | (13) |
|  | *[Clostridium] leptum* | Firmicutes | Predicted | (13) |
|  | *[Clostridium] spiroforme* | Firmicutes | Predicted | (13) |
|  | *Bariatricus massiliensis* | Firmicutes | Predicted | (13) |
|  | *Erysipelatoclostridium ramosum* | Firmicutes | Predicted | (13) |
|  | *Eubacterium coprostanoligenes* | Firmicutes | Predicted | (13) |
|  | *Fusicatenibacter saccharivorans* | Firmicutes | Predicted | (13) |
|  | *Ruminococcus champanellensis* | Firmicutes | Predicted | (13) |
|  | *Sellimonas intestinalis* | Firmicutes | Predicted | (13) |
|  | *[Clostridium] scindens* | Firmicutes | Predicted | (13) |
|  | *Blautia glucerasea* | Firmicutes | Predicted | (13) |
|  | *Pseudobutyrivibrio ruminis* | Firmicutes | Predicted | (13) |
|  | *Ruminococcus faecis* | Firmicutes | Predicted | (13) |
|  | *[Clostridium] innocuum* | Firmicutes | Predicted | (13) |
|  | *Blautia obeum* | Firmicutes | Predicted | (13) |
|  | *[Clostridium] clostridioforme* | Firmicutes | Predicted | (13) |
|  | *[Clostridium] symbiosum* | Firmicutes | Predicted | (13) |
|  | *[Clostridium] viride* | Firmicutes | Predicted | (13) |
|  | *Faecalicatena contorta* | Firmicutes | Predicted | (13) |
|  | *Holdemanella biformis* | Firmicutes | Predicted | (13) |
|  | *[Eubacterium] eligens* | Firmicutes | Predicted | (13) |
|  | *Blautia luti* | Firmicutes | Predicted | (13) |
|  | *Blautia wexlerae* | Firmicutes | Predicted | (13) |
|  | *Clostridium perfringens* | Firmicutes | Predicted | (13) |
|  | *Coprococcus comes* | Firmicutes | Predicted | (13) |
|  | *Dorea longicatena* | Firmicutes | Predicted | (13) |
|  | *Eubacterium callanderi* | Firmicutes | Predicted | (13) |
|  | *Eubacterium limosum* | Firmicutes | Predicted | (13) |
|  | *Robinsoniella peoriensis* | Firmicutes | Predicted | (13) |
|  | *Ruminococcus gauvreauii* | Firmicutes | Predicted | (13) |
|  | *[Ruminococcus] torques* | Firmicutes | Predicted | (13) |
|  | *Anaerotignum lactatifermentans* | Firmicutes | Predicted | (13) |
|  | *Eubacterium ventriosum* | Firmicutes | Predicted | (13) |
|  | *Gemmiger formicilis* | Firmicutes | Predicted | (13) |
|  | *[Clostridium] methylpentosum* | Firmicutes | Predicted | (13) |
|  | *Anaerofustis stercorihominis* | Firmicutes | Predicted | (13) |
|  | *Clostridium saudiense* | Firmicutes | Predicted | (13) |
|  | *Sporobacter termitidis* | Firmicutes | Predicted | (13) |
|  | *[Eubacterium] hallii* | Firmicutes | Predicted | (13) |
|  | *[Eubacterium] rectale* | Firmicutes | Predicted | (13) |
|  | *Anaerobium acetethylicum* | Firmicutes | Predicted | (13) |
|  | *Anaerostipes hadrus* | Firmicutes | Predicted | (13) |
|  | *Anaerotignum propionicum* | Firmicutes | Predicted | (13) |
|  | *Coprococcus eutactus* | Firmicutes | Predicted | (13) |
|  | *Eubacterium ramulus* | Firmicutes | Predicted | (13) |
|  | *Peptococcus niger* | Firmicutes | Predicted | (13) |
|  | *Roseburia faecis* | Firmicutes | Predicted | (13) |
|  | *Ruminococcus albus* | Firmicutes | Predicted | (13) |
|  | *Fusobacterium mortiferum ATCC 9817* | Fusobacteria | Predicted | (10) |
|  | *Fusobacterium ulcerans ATCC 49185* | Fusobacteria | Predicted | (10) |
|  | *Fusobacterium varium ATCC 27725* | Fusobacteria | Predicted | (10) |
|  | *Victivallis vadensis* | Lentisphaerae | Predicted | (13) |
|  | *Succinatimonas hippei YIT 12066* | Proteobacteria | Predicted | (10) |
|  | *Edwardsiella tarda ATCC 23685* | Proteobacteria | Predicted | (10) |
|  | *Proteus penneri ATCC 35198* | Proteobacteria | Predicted | (10) |
|  | *Acinetobacter junii SH205* | Proteobacteria | Predicted | (10) |
|  | *Bilophila wadsworthia 3_1_6* | Proteobacteria | Predicted | (10) |
|  | *Citrobacter sp. 30_2* | Proteobacteria | Predicted | (10) |
|  | *Citrobacter youngae ATCC 29220* | Proteobacteria | Predicted | (10) |
|  | *Desulfovibrio piger ATCC 29098* | Proteobacteria | Predicted | (10) |
|  | *Desulfovibrio sp. 3_1_syn3* | Proteobacteria | Predicted | (10) |
|  | *Enterobacter cancerogenus ATCC 35316* | Proteobacteria | Predicted | (10) |
|  | *Enterobacteriaceae bacterium 9_2_54FAA* | Proteobacteria | Predicted | (10) |
|  | *Escherichia coli O157:H7 str. Sakai* | Proteobacteria | Predicted | (10) |
|  | *Escherichia coli SE11* | Proteobacteria | Predicted | (10) |
|  | *Escherichia coli str. K-12 substr. MG1655* | Proteobacteria | Predicted and experimentally proven | (10) |
|  | *Escherichia coli UTI89* | Proteobacteria | Predicted | (10) |
|  | *Escherichia sp. 3_2_53FAA* | Proteobacteria | Predicted | (10) |
|  | *Escherichia sp. 4_1_40B* | Proteobacteria | Predicted | (10) |
|  | *Klebsiella pneumoniae 1162281* | Proteobacteria | Predicted and experimentally proven | (10) |
|  | *Klebsiella sp. 1_1_55* | Proteobacteria | Predicted | (10) |
|  | *Providencia alcalifaciens DSM 30120* | Proteobacteria | Predicted | (10) |
|  | *Providencia rettgeri DSM 1131* | Proteobacteria | Predicted | (10) |
|  | *Providencia rustigianii DSM 4541* | Proteobacteria | Predicted | (10) |
|  | *Providencia stuartii ATCC 25827* | Proteobacteria | Predicted | (10) |
|  | *Ralstonia sp. 5_7_47FAA* | Proteobacteria | Predicted | (10) |
|  | *Salmonella enterica subsp. enterica serovar Typhimurium str.* | Proteobacteria | Predicted and experimentally proven | (10) |
|  | *Pararhodospirillum photometricum* | Proteobacteria | Predicted | (13) |
|  | *Parasutterella excrementihominis* | Proteobacteria | Predicted | (13) |
|  | *Turicimonas muris* | Proteobacteria | Predicted | (13) |
|  | *Rhodospirillum rubrum* | Proteobacteria | Predicted | (13) |
|  | *Desulfovibrio piger* | Proteobacteria | Predicted | (13) |
|  | *Escherichia fergusonii* | Proteobacteria | Predicted | (13) |
|  | *Akkermansia muciniphila* | Verrucomicrobiota | Predicted | (13) |
|  |  |  |  |  |
|  |  |  |  |  |
|  |  |  |  |  |
|  |  |  |  |  |
|  |  |  |  |  |
| **Biotin (B7)** | *Corynebacterium pseudodiphtheriticum* | Actinomycetota | Predicted | (13) |
|  | *Bacteroides coprocola DSM 17136* | Bacteroidetes | Predicted | (10) |
|  | *Bacteroides coprophilus DSM 18228* | Bacteroidetes | Predicted | (10) |
|  | *Bacteroides plebeius DSM 17135* | Bacteroidetes | Predicted | (10) |
|  | *Alistipes indistinctus YIT 12060* | Bacteroidetes | Predicted | (10) |
|  | *Bacteroides caccae ATCC 43185* | Bacteroidetes | Predicted | (10) |
|  | *Bacteroides cellulosilyticus* | Bacteroidetes | Predicted | (13) |
|  | *Bacteroides cellulosilyticus DSM 14838* | Bacteroidetes | Predicted | (10) |
|  | *Bacteroides dorei DSM 17855* | Bacteroidetes | Predicted | (10) |
|  | *Bacteroides eggerthii 1_2_48FAA* | Bacteroidetes | Predicted | (10) |
|  | *Bacteroides eggerthii DSM 20697* | Bacteroidetes | Predicted | (10) |
|  | *Bacteroides finegoldii DSM 17565* | Bacteroidetes | Predicted | (10) |
|  | *Bacteroides fragilis* | Bacteroidetes | Predicted | (13) |
|  | *Bacteroides fragilis 3_1_12* | Bacteroidetes | Predicted | (10) |
|  | *Bacteroides fragilis NCTC 9343* | Bacteroidetes | Predicted and experimentally proven | (10) |
|  | *Bacteroides fragilis YCH46* | Bacteroidetes | Predicted | (10) |
|  | *Bacteroides intestinalis DSM 17393* | Bacteroidetes | Predicted | (10) |
|  | *Bacteroides ovatus ATCC 8483* | Bacteroidetes | Predicted | (10) |
|  | *Bacteroides ovatus SD CC 2a* | Bacteroidetes | Predicted | (10) |
|  | *Bacteroides ovatus SD CMC 3f* | Bacteroidetes | Predicted | (10) |
|  | *Bacteroides sp. 1_1_30* | Bacteroidetes | Predicted | (10) |
|  | *Bacteroides sp. 1_1_6* | Bacteroidetes | Predicted | (10) |
|  | *Bacteroides sp. 2_1_16* | Bacteroidetes | Predicted | (10) |
|  | *Bacteroides sp. 2_1_22* | Bacteroidetes | Predicted | (10) |
|  | *Bacteroides sp. 2_1_33B* | Bacteroidetes | Predicted | (10) |
|  | *Bacteroides sp. 2_1_7* | Bacteroidetes | Predicted | (10) |
|  | *Bacteroides sp. 2_2_4* | Bacteroidetes | Predicted | (10) |
|  | *Bacteroides sp. 3_1_19* | Bacteroidetes | Predicted | (10) |
|  | *Bacteroides sp. 3_1_23* | Bacteroidetes | Predicted | (10) |
|  | *Bacteroides sp. 3_1_33FAA* | Bacteroidetes | Predicted | (10) |
|  | *Bacteroides sp. 3_1_40A* | Bacteroidetes | Predicted | (10) |
|  | *Bacteroides sp. 3_2_5* | Bacteroidetes | Predicted | (10) |
|  | *Bacteroides sp. 4_1_36* | Bacteroidetes | Predicted | (10) |
|  | *Bacteroides sp. 4_3_47FAA* | Bacteroidetes | Predicted | (10) |
|  | *Bacteroides sp. 9_1_42FAA* | Bacteroidetes | Predicted | (10) |
|  | *Bacteroides sp. D1* | Bacteroidetes | Predicted | (10) |
|  | *Bacteroides sp. D2* | Bacteroidetes | Predicted | (10) |
|  | *Bacteroides sp. D20* | Bacteroidetes | Predicted | (10) |
|  | *Bacteroides sp. D22* | Bacteroidetes | Predicted | (10) |
|  | *Bacteroides stercoris* | Bacteroidetes | Predicted | (13) |
|  | *Bacteroides stercoris ATCC 43183* | Bacteroidetes | Predicted | (10) |
|  | *Bacteroides thetaiotaomicron CL09T03C10* | Bacteroidetes | Predicted | (10) |
|  | *Bacteroides thetaiotaomicron dnLKV9* | Bacteroidetes | Predicted | (10) |
|  | *Bacteroides thetaiotaomicron VPI-5482* | Bacteroidetes | Predicted and experimentally proven | (10) |
|  | *Bacteroides uniformis* | Bacteroidetes | Predicted | (13) |
|  | *Bacteroides uniformis ATCC 8492* | Bacteroidetes | Predicted | (10) |
|  | *Bacteroides vulgatus* | Bacteroidetes | Predicted | (13) |
|  | *Bacteroides vulgatus ATCC 8482* | Bacteroidetes | Predicted and experimentally proven | (10) |
|  | *Bacteroides vulgatus PC510* | Bacteroidetes | Predicted | (10) |
|  | *Bacteroides xylanisolvens SD CC 1b* | Bacteroidetes | Predicted | (10) |
|  | *Bacteroides xylanisolvens XB1A* | Bacteroidetes | Predicted | (10) |
|  | *Parabacteroides goldsteinii* | Bacteroidetes | Predicted | (13) |
|  | *Parabacteroides distasonis* | Bacteroidetes | Predicted | (10,13) |
|  | *Parabacteroides johnsonii DSM 18315* | Bacteroidetes | Predicted | (10) |
|  | *Parabacteroides merdae* | Bacteroidetes | Predicted | (13) |
|  | *Parabacteroides merdae ATCC 43184* | Bacteroidetes | Predicted | (10) |
|  | *Parabacteroides sp. D13* | Bacteroidetes | Predicted | (10) |
|  | *Alistipes ihumii* | Bacteroidetes | Predicted | (13) |
|  | *Alistipes indistinctus* | Bacteroidetes | Predicted | (13) |
|  | *Bacteroides caccae* | Bacteroidetes | Predicted | (13) |
|  | *Bacteroides caecimuris* | Bacteroidetes | Predicted | (13) |
|  | *Bacteroides cellulosilyticus* | Bacteroidetes | Predicted | (13) |
|  | *Bacteroides dorei* | Bacteroidetes | Predicted | (13) |
|  | *Bacteroides faecichinchillae* | Bacteroidetes | Predicted | (13) |
|  | *Bacteroides faecis* | Bacteroidetes | Predicted | (13) |
|  | *Bacteroides finegoldii* | Bacteroidetes | Predicted | (13) |
|  | *Bacteroides fragilis* | Bacteroidetes | Predicted | (13) |
|  | *Bacteroides nordii* | Bacteroidetes | Predicted | (13) |
|  | *Bacteroides oleiciplenus* | Bacteroidetes | Predicted | (13) |
|  | *Bacteroides ovatus* | Bacteroidetes | Predicted | (13) |
|  | *Bacteroides salyersiae* | Bacteroidetes | Predicted | (13) |
|  | *Bacteroides stercorirosoris* | Bacteroidetes | Predicted | (13) |
|  | *Bacteroides stercoris* | Bacteroidetes | Predicted | (13) |
|  | *Bacteroides thetaiotaomicron* | Bacteroidetes | Predicted | (13) |
|  | *Bacteroides uniformis* | Bacteroidetes | Predicted | (13) |
|  | *Bacteroides vulgatus* | Bacteroidetes | Predicted | (13) |
|  | *Bacteroides xylanisolvens* | Bacteroidetes | Predicted | (13) |
|  | *Barnesiella intestinihominis* | Bacteroidetes | Predicted | (13) |
|  | *Butyricimonas virosa* | Bacteroidetes | Predicted | (13) |
|  | *Coprobacter fastidiosus* | Bacteroidetes | Predicted | (13) |
|  | *Muribaculum intestinale* | Bacteroidetes | Predicted | (13) |
|  | *Odoribacter splanchnicus* | Bacteroidetes | Predicted | (13) |
|  | *Parabacteroides distasonis* | Bacteroidetes | Predicted | (13) |
|  | *Parabacteroides goldsteinii* | Bacteroidetes | Predicted | (13) |
|  | *Parabacteroides gordonii* | Bacteroidetes | Predicted | (13) |
|  | *Parabacteroides merdae* | Bacteroidetes | Predicted | (13) |
|  | *Paraprevotella clara* | Bacteroidetes | Predicted | (13) |
|  | *Ruminococcaceae bacterium D16* | Firmicutes | Predicted | (10) |
|  | *Veillonella sp. 3_1_44* | Firmicutes | Predicted | (10) |
|  | *Veillonella sp. 6_1_27* | Firmicutes | Predicted | (10) |
|  | *Bacillus subtilis subsp. subtilis str. 168* | Firmicutes | Predicted | (10) |
|  | *Megamonas hypermegale ART12/1* | Firmicutes | Predicted | (10) |
|  | *Ruminococcus torques L2-14* | Firmicutes | Predicted | (10) |
|  | *Butyricicoccus pullicaecorum* | Firmicutes | Predicted | (13) |
|  | *[Clostridium] saccharolyticum* | Firmicutes | Predicted | (13) |
|  | *Oscillibacter ruminantium* | Firmicutes | Predicted | (13) |
|  | *[Clostridium] symbiosum* | Firmicutes | Predicted | (13) |
|  | *[Clostridium] viride* | Firmicutes | Predicted | (13) |
|  | *Robinsoniella peoriensis* | Firmicutes | Predicted | (13) |
|  | *Anaerotignum lactatifermentans* | Firmicutes | Predicted | (13) |
|  | *Anaerobium acetethylicum* | Firmicutes | Predicted | (13) |
|  | *Anaerotignum propionicum* | Firmicutes | Predicted | (13) |
|  | *Ruminococcus albus* | Firmicutes | Predicted | (13) |
|  | *Fusobacterium gonidiaformans ATCC 25563* | Fusobacteria | Predicted | (10) |
|  | *Fusobacterium sp. 3_1_5R* | Fusobacteria | Predicted | (10) |
|  | *Fusobacterium sp. 1_1_41FAA* | Fusobacteria | Predicted | (10) |
|  | *Fusobacterium sp. 2_1_31* | Fusobacteria | Predicted | (10) |
|  | *Fusobacterium sp. 3_1_27* | Fusobacteria | Predicted | (10) |
|  | *Fusobacterium sp. 3_1_33* | Fusobacteria | Predicted | (10) |
|  | *Fusobacterium sp. 3_1_36A2* | Fusobacteria | Predicted | (10) |
|  | *Fusobacterium sp. 4_1_13* | Fusobacteria | Predicted | (10) |
|  | *Fusobacterium sp. 7_1* | Fusobacteria | Predicted | (10) |
|  | *Fusobacterium sp. D11* | Fusobacteria | Predicted | (10) |
|  | *Fusobacterium sp. D12* | Fusobacteria | Predicted | (10) |
|  | *Fusobacterium mortiferum ATCC 9817* | Fusobacteria | Predicted | (10) |
|  | *Fusobacterium ulcerans ATCC 49185* | Fusobacteria | Predicted | (10) |
|  | *Fusobacterium varium ATCC 27725* | Fusobacteria | Predicted | (10) |
|  | *Victivallis vadensis* | Lentisphaerae | Predicted | (13) |
|  | *Campylobacter coli JV20* | Proteobacteria | Predicted | (10) |
|  | *Campylobacter upsaliensis JV21* | Proteobacteria | Predicted | (10) |
|  | *Helicobacter bilis ATCC 43879* | Proteobacteria | Predicted | (10) |
|  | *Helicobacter canadensis MIT 98-5491* | Proteobacteria | Predicted | (10) |
|  | *Helicobacter cinaedi CCUG 18818* | Proteobacteria | Predicted | (10) |
|  | *Helicobacter pullorum MIT 98-5489* | Proteobacteria | Predicted | (10) |
|  | *Helicobacter pylori 26695* | Proteobacteria | Predicted and experimentally proven | (10) |
|  | *Helicobacter winghamensis ATCC BAA-430* | Proteobacteria | Predicted | (10) |
|  | *Oxalobacter formigenes HOxBLS* | Proteobacteria | Predicted | (10) |
|  | *Oxalobacter formigenes OXCC13* | Proteobacteria | Predicted | (10) |
|  | *Succinatimonas hippei YIT 12066* | Proteobacteria | Predicted | (10) |
|  | *Edwardsiella tarda ATCC 23685* | Proteobacteria | Predicted | (10) |
|  | *Proteus penneri ATCC 35198* | Proteobacteria | Predicted | (10) |
|  | *Acinetobacter junii SH205* | Proteobacteria | Predicted | (10) |
|  | *Citrobacter sp. 30_2* | Proteobacteria | Predicted | (10) |
|  | *Citrobacter youngae ATCC 29220* | Proteobacteria | Predicted | (10) |
|  | *Enterobacter cancerogenus ATCC 35316* | Proteobacteria | Predicted | (10) |
|  | *Enterobacteriaceae bacterium 9_2_54FAA* | Proteobacteria | Predicted | (10) |
|  | *Escherichia coli O157:H7 str. Sakai* | Proteobacteria | Predicted | (10) |
|  | *Escherichia coli SE11* | Proteobacteria | Predicted | (10) |
|  | *Escherichia coli str. K-12 substr. MG1655* | Proteobacteria | Predicted and experimentally proven | (10) |
|  | *Escherichia coli UTI89* | Proteobacteria | Predicted | (10) |
|  | *Escherichia sp. 3_2_53FAA* | Proteobacteria | Predicted | (10) |
|  | *Escherichia sp. 4_1_40B* | Proteobacteria | Predicted | (10) |
|  | *Klebsiella pneumoniae 1162281* | Proteobacteria | Predicted and experimentally proven | (10) |
|  | *Klebsiella sp. 1_1_55* | Proteobacteria | Predicted | (10) |
|  | *Providencia alcalifaciens DSM 30120* | Proteobacteria | Predicted | (10) |
|  | *Providencia rettgeri DSM 1131* | Proteobacteria | Predicted | (10) |
|  | *Providencia rustigianii DSM 4541* | Proteobacteria | Predicted | (10) |
|  | *Providencia stuartii ATCC 25827* | Proteobacteria | Predicted | (10) |
|  | *Ralstonia sp. 5_7_47FAA* | Proteobacteria | Predicted | (10) |
|  | *Salmonella enterica subsp. enterica serovar Typhimurium str.* | Proteobacteria | Predicted and experimentally proven | (10) |
|  | *Pararhodospirillum photometricum* | Proteobacteria | Predicted | (13) |
|  | *Desulfovibrio piger* | Proteobacteria | Predicted | (13) |
|  | *Escherichia fergusonii* | Proteobacteria | Predicted | (13) |
|  | *Akkermansia muciniphila* | Verrucomicrobiota | Predicted | (13) |
|  |  |  |  |  |
|  |  |  |  |  |
|  |  |  |  |  |
|  |  |  |  |  |
|  |  |  |  |  |
|  |  |  |  |  |
| **Folate (B9)** | *Corynebacterium ammoniagenes DSM 20306* | Actinobacteria | Predicted | (10) |
|  | *Bifidobacterium breve B 622* | Actinobacteria | Experimentally proven | (67)⁠ |
|  | *Bifidobacterium breve MB 234* | Actinobacteria | Experimentally proven | (67)⁠ |
|  | *Bifidobacterium breve MB 235* | Actinobacteria | Experimentally proven | (67)⁠ |
|  | *Bifidobacterium catenulatum DSMZ 16992* | Actinobacteria | Experimentally proven | (67)⁠ |
|  | *Bifidobacterium dentium MB 117* | Actinobacteria | Experimentally proven | (67)⁠ |
|  | *Bifidobacterium infantis ATCC 15697* | Actinobacteria | Experimentally proven | (67)⁠ |
|  | *Bifidobacterium longum MB 214* | Actinobacteria | Experimentally proven | (67)⁠ |
|  | *Bifidobacterium pseudocatenulatum MB 116* | Actinobacteria | Experimentally proven | (67)⁠ |
|  | *Bifidobacterium pseudocatenulatum MB 237* | Actinobacteria | Experimentally proven | (67)⁠ |
|  | *Bifidobacterium pseudocatenulatum MB 264* | Actinobacteria | Experimentally proven | (67)⁠ |
|  | *Bifidobacterium adolescentis MB 239* | Actinobacteria | Experimentally proven | (67)⁠ |
|  | *Bifidobacterium adolescentis* | Actinobacteria | Experimentally proven | (67)⁠ |
|  | *Bifidobacterium adolescentis F 200* | Actinobacteria | Experimentally proven | (67)⁠ |
|  | *Bifidobacterium adolescentis MB 106* | Actinobacteria | Experimentally proven | (67)⁠ |
|  | *Bifidobacterium bifidum* | Actinobacteria | Experimentally proven | (5,67)⁠ |
|  | *Bifidobacterium adolescentis 115* | Actinobacteria | Experimentally proven | (67)⁠ |
|  | *Bifidobacterium adolescentis 227* | Actinobacteria | Experimentally proven | (67)⁠ |
|  | *Bifidobacterium adolescentis MB 114* | Actinobacteria | Experimentally proven | (67)⁠ |
|  | *Bifidobacterium adolescentis DSMZ 20086* | Actinobacteria | Experimentally proven | (67) |
|  | *Bifidobacterium adolescentis* | Actinobacteria | Predicted | (10) |
|  | *Bifidobacterium dentium ATCC 27678* | Actinobacteria | Predicted | (10) |
|  | *Bifidobacterium bifidum NCIMB 41171* | Actinobacteria | Predicted | (10) |
|  | *Bifidobacterium breve DSM 20213 = JCM 1192* | Actinobacteria | Predicted | (10) |
|  | *Bifidobacterium longum subsp. infantis ATCC 15697* | Actinobacteria | Predicted and experimentally proven | (5,10,67)⁠ |
|  | *Collinsella aerofaciens* | Actinobacteria | Predicted | (13) |
|  | *Adlercreutzia equolifaciens* | Actinomycetota | Predicted | (13) |
|  | *Corynebacterium pseudodiphtheriticum* | Actinomycetota | Predicted | (13) |
|  | *Propionibacterium thoenii* | Actinomycetota | Experimentally proven | (68)⁠ |
|  | *Propionibacterium acidipropionici* | Actinomycetota | Experimentally proven | (68) |
|  | *Propionibacterium jensenii* | Actinomycetota | Experimentally proven | (68) |
|  | *Propionibacterium freudenreichii ssp. shermanii* | Actinomycetota | Experimentally proven | (68) |
|  | *Prevotella copri DSM 18205* | Bacteroidetes | Predicted | (10) |
|  | *Bacteroides coprocola DSM 17136* | Bacteroidetes | Predicted | (10) |
|  | *Bacteroides coprophilus DSM 18228* | Bacteroidetes | Predicted | (10) |
|  | *Bacteroides plebeius DSM 17135* | Bacteroidetes | Predicted | (10) |
|  | *Bacteroides caccae ATCC 43185* | Bacteroidetes | Predicted | (10) |
|  | *Bacteroides cellulosilyticus* | Bacteroidetes | Predicted | (13) |
|  | *Bacteroides cellulosilyticus DSM 14838* | Bacteroidetes | Predicted | (10) |
|  | *Bacteroides dorei DSM 17855* | Bacteroidetes | Predicted | (10) |
|  | *Bacteroides eggerthii 1_2_48FAA* | Bacteroidetes | Predicted | (10) |
|  | *Bacteroides eggerthii DSM 20697* | Bacteroidetes | Predicted | (10) |
|  | *Bacteroides fragilis* | Bacteroidetes | Predicted | (13) |
|  | *Bacteroides fragilis 3_1_12* | Bacteroidetes | Predicted | (10) |
|  | *Bacteroides fragilis NCTC 9343* | Bacteroidetes | Predicted and experimentally proven | (10) |
|  | *Bacteroides fragilis YCH46* | Bacteroidetes | Predicted | (10) |
|  | *Bacteroides intestinalis DSM 17393* | Bacteroidetes | Predicted | (10) |
|  | *Bacteroides ovatus ATCC 8483* | Bacteroidetes | Predicted | (10) |
|  | *Bacteroides ovatus SD CC 2a* | Bacteroidetes | Predicted | (10) |
|  | *Bacteroides ovatus SD CMC 3f* | Bacteroidetes | Predicted | (10) |
|  | *Bacteroides sp. 1_1_30* | Bacteroidetes | Predicted | (10) |
|  | *Bacteroides sp. 1_1_6* | Bacteroidetes | Predicted | (10) |
|  | *Bacteroides sp. 2_1_16* | Bacteroidetes | Predicted | (10) |
|  | *Bacteroides sp. 2_1_22* | Bacteroidetes | Predicted | (10) |
|  | *Bacteroides sp. 2_1_33B* | Bacteroidetes | Predicted | (10) |
|  | *Bacteroides sp. 2_1_7* | Bacteroidetes | Predicted | (10) |
|  | *Bacteroides sp. 2_2_4* | Bacteroidetes | Predicted | (10) |
|  | *Bacteroides sp. 3_1_19* | Bacteroidetes | Predicted | (10) |
|  | *Bacteroides sp. 3_1_23* | Bacteroidetes | Predicted | (10) |
|  | *Bacteroides sp. 3_1_33FAA* | Bacteroidetes | Predicted | (10) |
|  | *Bacteroides sp. 3_1_40A* | Bacteroidetes | Predicted | (10) |
|  | *Bacteroides sp. 3_2_5* | Bacteroidetes | Predicted | (10) |
|  | *Bacteroides sp. 4_1_36* | Bacteroidetes | Predicted | (10) |
|  | *Bacteroides sp. 4_3_47FAA* | Bacteroidetes | Predicted | (10) |
|  | *Bacteroides sp. 9_1_42FAA* | Bacteroidetes | Predicted | (10) |
|  | *Bacteroides sp. D1* | Bacteroidetes | Predicted | (10) |
|  | *Bacteroides sp. D2* | Bacteroidetes | Predicted | (10) |
|  | *Bacteroides sp. D20* | Bacteroidetes | Predicted | (10) |
|  | *Bacteroides sp. D22* | Bacteroidetes | Predicted | (10) |
|  | *Bacteroides stercoris* | Bacteroidetes | Predicted | (13) |
|  | *Bacteroides stercoris ATCC 43183* | Bacteroidetes | Predicted | (10) |
|  | *Bacteroides thetaiotaomicron dnLKV9* | Bacteroidetes | Predicted | (10) |
|  | *Bacteroides thetaiotaomicron VPI-5482* | Bacteroidetes | Predicted and experimentally proven | (10) |
|  | *Bacteroides uniformis* | Bacteroidetes | Predicted | (13) |
|  | *Bacteroides uniformis ATCC 8492* | Bacteroidetes | Predicted | (10) |
|  | *Bacteroides vulgatus* | Bacteroidetes | Predicted | (13) |
|  | *Bacteroides vulgatus ATCC 8482* | Bacteroidetes | Predicted and experimentally proven | (10) |
|  | *Bacteroides vulgatus PC510* | Bacteroidetes | Predicted | (10) |
|  | *Bacteroides xylanisolvens SD CC 1b* | Bacteroidetes | Predicted | (10) |
|  | *Bacteroides xylanisolvens XB1A* | Bacteroidetes | Predicted | (10) |
|  | *Parabacteroides goldsteinii* | Bacteroidetes | Predicted | (13) |
|  | *Parabacteroides distasonis* | Bacteroidetes | Predicted | (10,13) |
|  | *Parabacteroides johnsonii DSM 18315* | Bacteroidetes | Predicted | (10) |
|  | *Parabacteroides merdae* | Bacteroidetes | Predicted | (13) |
|  | *Parabacteroides merdae ATCC 43184* | Bacteroidetes | Predicted | (10) |
|  | *Parabacteroides sp. D13* | Bacteroidetes | Predicted | (10) |
|  | *Alistipes finegoldii* | Bacteroidetes | Predicted | (13) |
|  | *Alistipes obesi* | Bacteroidetes | Predicted | (13) |
|  | *Alistipes onderdonkii* | Bacteroidetes | Predicted | (13) |
|  | *Alistipes putredinis* | Bacteroidetes | Predicted | (13) |
|  | *Alistipes shahii* | Bacteroidetes | Predicted | (13) |
|  | *Alistipes timonensis* | Bacteroidetes | Predicted | (13) |
|  | *Tidjanibacter massiliensis* | Bacteroidetes | Predicted | (13) |
|  | *Alistipes ihumii* | Bacteroidetes | Predicted | (13) |
|  | *Alistipes indistinctus* | Bacteroidetes | Predicted | (13) |
|  | *Bacteroides caccae* | Bacteroidetes | Predicted | (13) |
|  | *Bacteroides caecimuris* | Bacteroidetes | Predicted | (13) |
|  | *Bacteroides cellulosilyticus* | Bacteroidetes | Predicted | (13) |
|  | *Bacteroides dorei* | Bacteroidetes | Predicted | (13) |
|  | *Bacteroides faecichinchillae* | Bacteroidetes | Predicted | (13) |
|  | *Bacteroides faecis* | Bacteroidetes | Predicted | (13) |
|  | *Bacteroides finegoldii* | Bacteroidetes | Predicted | (13) |
|  | *Bacteroides fragilis* | Bacteroidetes | Predicted | (13) |
|  | *Bacteroides nordii* | Bacteroidetes | Predicted | (13) |
|  | *Bacteroides oleiciplenus* | Bacteroidetes | Predicted | (13) |
|  | *Bacteroides ovatus* | Bacteroidetes | Predicted | (13) |
|  | *Bacteroides salyersiae* | Bacteroidetes | Predicted | (13) |
|  | *Bacteroides stercorirosoris* | Bacteroidetes | Predicted | (13) |
|  | *Bacteroides stercoris* | Bacteroidetes | Predicted | (13) |
|  | *Bacteroides thetaiotaomicron* | Bacteroidetes | Predicted | (13) |
|  | *Bacteroides uniformis* | Bacteroidetes | Predicted | (13) |
|  | *Bacteroides vulgatus* | Bacteroidetes | Predicted | (13) |
|  | *Bacteroides xylanisolvens* | Bacteroidetes | Predicted | (13) |
|  | *Barnesiella intestinihominis* | Bacteroidetes | Predicted | (13) |
|  | *Butyricimonas virosa* | Bacteroidetes | Predicted | (13) |
|  | *Coprobacter fastidiosus* | Bacteroidetes | Predicted | (13) |
|  | *Muribaculum intestinale* | Bacteroidetes | Predicted | (13) |
|  | *Odoribacter splanchnicus* | Bacteroidetes | Predicted | (13) |
|  | *Parabacteroides distasonis* | Bacteroidetes | Predicted | (13) |
|  | *Parabacteroides goldsteinii* | Bacteroidetes | Predicted | (13) |
|  | *Parabacteroides gordonii* | Bacteroidetes | Predicted | (13) |
|  | *Parabacteroides merdae* | Bacteroidetes | Predicted | (13) |
|  | *Paraprevotella clara* | Bacteroidetes | Predicted | (13) |
|  | *Blautia hydrogenotrophica DSM 10507* | Firmicutes | Predicted | (10) |
|  | *Streptococcus thermophilus* | Firmicutes | Experimentally proven | (6,69)⁠ |
|  | *Lactobacillus lactis subsp.lactis biovar diacetylactis* | Firmicutes | Experimentally proven | (68) |
|  | *Lactobacillus plantarum* | Firmicutes | Experimentally proven | (68) |
|  | *Lactobacillus helveticus* | Firmicutes | Experimentally proven | (68) |
|  | *Lactobacillus acidophilus* | Firmicutes | Experimentally proven | (68) |
|  | *Lactobacillus casei* | Firmicutes | Experimentally proven | (68) |
|  | *Lactobacillus casei subsp. rhamnosus* | Firmicutes | Experimentally proven | (68) |
|  | *Lactobacillus delbrueckii subsp. bulgaricus CRL 863* | Firmicutes | Experimentally proven | (70)⁠ |
|  | *Leuconostoc lactis* | Firmicutes | Experimentally proven | (68) |
|  | *Leuconostoc paramesenteroides* | Firmicutes | Experimentally proven | (68) |
|  | *Lactobacillus lactis subsp. cremoris* | Firmicutes | Experimentally proven | (68) |
|  | *Streptococcus sp. 2_1_36FAA* | Firmicutes | Predicted | (10) |
|  | *Blautia hansenii DSM 20583* | Firmicutes | Predicted | (10) |
|  | *Ruminococcus obeum A2-162* | Firmicutes | Predicted | (10) |
|  | *Clostridium difficile CD196* | Firmicutes | Predicted and experimentally proven | (10) |
|  | *Clostridium difficile NAP07* | Firmicutes | Predicted and experimentally proven | (10) |
|  | *Clostridium difficile NAP08* | Firmicutes | Predicted and experimentally proven | (10) |
|  | *Ruminococcus sp. SR1/5* | Firmicutes | Predicted | (10) |
|  | *Clostridium sp. 7_2_43FAA* | Firmicutes | Predicted | (10) |
|  | *Clostridium bartlettii DSM 16795* | Firmicutes | Predicted | (10) |
|  | *Listeria grayi DSM 20601* | Firmicutes | Predicted | (10) |
|  | *Anaerostipes caccae DSM 14662* | Firmicutes | Predicted | (10) |
|  | *Anaerostipes sp. 3_2_56FAA* | Firmicutes | Predicted | (10) |
|  | *Coprococcus eutactus ATCC 27759* | Firmicutes | Predicted | (10) |
|  | *Lachnospiraceae bacterium 5_1_63FAA* | Firmicutes | Predicted | (10) |
|  | *Listeria monocytogenes str. 1/2a F6854* | Firmicutes | Predicted and experimentally proven | (10) |
|  | *Streptococcus infantarius subsp. infantarius ATCC BAA-102* | Firmicutes | Predicted | (10) |
|  | *Bacillus subtilis subsp. subtilis str. 168* | Firmicutes | Predicted | (10) |
|  | *Coprococcus catus* | Firmicutes | Predicted | (13) |
|  | *Eisenbergiella tayi* | Firmicutes | Predicted | (13) |
|  | *Neglecta timonensis* | Firmicutes | Predicted | (13) |
|  | *Romboutsia timonensis* | Firmicutes | Predicted | (13) |
|  | *Turicibacter sanguinis* | Firmicutes | Predicted | (13) |
|  | *Blautia schinkii* | Firmicutes | Predicted | (13) |
|  | *Caecibacter massiliensis* | Firmicutes | Predicted | (13) |
|  | *Emergencia timonensis* | Firmicutes | Predicted | (13) |
|  | *Peptoniphilus grossensis* | Firmicutes | Predicted | (13) |
|  | *[Clostridium] glycyrrhizinilyticum* | Firmicutes | Predicted | (13) |
|  | *[Clostridium] leptum* | Firmicutes | Predicted | (13) |
|  | *Bariatricus massiliensis* | Firmicutes | Predicted | (13) |
|  | *Eubacterium coprostanoligenes* | Firmicutes | Predicted | (13) |
|  | *Ruminococcus champanellensis* | Firmicutes | Predicted | (13) |
|  | *[Clostridium] scindens* | Firmicutes | Predicted | (13) |
|  | *Blautia glucerasea* | Firmicutes | Predicted | (13) |
|  | *Ruminococcus faecis* | Firmicutes | Predicted | (13) |
|  | *Faecalicatena contorta* | Firmicutes | Predicted | (13) |
|  | *Blautia luti* | Firmicutes | Predicted | (13) |
|  | *Clostridium perfringens* | Firmicutes | Predicted | (13) |
|  | *Coprococcus comes* | Firmicutes | Predicted | (13) |
|  | *Dorea longicatena* | Firmicutes | Predicted | (13) |
|  | *Eubacterium callanderi* | Firmicutes | Predicted | (13) |
|  | *Eubacterium limosum* | Firmicutes | Predicted | (13) |
|  | *Ruminococcus gauvreauii* | Firmicutes | Predicted | (13) |
|  | *Eubacterium ventriosum* | Firmicutes | Predicted | (13) |
|  | *Clostridium saudiense* | Firmicutes | Predicted | (13) |
|  | *Sporobacter termitidis* | Firmicutes | Predicted | (13) |
|  | *[Eubacterium] hallii* | Firmicutes | Predicted | (13) |
|  | *Anaerostipes hadrus* | Firmicutes | Predicted | (13) |
|  | *Coprococcus eutactus* | Firmicutes | Predicted | (13) |
|  | *Eubacterium ramulus* | Firmicutes | Predicted | (13) |
|  | *Peptococcus niger* | Firmicutes | Predicted | (13) |
|  | *Blautia obeum* | Firmicutes | Predicted | (13) |
|  | *Blautia wexlerae* | Firmicutes | Predicted | (13) |
|  | *[Ruminococcus] torques* | Firmicutes | Predicted | (13) |
|  | *[Clostridium] viride* | Firmicutes | Predicted | (13) |
|  | *Robinsoniella peoriensis* | Firmicutes | Predicted | (13) |
|  | *Anaerobium acetethylicum* | Firmicutes | Predicted | (13) |
|  | *Fusobacterium gonidiaformans ATCC 25563* | Fusobacteria | Predicted | (10) |
|  | *Fusobacterium sp. 3_1_5R* | Fusobacteria | Predicted | (10) |
|  | *Fusobacterium sp. 1_1_41FAA* | Fusobacteria | Predicted | (10) |
|  | *Fusobacterium sp. 2_1_31* | Fusobacteria | Predicted | (10) |
|  | *Fusobacterium sp. 3_1_27* | Fusobacteria | Predicted | (10) |
|  | *Fusobacterium sp. 3_1_33* | Fusobacteria | Predicted | (10) |
|  | *Fusobacterium sp. 3_1_36A2* | Fusobacteria | Predicted | (10) |
|  | *Fusobacterium sp. 4_1_13* | Fusobacteria | Predicted | (10) |
|  | *Fusobacterium sp. 7_1* | Fusobacteria | Predicted | (10) |
|  | *Fusobacterium sp. D11* | Fusobacteria | Predicted | (10) |
|  | *Fusobacterium sp. D12* | Fusobacteria | Predicted | (10) |
|  | *Victivallis vadensis* | Lentisphaerae | Predicted | (13) |
|  | *Campylobacter coli JV20* | Proteobacteria | Predicted | (10) |
|  | *Helicobacter bilis ATCC 43879* | Proteobacteria | Predicted | (10) |
|  | *Helicobacter cinaedi CCUG 18818* | Proteobacteria | Predicted | (10) |
|  | *Helicobacter pylori 26695* | Proteobacteria | Predicted and experimentally proven | (10) |
|  | *Oxalobacter formigenes HOxBLS* | Proteobacteria | Predicted | (10) |
|  | *Oxalobacter formigenes OXCC13* | Proteobacteria | Predicted | (10) |
|  | *Edwardsiella tarda ATCC 23685* | Proteobacteria | Predicted | (10) |
|  | *Proteus penneri ATCC 35198* | Proteobacteria | Predicted | (10) |
|  | *Acinetobacter junii SH205* | Proteobacteria | Predicted | (10) |
|  | *Citrobacter sp. 30_2* | Proteobacteria | Predicted | (10) |
|  | *Citrobacter youngae ATCC 29220* | Proteobacteria | Predicted | (10) |
|  | *Enterobacter cancerogenus ATCC 35316* | Proteobacteria | Predicted | (10) |
|  | *Enterobacteriaceae bacterium 9_2_54FAA* | Proteobacteria | Predicted | (10) |
|  | *Escherichia coli O157:H7 str. Sakai* | Proteobacteria | Predicted | (10) |
|  | *Escherichia coli SE11* | Proteobacteria | Predicted | (10) |
|  | *Escherichia coli str. K-12 substr. MG1655* | Proteobacteria | Predicted and experimentally proven | (10) |
|  | *Escherichia coli UTI89* | Proteobacteria | Predicted | (10) |
|  | *Escherichia sp. 3_2_53FAA* | Proteobacteria | Predicted | (10) |
|  | *Escherichia sp. 4_1_40B* | Proteobacteria | Predicted | (10) |
|  | *Klebsiella pneumoniae 1162281* | Proteobacteria | Predicted and experimentally proven | (10) |
|  | *Klebsiella sp. 1_1_55* | Proteobacteria | Predicted | (10) |
|  | *Providencia alcalifaciens DSM 30120* | Proteobacteria | Predicted | (10) |
|  | *Providencia rettgeri DSM 1131* | Proteobacteria | Predicted | (10) |
|  | *Providencia rustigianii DSM 4541* | Proteobacteria | Predicted | (10) |
|  | *Providencia stuartii ATCC 25827* | Proteobacteria | Predicted | (10) |
|  | *Ralstonia sp. 5_7_47FAA* | Proteobacteria | Predicted | (10) |
|  | *Salmonella enterica subsp. enterica serovar Typhimurium str.* | Proteobacteria | Predicted and experimentally proven | (10) |
|  | *Parasutterella excrementihominis* | Proteobacteria | Predicted | (13) |
|  | *Turicimonas muris* | Proteobacteria | Predicted | (13) |
|  | *Rhodospirillum rubrum* | Proteobacteria | Predicted | (13) |
|  | *Pararhodospirillum photometricum* | Proteobacteria | Predicted | (13) |
|  | *Desulfovibrio piger* | Proteobacteria | Predicted | (13) |
|  | *Escherichia coli Nissle 1917* | Proteobacteria | Experimentally proven | (71)⁠ |
|  | *Escherichia fergusonii* | Proteobacteria | Predicted | (13) |
|  | *Akkermansia muciniphila* | Verrucomicrobiota | Predicted | (13) |
|  |  |  |  |  |
|  |  |  |  |  |
|  |  |  |  |  |
|  |  |  |  |  |
|  |  |  |  |  |
| **Cobalamin (B12)** | *Bifidobacterium infantis* | Actinobacteria | Experimentally proven | (62) |
|  | *Collinsella aerofaciens ATCC 25986* | Actinobacteria | Predicted | (10) |
|  | *Bifidobacterium animalis* | Actinobacteria | Predicted | (6) |
|  | *Bifidobacterium longum* | Actinobacteria | Predicted | (6) |
|  | *Gordonibacter pamelaeae 7-10-1-b* | Actinobacteria | Predicted | (10) |
|  | *Propionibacterium freudenreichii* | Actinomycetota | Experimentally proven | (6,72,73)⁠ |
|  | *Bacteroides thetaiotaomicron VPI-5482* | Bacteroidetes | Predicted and experimentally proven | (10) |
|  | *Bacteroides cellulosilyticus* | Bacteroidetes | Predicted | (13) |
|  | *Bacteroides cellulosilyticus DSM 14838* | Bacteroidetes | Predicted | (10) |
|  | *Bacteroides dorei DSM 17855* | Bacteroidetes | Predicted | (10) |
|  | *Bacteroides fragilis* | Bacteroidetes | Predicted | (13) |
|  | *Bacteroides fragilis 3_1_12* | Bacteroidetes | Predicted | (10) |
|  | *Bacteroides fragilis YCH46* | Bacteroidetes | Predicted | (10) |
|  | *Bacteroides intestinalis DSM 17393* | Bacteroidetes | Predicted | (10) |
|  | *Bacteroides sp. 2_1_16* | Bacteroidetes | Predicted | (10) |
|  | *Bacteroides sp. 2_1_33B* | Bacteroidetes | Predicted | (10) |
|  | *Bacteroides sp. 2_1_7* | Bacteroidetes | Predicted | (10) |
|  | *Bacteroides sp. 3_1_19* | Bacteroidetes | Predicted | (10) |
|  | *Bacteroides sp. 3_1_33FAA* | Bacteroidetes | Predicted | (10) |
|  | *Bacteroides sp. 3_1_40A* | Bacteroidetes | Predicted | (10) |
|  | *Bacteroides sp. 3_2_5* | Bacteroidetes | Predicted | (10) |
|  | *Bacteroides sp. 4_1_36* | Bacteroidetes | Predicted | (10) |
|  | *Bacteroides sp. 4_3_47FAA* | Bacteroidetes | Predicted | (10) |
|  | *Bacteroides sp. 9_1_42FAA* | Bacteroidetes | Predicted | (10) |
|  | *Bacteroides sp. D2* | Bacteroidetes | Predicted | (10) |
|  | *Bacteroides sp. D20* | Bacteroidetes | Predicted | (10) |
|  | *Bacteroides stercoris ATCC 43183* | Bacteroidetes | Predicted | (10) |
|  | *Bacteroides uniformis* | Bacteroidetes | Predicted | (13) |
|  | *Bacteroides uniformis ATCC 8492* | Bacteroidetes | Predicted | (10) |
|  | *Bacteroides vulgatus* | Bacteroidetes | Predicted | (13) |
|  | *Bacteroides vulgatus PC510* | Bacteroidetes | Predicted | (10) |
|  | *Parabacteroides goldsteinii* | Bacteroidetes | Predicted | (13) |
|  | *Parabacteroides distasonis* | Bacteroidetes | Predicted | (10,13) |
|  | *Parabacteroides johnsonii DSM 18315* | Bacteroidetes | Predicted | (10) |
|  | *Parabacteroides merdae* | Bacteroidetes | Predicted | (13) |
|  | *Parabacteroides merdae ATCC 43184* | Bacteroidetes | Predicted | (10) |
|  | *Parabacteroides sp. D13* | Bacteroidetes | Predicted | (10) |
|  | *Bacteroides cellulosilyticus* | Bacteroidetes | Predicted | (13) |
|  | *Bacteroides dorei* | Bacteroidetes | Predicted | (13) |
|  | *Bacteroides fragilis* | Bacteroidetes | Predicted | (13) |
|  | *Bacteroides oleiciplenus* | Bacteroidetes | Predicted | (13) |
|  | *Bacteroides stercoris* | Bacteroidetes | Predicted | (13) |
|  | *Bacteroides uniformis* | Bacteroidetes | Predicted | (13) |
|  | *Bacteroides vulgatus* | Bacteroidetes | Predicted | (13) |
|  | *Butyricimonas virosa* | Bacteroidetes | Predicted | (13) |
|  | *Odoribacter splanchnicus* | Bacteroidetes | Predicted | (13) |
|  | *Parabacteroides distasonis* | Bacteroidetes | Predicted | (13) |
|  | *Parabacteroides goldsteinii* | Bacteroidetes | Predicted | (13) |
|  | *Parabacteroides gordonii* | Bacteroidetes | Predicted | (13) |
|  | *Parabacteroides merdae* | Bacteroidetes | Predicted | (13) |
|  | *Anaerostipes caccae DSM 14662* | Firmicutes | Predicted | (10) |
|  | *Anaerostipes sp. 3_2_56FAA* | Firmicutes | Predicted | (10) |
|  | *Anaerotruncus colihominis DSM 17241* | Firmicutes | Predicted | (10) |
|  | *Bacteroides capillosus ATCC 29799* | Firmicutes | Predicted | (10) |
|  | *Bacteroides pectinophilus ATCC 43243* | Firmicutes | Predicted | (10) |
|  | *Blautia hansenii DSM 20583* | Firmicutes | Predicted | (10) |
|  | *Blautia hydrogenotrophica DSM 10507* | Firmicutes | Predicted | (10) |
|  | *Bryantella formatexigens DSM 14469* | Firmicutes | Predicted | (10) |
|  | *Butyrivibrio fibrisolvens 16/4* | Firmicutes | Predicted | (10) |
|  | *Clostridiales bacterium 1_7_47FAA* | Firmicutes | Predicted | (10) |
|  | *Clostridium asparagiforme DSM 15981* | Firmicutes | Predicted | (10) |
|  | *Clostridium bartlettii DSM 16795* | Firmicutes | Predicted | (10) |
|  | *Clostridium bolteae ATCC BAA-613* | Firmicutes | Predicted | (10) |
|  | *Clostridium clostridioforme 2_1_49FAA* | Firmicutes | Predicted | (10) |
|  | *Clostridium difficile CD196* | Firmicutes | Predicted and experimentally proven | (10) |
|  | *Clostridium difficile NAP07* | Firmicutes | Predicted and experimentally proven | (10) |
|  | *Clostridium difficile NAP08* | Firmicutes | Predicted and experimentally proven | (10) |
|  | *Clostridium hiranonis DSM 13275* | Firmicutes | Predicted | (10) |
|  | *Clostridium hylemonae DSM 15053* | Firmicutes | Predicted | (10) |
|  | *Clostridium scindens ATCC 35704* | Firmicutes | Predicted | (10) |
|  | *Clostridium sp. M62/1* | Firmicutes | Predicted | (10) |
|  | *Clostridium sporogenes ATCC 15579* | Firmicutes | Predicted | (10) |
|  | *Clostridium symbiosum WAL-14163* | Firmicutes | Predicted | (10) |
|  | *Clostridium symbiosum WAL-14673* | Firmicutes | Predicted | (10) |
|  | *Coprococcus comes ATCC 27758* | Firmicutes | Predicted | (10) |
|  | *Dorea longicatena DSM 13814* | Firmicutes | Predicted | (10) |
|  | *Eubacterium hallii DSM 3353* | Firmicutes | Predicted | (10) |
|  | *Eubacterium rectale DSM 17629* | Firmicutes | Predicted | (10) |
|  | *Eubacterium rectale M104/1* | Firmicutes | Predicted | (10) |
|  | *Lachnospiraceae bacterium 8_1_57FAA* | Firmicutes | Predicted | (10) |
|  | *Lactobacillus plantarum* | Firmicutes | Experimentally proven | (6,63)⁠ |
|  | *Lactobacillus coryniformis* | Firmicutes | Experimentally proven | (6,63) |
|  | *Lactobacillus reuteri* | Firmicutes | Experimentally proven | (74,75)⁠ |
|  | *Lactobacillus reuteri CRL1098* | Firmicutes | Experimentally proven | (5,76)⁠ |
|  | *Lactobacillus reuteri CF48-3A* | Firmicutes | Predicted | (10) |
|  | *Lactobacillus reuteri DSM 20016* | Firmicutes | Predicted | (10) |
|  | *Lactobacillus reuteri JCM 1112* | Firmicutes | Predicted | (10) |
|  | *Lactobacillus reuteri MM2-3* | Firmicutes | Predicted | (10) |
|  | *Lactobacillus reuteri MM4-1A* | Firmicutes | Predicted | (10) |
|  | *Lactobacillus reuteri SD2112* | Firmicutes | Predicted | (10) |
|  | *Listeria monocytogenes str. 1/2a F6854* | Firmicutes | Predicted and experimentally proven | (10) |
|  | *Megamonas hypermegale ART12/1* | Firmicutes | Predicted | (10) |
|  | *Mitsuokella multacida DSM 20544* | Firmicutes | Predicted | (10) |
|  | *Phascolarctobacterium sp. YIT 12067* | Firmicutes | Predicted | (10) |
|  | *Roseburia inulinivorans DSM 16841* | Firmicutes | Predicted | (10) |
|  | *Ruminococcaceae bacterium D16* | Firmicutes | Predicted | (10) |
|  | *Ruminococcus gnavus ATCC 29149* | Firmicutes | Predicted | (10) |
|  | *Ruminococcus lactaris ATCC 29176* | Firmicutes | Predicted | (10) |
|  | *Ruminococcus obeum A2-162* | Firmicutes | Predicted | (10) |
|  | *Ruminococcus sp. 5_1_39BFAA* | Firmicutes | Predicted | (10) |
|  | *Ruminococcus sp. SR1/5* | Firmicutes | Predicted | (10) |
|  | *Ruminococcus torques ATCC 27756* | Firmicutes | Predicted | (10) |
|  | *Ruminococcus torques L2-14* | Firmicutes | Predicted | (10) |
|  | *Subdoligranulum variabile DSM 15176* | Firmicutes | Predicted | (10) |
|  | *Veillonella sp. 3_1_44* | Firmicutes | Predicted | (10) |
|  | *Veillonella sp. 6_1_27* | Firmicutes | Predicted | (10) |
|  | *Pseudoflavonifractor capillosus* | Firmicutes | Predicted | (13) |
|  | *[Clostridium] citroniae* | Firmicutes | Predicted | (13) |
|  | *[Clostridium] asparagiforme* | Firmicutes | Predicted | (13) |
|  | *[Clostridium] lavalense* | Firmicutes | Predicted | (13) |
|  | *Agathobaculum desmolans* | Firmicutes | Predicted | (13) |
|  | *Anaerofilum pentosovorans* | Firmicutes | Predicted | (13) |
|  | *Anaerotruncus colihominis* | Firmicutes | Predicted | (13) |
|  | *Anaerotruncus rubiinfantis* | Firmicutes | Predicted | (13) |
|  | *Flavonifractor plautii* | Firmicutes | Predicted | (13) |
|  | *Flintibacter butyricus* | Firmicutes | Predicted | (13) |
|  | *Intestinimonas butyriciproducens* | Firmicutes | Predicted | (13) |
|  | *Phocea massiliensis* | Firmicutes | Predicted | (13) |
|  | *Faecalibacterium prausnitzii* | Firmicutes | Predicted | (13) |
|  | *[Clostridium] celerecrescens* | Firmicutes | Predicted | (13) |
|  | *Intestinibacillus massiliensis* | Firmicutes | Predicted | (13) |
|  | *[Clostridium] amygdalinum* | Firmicutes | Predicted | (13) |
|  | *[Clostridium] hylemonae* | Firmicutes | Predicted | (13) |
|  | *Fusicatenibacter saccharivorans* | Firmicutes | Predicted | (13) |
|  | *Sellimonas intestinalis* | Firmicutes | Predicted | (13) |
|  | *Pseudobutyrivibrio ruminis* | Firmicutes | Predicted | (13) |
|  | *[Eubacterium] eligens* | Firmicutes | Predicted | (13) |
|  | *[Eubacterium] rectale* | Firmicutes | Predicted | (13) |
|  | *Roseburia faecis* | Firmicutes | Predicted | (13) |
|  | *Hungatella hathewayi* | Firmicutes | Predicted | (13) |
|  | *Butyricicoccus pullicaecorum* | Firmicutes | Predicted | (13) |
|  | *[Clostridium] saccharolyticum* | Firmicutes | Predicted | (13) |
|  | *[Clostridium] symbiosum* | Firmicutes | Predicted | (13) |
|  | *[Clostridium] clostridioforme* | Firmicutes | Predicted | (13) |
|  | *Coprococcus catus* | Firmicutes | Predicted | (13) |
|  | *Eisenbergiella tayi* | Firmicutes | Predicted | (13) |
|  | *Blautia schinkii* | Firmicutes | Predicted | (13) |
|  | *Caecibacter massiliensis* | Firmicutes | Predicted | (13) |
|  | *[Clostridium] glycyrrhizinilyticum* | Firmicutes | Predicted | (13) |
|  | *Bariatricus massiliensis* | Firmicutes | Predicted | (13) |
|  | *[Clostridium] scindens* | Firmicutes | Predicted | (13) |
|  | *Blautia glucerasea* | Firmicutes | Predicted | (13) |
|  | *Ruminococcus faecis* | Firmicutes | Predicted | (13) |
|  | *Faecalicatena contorta* | Firmicutes | Predicted | (13) |
|  | *Blautia luti* | Firmicutes | Predicted | (13) |
|  | *Clostridium perfringens* | Firmicutes | Predicted | (13) |
|  | *Coprococcus comes* | Firmicutes | Predicted | (13) |
|  | *Dorea longicatena* | Firmicutes | Predicted | (13) |
|  | *Eubacterium callanderi* | Firmicutes | Predicted | (13) |
|  | *Eubacterium limosum* | Firmicutes | Predicted | (13) |
|  | *Ruminococcus gauvreauii* | Firmicutes | Predicted | (13) |
|  | *Sporobacter termitidis* | Firmicutes | Predicted | (13) |
|  | *[Eubacterium] hallii* | Firmicutes | Predicted | (13) |
|  | *Eubacterium ramulus* | Firmicutes | Predicted | (13) |
|  | *Blautia obeum* | Firmicutes | Predicted | (13) |
|  | *Blautia wexlerae* | Firmicutes | Predicted | (13) |
|  | *[Ruminococcus] torques* | Firmicutes | Predicted | (13) |
|  | *Fusobacterium varium* | Fusobacteria | Predicted | (6)⁠ |
|  | *Fusobacterium gonidiaformans ATCC 25563* | Fusobacteria | Predicted | (10) |
|  | *Fusobacterium mortiferum ATCC 9817* | Fusobacteria | Predicted | (10) |
|  | *Fusobacterium sp. 1_1_41FAA* | Fusobacteria | Predicted | (10) |
|  | *Fusobacterium sp. 2_1_31* | Fusobacteria | Predicted | (10) |
|  | *Fusobacterium sp. 3_1_27* | Fusobacteria | Predicted | (10) |
|  | *Fusobacterium sp. 3_1_33* | Fusobacteria | Predicted | (10) |
|  | *Fusobacterium sp. 3_1_36A2* | Fusobacteria | Predicted | (10) |
|  | *Fusobacterium sp. 3_1_5R* | Fusobacteria | Predicted | (10) |
|  | *Fusobacterium sp. 4_1_13* | Fusobacteria | Predicted | (10) |
|  | *Fusobacterium sp. 7_1* | Fusobacteria | Predicted | (10) |
|  | *Fusobacterium sp. D11* | Fusobacteria | Predicted | (10) |
|  | *Fusobacterium sp. D12* | Fusobacteria | Predicted | (10) |
|  | *Fusobacterium ulcerans ATCC 49185* | Fusobacteria | Predicted | (10) |
|  | *Fusobacterium varium ATCC 27725* | Fusobacteria | Predicted | (10) |
|  | *Bilophila wadsworthia 3_1_6* | Proteobacteria | Predicted | (10) |
|  | *Citrobacter sp. 30_2* | Proteobacteria | Predicted | (10) |
|  | *Citrobacter youngae ATCC 29220* | Proteobacteria | Predicted | (10) |
|  | *Desulfovibrio piger ATCC 29098* | Proteobacteria | Predicted | (10) |
|  | *Desulfovibrio sp. 3_1_syn3* | Proteobacteria | Predicted | (10) |
|  | *Edwardsiella tarda ATCC 23685* | Proteobacteria | Predicted | (10) |
|  | *Enterobacteriaceae bacterium 9_2_54FAA* | Proteobacteria | Predicted | (10) |
|  | *Klebsiella pneumoniae 1162281* | Proteobacteria | Predicted and experimentally proven | (10) |
|  | *Klebsiella sp. 1_1_55* | Proteobacteria | Predicted | (10) |
|  | *Salmonella enterica subsp. enterica serovar Typhimurium str.* | Proteobacteria | Predicted and experimentally proven | (10) |
|  | *Rhodospirillum rubrum* | Proteobacteria | Predicted | (13) |
|  | *Pararhodospirillum photometricum* | Proteobacteria | Predicted | (13) |
|  | *Desulfovibrio piger* | Proteobacteria | Predicted | (13) |

## Table S4. Metadata of samples from Indian and Chinese cohorts used in this study

| **Sample** | **Age** | **Age-group** | **Gender** | **Nationality** | **Location** | **Diet** |
| --- | --- | --- | --- | --- | --- | --- |
| SRR5898908 | 22 | Young_adult | Female | Indian | Bhopal | Veg |
| SRR5898909 | 24 | Young_adult | Male | Indian | Bhopal | Veg |
| SRR5898910 | 25 | Young_adult | Female | Indian | Bhopal | Veg |
| SRR5898911 | 23 | Young_adult | Female | Indian | Bhopal | Veg |
| SRR5898912 | 21 | Young_adult | Female | Indian | Bhopal | Veg |
| SRR5898913 | 21 | Young_adult | Female | Indian | Bhopal | Veg |
| SRR5898914 | 50 | Middle_aged_adult | Male | Indian | Bhopal | Veg |
| SRR5898915 | 27 | Young_adult | Female | Indian | Bhopal | Veg |
| SRR5898916 | 21 | Young_adult | Female | Indian | Bhopal | Veg |
| SRR5898917 | 28 | Young_adult | Female | Indian | Bhopal | Veg |
| SRR5898918 | 30 | Young_adult | Male | Indian | Kasaragod | Non_veg |
| SRR5898919 | 24 | Young_adult | Male | Indian | Kasaragod | Non_veg |
| SRR5898920 | 29 | Young_adult | Female | Indian | Bhopal | Veg |
| SRR5898921 | 24 | Young_adult | Female | Indian | Bhopal | Veg |
| SRR5898922 | 23 | Young_adult | Female | Indian | Bhopal | Veg |
| SRR5898923 | 26 | Young_adult | Female | Indian | Bhopal | Veg |
| SRR5898924 | 26 | Young_adult | Female | Indian | Bhopal | Veg |
| SRR5898925 | 25 | Young_adult | Male | Indian | Bhopal | Veg |
| SRR5898926 | 28 | Young_adult | Male | Indian | Bhopal | Veg |
| SRR5898927 | 26 | Young_adult | Male | Indian | Kasaragod | Non_veg |
| SRR5898928 | 4 | Child | Male | Indian | Kasaragod | Non_veg |
| SRR5898929 | 6 | Child | Male | Indian | Kasaragod | Non_veg |
| SRR5898930 | 9 | Child | Female | Indian | Kasaragod | Non_veg |
| SRR5898931 | 12 | Child | Female | Indian | Kasaragod | Non_veg |
| SRR5898932 | 16 | Child | Male | Indian | Kasaragod | Non_veg |
| SRR5898933 | 10 | Child | Male | Indian | Kasaragod | Non_veg |
| SRR5898934 | 12 | Child | Male | Indian | Kasaragod | Non_veg |
| SRR5898935 | 11 | Child | Female | Indian | Kasaragod | Non_veg |
| SRR5898936 | 7 | Child | Female | Indian | Kasaragod | Non_veg |
| SRR5898937 | 11 | Child | Male | Indian | Kasaragod | Non_veg |
| SRR5898938 | 5 | Child | Male | Indian | Kasaragod | Non_veg |
| SRR5898939 | 3.5 | Child | Male | Indian | Kasaragod | Non_veg |
| SRR5898940 | 6 | Child | Male | Indian | Kasaragod | Non_veg |
| SRR5898941 | 9 | Child | Male | Indian | Kasaragod | Non_veg |
| SRR5898942 | 12 | Child | Male | Indian | Kasaragod | Non_veg |
| SRR5898943 | 10 | Child | Female | Indian | Kasaragod | Non_veg |
| SRR5898944 | 5 | Child | Female | Indian | Kasaragod | Non_veg |
| SRR5898945 | 7 | Child | Female | Indian | Kasaragod | Non_veg |
| SRR5898946 | 26 | Young_adult | Male | Indian | Bhopal | Non_veg |
| SRR5898947 | 27 | Young_adult | Female | Indian | Bhopal | Non_veg |
| SRR5898948 | 58 | Middle_aged_adult | Female | Indian | Kasaragod | Non_veg |
| SRR5898949 | 30 | Young_adult | Female | Indian | Kasaragod | Non_veg |
| SRR5898950 | 50 | Middle_aged_adult | Female | Indian | Kasaragod | Non_veg |
| SRR5898951 | 29 | Young_adult | Male | Indian | Kasaragod | Non_veg |
| SRR5898952 | 30 | Young_adult | Male | Indian | Kasaragod | Non_veg |
| SRR5898953 | 27 | Young_adult | Female | Indian | Kasaragod | Non_veg |
| SRR5898954 | 21 | Young_adult | Male | Indian | Kasaragod | Non_veg |
| SRR5898955 | 58 | Middle_aged_adult | Female | Indian | Kasaragod | Non_veg |
| SRR5898956 | 25 | Young_adult | Male | Indian | Bhopal | Veg |
| SRR5898957 | 53 | Middle_aged_adult | Female | Indian | Kasaragod | Non_veg |
| SRR5898958 | 50 | Middle_aged_adult | Female | Indian | Kasaragod | Non_veg |
| SRR5898959 | 50 | Middle_aged_adult | Female | Indian | Kasaragod | Non_veg |
| SRR5898960 | 23 | Young_adult | Male | Indian | Kasaragod | Non_veg |
| SRR5898961 | 50 | Middle_aged_adult | Female | Indian | Bhopal | Veg |
| SRR5898962 | 53 | Middle_aged_adult | Male | Indian | Kasaragod | Non_veg |
| SRR5898963 | 53 | Middle_aged_adult | Male | Indian | Kasaragod | Non_veg |
| SRR5898964 | 58 | Middle_aged_adult | Male | Indian | Kasaragod | Non_veg |
| SRR5898965 | 56 | Middle_aged_adult | Male | Indian | Kasaragod | Non_veg |
| SRR5898966 | 53 | Middle_aged_adult | Male | Indian | Bhopal | Non_veg |
| SRR5898967 | 60 | Old | Male | Indian | Kasaragod | Non_veg |
| SRR5898968 | 28 | Young_adult | Male | Indian | Bhopal | Non_veg |
| SRR5898969 | 26 | Young_adult | Male | Indian | Bhopal | Veg |
| SRR5898970 | 29 | Young_adult | Male | Indian | Bhopal | Veg |
| SRR5898971 | 30 | Young_adult | Male | Indian | Bhopal | Non_veg |
| SRR5898972 | 23 | Young_adult | Male | Indian | Bhopal | Veg |
| SRR5898973 | 26 | Young_adult | Female | Indian | Bhopal | Veg |
| SRR5898974 | 30 | Young_adult | Male | Indian | Bhopal | Non_veg |
| SRR5898975 | 2.4 | Child | Female | Indian | Bhopal | Veg |
| SRR5898976 | 22 | Young_adult | Male | Indian | Bhopal | Veg |
| SRR5898977 | 30 | Young_adult | Male | Indian | Bhopal | Veg |
| SRR5898978 | 58 | Middle_aged_adult | Male | Indian | Bhopal | Non_veg |
| SRR5898979 | 61 | Old | Male | Indian | Bhopal | Non_veg |
| SRR5898980 | 71 | Old | Male | Indian | Bhopal | Non_veg |
| SRR5898981 | 52 | Middle_aged_adult | Female | Indian | Bhopal | Non_veg |
| SRR5898982 | 62 | Old | Male | Indian | Bhopal | Non_veg |
| SRR5898983 | 52 | Middle_aged_adult | Female | Indian | Bhopal | Non_veg |
| SRR5898984 | 64 | Old | Female | Indian | Bhopal | Non_veg |
| SRR5898985 | 62 | Old | Female | Indian | Bhopal | Non_veg |
| SRR5898986 | 57 | Middle_aged_adult | Male | Indian | Bhopal | Non_veg |
| SRR5898987 | 57 | Middle_aged_adult | Female | Indian | Bhopal | Non_veg |
| SRR5898988 | 29 | Young_adult | Female | Indian | Kasaragod | Non_veg |
| SRR5898989 | 30 | Young_adult | Female | Indian | Kasaragod | Non_veg |
| SRR5898990 | 23 | Young_adult | Male | Indian | Bhopal | Non_veg |
| SRR5898991 | 25 | Young_adult | Male | Indian | Kasaragod | Non_veg |
| SRR5898992 | 23 | Young_adult | Male | Indian | Kasaragod | Non_veg |
| SRR5898993 | 23 | Young_adult | Male | Indian | Kasaragod | Non_veg |
| SRR5898994 | 19 | Young_adult | Male | Indian | Kasaragod | Non_veg |
| SRR5898995 | 23 | Young_adult | Male | Indian | Kasaragod | Non_veg |
| SRR5898996 | 30 | Young_adult | Female | Indian | Kasaragod | Non_veg |
| SRR5898997 | 20 | Young_adult | Male | Indian | Kasaragod | Non_veg |
| SRR5898998 | 29 | Young_adult | Female | Indian | Bhopal | Veg |
| SRR5898999 | 0.6 | Child | Female | Indian | Bhopal | Veg |
| SRR5899000 | 28 | Young_adult | Female | Indian | Bhopal | Veg |
| SRR5899001 | 25 | Young_adult | Female | Indian | Bhopal | Veg |
| SRR5899002 | 0.5 | Child | Female | Indian | Bhopal | Veg |
| SRR5899003 | 0.5 | Child | Female | Indian | Bhopal | Veg |
| SRR5899004 | 24 | Young_adult | Female | Indian | Bhopal | Non_veg |
| SRR5899005 | 30 | Young_adult | Male | Indian | Bhopal | Veg |
| SRR5899006 | 25 | Young_adult | Male | Indian | Bhopal | Veg |
| SRR5899007 | 26 | Young_adult | Male | Indian | Bhopal | Veg |
| SRR5899008 | 55 | Middle_aged_adult | Female | Indian | Kasaragod | Non_veg |
| SRR5899009 | 60 | Old | Female | Indian | Kasaragod | Non_veg |
| SRR5899010 | 27 | Young_adult | Female | Indian | Kasaragod | Non_veg |
| SRR5899011 | 21 | Young_adult | Female | Indian | Kasaragod | Non_veg |
| SRR5899012 | 26 | Young_adult | Female | Indian | Kasaragod | Non_veg |
| SRR5899013 | 28 | Young_adult | Female | Indian | Kasaragod | Non_veg |
| SRR5899014 | 50 | Middle_aged_adult | Female | Indian | Kasaragod | Non_veg |
| SRR5899015 | 55 | Middle_aged_adult | Female | Indian | Kasaragod | Non_veg |
| SRR5899016 | 30 | Young_adult | Female | Indian | Kasaragod | Non_veg |
| SRR5899017 | 23 | Young_adult | Female | Indian | Kasaragod | Non_veg |
| SRR9108942 | 40 | Middle_aged_adult | Male | Indian | Tribal | Non_veg |
| SRR9108943 | 18 | Young_adult | Male | Indian | Tribal | Non_veg |
| SRR9108944 | 32 | Young_adult | Male | Indian | Tribal | Non_veg |
| SRR9108945 | 55 | Middle_aged_adult | Female | Indian | Tribal | Non_veg |
| SRR9108946 | 58 | Middle_aged_adult | Male | Indian | Tribal | Non_veg |
| SRR9108947 | 35 | Young_adult | Male | Indian | Tribal | Non_veg |
| SRR9108948 | 32 | Young_adult | Male | Indian | Tribal | Non_veg |
| SRR9108949 | 24 | Young_adult | Male | Indian | Tribal | Non_veg |
| SRR9108950 | 35 | Young_adult | Male | Indian | Tribal | Veg |
| SRR9108951 | 37 | Middle_aged_adult | Female | Indian | Tribal | Veg |
| SRR9108952 | 22 | Young_adult | Male | Indian | Tribal | Non_veg |
| SRR9108953 | 19 | Young_adult | Male | Indian | Tribal | Non_veg |
| SRR9108954 | 28 | Young_adult | Male | Indian | Tribal | Non_veg |
| SRR9108955 | 27 | Young_adult | Male | Indian | Tribal | Non_veg |
| SRR9108956 | 35 | Young_adult | Male | Indian | Tribal | Veg |
| SRR9108957 | 33 | Young_adult | Male | Indian | Tribal | Non_veg |
| SRR9108958 | 31 | Young_adult | Male | Indian | Tribal | Non_veg |
| SRR9108959 | 22 | Young_adult | Male | Indian | Tribal | Non_veg |
| SRR9108960 | 45 | Middle_aged_adult | Male | Indian | Tribal | Non_veg |
| SRR9108961 | 33 | Young_adult | Male | Indian | Tribal | Non_veg |
| SRR9108962 | 29 | Young_adult | Male | Indian | Tribal | Veg |
| SRR9108963 | 44 | Middle_aged_adult | Male | Indian | Tribal | Non_veg |
| SRR9108964 | 29 | Young_adult | Male | Indian | Tribal | Non_veg |
| SRR9108965 | 31 | Young_adult | Male | Indian | Tribal | Non_veg |
| SRR9108966 | 46 | Middle_aged_adult | Male | Indian | Tribal | Non_veg |
| SRR9108967 | 50 | Middle_aged_adult | Male | Indian | Tribal | Non_veg |
| SRR9108968 | 36 | Middle_aged_adult | Male | Indian | Tribal | Non_veg |
| SRR9108969 | 56 | Middle_aged_adult | Female | Indian | Tribal | Non_veg |
| SRR9108970 | 23 | Young_adult | Female | Indian | Tribal | Non_veg |
| SRR9108971 | 20 | Young_adult | Male | Indian | Tribal | Veg |
| SRR9108972 | 20 | Young_adult | Male | Indian | Tribal | Veg |
| SRR341630 | 23 | Young_adult | Male | Chinese | NA | NA |
| SRR341631 | 23 | Young_adult | Male | Chinese | NA | NA |
| SRR341632 | 23 | Young_adult | Male | Chinese | NA | NA |
| SRR341633 | 38 | Middle_aged_adult | Male | Chinese | NA | NA |
| SRR341634 | 19 | Young_adult | Male | Chinese | NA | NA |
| SRR341635 | 45 | Middle_aged_adult | Female | Chinese | NA | NA |
| SRR341636 | 51 | Middle_aged_adult | Female | Chinese | NA | NA |
| SRR341637 | 33 | Young_adult | Female | Chinese | NA | NA |
| SRR341638 | 35 | Young_adult | Female | Chinese | NA | NA |
| SRR341639 | 32 | Young_adult | Female | Chinese | NA | NA |
| SRR341640 | 26 | Young_adult | Female | Chinese | NA | NA |
| SRR341641 | 30 | Young_adult | Female | Chinese | NA | NA |
| SRR341642 | 41 | Middle_aged_adult | Female | Chinese | NA | NA |
| SRR341643 | 42 | Middle_aged_adult | Female | Chinese | NA | NA |
| SRR341644 | 32 | Young_adult | Male | Chinese | NA | NA |
| SRR341645 | 72 | Old | Male | Chinese | NA | NA |
| SRR341646 | 54 | Middle_aged_adult | Male | Chinese | NA | NA |
| SRR341647 | 32 | Young_adult | Male | Chinese | NA | NA |
| SRR341648 | 42 | Middle_aged_adult | Male | Chinese | NA | NA |
| SRR341649 | 43 | Middle_aged_adult | Male | Chinese | NA | NA |
| SRR341650 | 23 | Young_adult | Male | Chinese | NA | NA |
| SRR341651 | 46 | Middle_aged_adult | Male | Chinese | NA | NA |
| SRR341652 | 52 | Middle_aged_adult | Male | Chinese | NA | NA |
| SRR341699 | 25 | Young_adult | Male | Chinese | NA | NA |
| SRR341700 | 29 | Young_adult | Male | Chinese | NA | NA |
| SRR341701 | 23 | Young_adult | Male | Chinese | NA | NA |
| SRR341702 | 24 | Young_adult | Male | Chinese | NA | NA |
| SRR341703 | 22 | Young_adult | Male | Chinese | NA | NA |
| SRR341704 | 21 | Young_adult | Male | Chinese | NA | NA |
| SRR341705 | 25 | Young_adult | Male | Chinese | NA | NA |
| SRR341706 | 33 | Young_adult | Male | Chinese | NA | NA |
| SRR341707 | 39 | Middle_aged_adult | Male | Chinese | NA | NA |
| SRR341708 | 32 | Young_adult | Female | Chinese | NA | NA |
| SRR341709 | 30 | Young_adult | Female | Chinese | NA | NA |
| SRR341710 | 40 | Middle_aged_adult | Female | Chinese | NA | NA |
| SRR341711 | 38 | Middle_aged_adult | Female | Chinese | NA | NA |
| SRR341712 | 48 | Middle_aged_adult | Male | Chinese | NA | NA |
| SRR341713 | 68 | Old | Male | Chinese | NA | NA |
| SRR341714 | 46 | Middle_aged_adult | Male | Chinese | NA | NA |
| SRR341715 | 33 | Young_adult | Male | Chinese | NA | NA |
| SRR341716 | 30 | Young_adult | Male | Chinese | NA | NA |
| SRR341717 | 41 | Middle_aged_adult | Male | Chinese | NA | NA |
| SRR341718 | 32 | Young_adult | Male | Chinese | NA | NA |
| SRR341719 | 37 | Middle_aged_adult | Male | Chinese | NA | NA |
| SRR341720 | 39 | Middle_aged_adult | Male | Chinese | NA | NA |
| SRR341721 | 26 | Young_adult | Male | Chinese | NA | NA |
| SRR341722 | 24 | Young_adult | Male | Chinese | NA | NA |
| SRR341723 | 28 | Young_adult | Male | Chinese | NA | NA |
| SRR341724 | 38 | Middle_aged_adult | Male | Chinese | NA | NA |
| SRR341725 | 47 | Middle_aged_adult | Male | Chinese | NA | NA |

## Table S5. B-vitamin producers identified in Indian cohorts

| **S.No.** | **Species** | **Phylum** |
| --- | --- | --- |
| 1 | *Adlercreutzia equolifaciens* | Actinomycetota |
| 2 | *Akkermansia muciniphila* | Verrucomicrobiota |
| 3 | *Alistipes finegoldii* | Bacteroidetes |
| 4 | *Alistipes ihumii* | Bacteroidetes |
| 5 | *Alistipes indistinctus* | Bacteroidetes |
| 6 | *Alistipes onderdonkii* | Bacteroidetes |
| 7 | *Alistipes putredinis* | Bacteroidetes |
| 8 | *Alistipes shahii* | Bacteroidetes |
| 9 | *Alistipes timonensis* | Bacteroidetes |
| 10 | *Anaerofustis stercorihominis* | Firmicutes |
| 11 | *Anaerostipes caccae* | Firmicutes |
| 12 | *Anaerostipes hadrus* | Firmicutes |
| 13 | *Anaerotignum lactatifermentans* | Firmicutes |
| 14 | *Anaerotruncus colihominis* | Firmicutes |
| 15 | *Anaerotruncus rubiinfantis* | Firmicutes |
| 16 | *Bacteroides caccae* | Bacteroidetes |
| 17 | *Bacteroides cellulosilyticus* | Bacteroidetes |
| 18 | *Bacteroides eggerthii* | Bacteroidetes |
| 19 | *Bacteroides faecis* | Bacteroidetes |
| 20 | *Bacteroides finegoldii* | Bacteroidetes |
| 21 | *Bacteroides fragilis* | Bacteroidetes |
| 22 | *Bacteroides intestinalis* | Bacteroidetes |
| 23 | *Bacteroides nordii* | Bacteroidetes |
| 24 | *Bacteroides oleiciplenus* | Bacteroidetes |
| 25 | *Bacteroides ovatus* | Bacteroidetes |
| 26 | *Bacteroides pectinophilus* | Firmicutes |
| 27 | *Bacteroides salyersiae* | Bacteroidetes |
| 28 | *Bacteroides stercorirosoris* | Bacteroidetes |
| 29 | *Bacteroides stercoris* | Bacteroidetes |
| 30 | *Bacteroides thetaiotaomicron* | Bacteroidetes |
| 31 | *Bacteroides uniformis* | Bacteroidetes |
| 32 | *Bacteroides xylanisolvens* | Bacteroidetes |
| 33 | *Barnesiella intestinihominis* | Bacteroidetes |
| 34 | *Bifidobacterium adolescentis* | Actinobacteria |
| 35 | *Bifidobacterium angulatum* | Actinobacteria |
| 36 | *Bifidobacterium animalis* | Actinobacteria |
| 37 | *Bifidobacterium bifidum* | Actinobacteria |
| 38 | *Bifidobacterium breve* | Actinobacteria |
| 39 | *Bifidobacterium catenulatum* | Actinobacteria |
| 40 | *Bifidobacterium dentium* | Actinobacteria |
| 41 | *Bifidobacterium longum* | Actinobacteria |
| 42 | *Bifidobacterium pseudocatenulatum* | Actinobacteria |
| 43 | *Bilophila wadsworthia* | Proteobacteria |
| 44 | *Blautia glucerasea* | Firmicutes |
| 45 | *Blautia obeum* | Firmicutes |
| 46 | *Blautia schinkii* | Firmicutes |
| 47 | *Blautia wexlerae* | Firmicutes |
| 48 | *Butyricimonas virosa* | Bacteroidetes |
| 49 | *Butyrivibrio crossotus* | Firmicutes |
| 50 | *Campylobacter upsaliensis* | Proteobacteria |
| 51 | *Christensenella massiliensis* | Firmicutes |
| 52 | *Clostridiales bacterium* | Firmicutes |
| 53 | *Clostridium leptum* | Firmicutes |
| 54 | *Clostridium perfringens* | Firmicutes |
| 55 | *Clostridium scindens* | Firmicutes |
| 56 | *Clostridium symbiosum* | Firmicutes |
| 57 | *Collinsella aerofaciens* | Actinobacteria |
| 58 | *Collinsella intestinalis* | Actinobacteria |
| 59 | *Coprobacter fastidiosus* | Bacteroidetes |
| 60 | *Coprococcus catus* | Firmicutes |
| 61 | *Coprococcus comes* | Firmicutes |
| 62 | *Coprococcus eutactus* | Firmicutes |
| 63 | *Desulfovibrio piger* | Proteobacteria |
| 64 | *Dielma fastidiosa* | Firmicutes |
| 65 | *Dorea longicatena* | Firmicutes |
| 66 | *Eisenbergiella tayi* | Firmicutes |
| 67 | *Enterococcus faecalis* | Firmicutes |
| 68 | *Enterococcus faecium* | Firmicutes |
| 69 | *Enterococcus hirae* | Firmicutes |
| 70 | *Erysipelatoclostridium ramosum* | Firmicutes |
| 71 | *Erysipelotrichaceae bacterium* | Firmicutes |
| 72 | *Escherichia coli* | Proteobacteria |
| 73 | *Eubacterium limosum* | Firmicutes |
| 74 | *Eubacterium ramulus* | Firmicutes |
| 75 | *Eubacterium rectale* | Firmicutes |
| 76 | *Eubacterium siraeum* | Firmicutes |
| 77 | *Eubacterium ventriosum* | Firmicutes |
| 78 | *Faecalibacterium prausnitzii* | Firmicutes |
| 79 | *Faecalicatena contorta* | Firmicutes |
| 80 | *Flavonifractor plautii* | Firmicutes |
| 81 | *Fusicatenibacter saccharivorans* | Firmicutes |
| 82 | *Fusobacterium mortiferum* | Fusobacteria |
| 83 | *Fusobacterium varium* | Fusobacteria |
| 84 | *Gemmiger formicilis* | Firmicutes |
| 85 | *Gordonibacter pamelaeae* | Actinobacteria |
| 86 | *Holdemanella biformis* | Firmicutes |
| 87 | *Hungatella hathewayi* | Firmicutes |
| 88 | *Intestinimonas butyriciproducens* | Firmicutes |
| 89 | *Klebsiella pneumoniae* | Proteobacteria |
| 90 | *Lachnospiraceae bacterium* | Firmicutes |
| 91 | *Lactobacillus acidophilus* | Firmicutes |
| 92 | *Lactobacillus crispatus* | Firmicutes |
| 93 | *Lactobacillus delbrueckii* | Firmicutes |
| 94 | *Lactobacillus helveticus* | Firmicutes |
| 95 | *Lactococcus lactis* | Firmicutes |
| 96 | *Leuconostoc lactis* | Firmicutes |
| 97 | *Mitsuokella multacida* | Firmicutes |
| 98 | *Negativibacillus massiliensis* | Firmicutes |
| 99 | *Odoribacter splanchnicus* | Bacteroidetes |
| 100 | *Parabacteroides distasonis* | Bacteroidetes |
| 101 | *Parabacteroides goldsteinii* | Bacteroidetes |
| 102 | *Parabacteroides gordonii* | Bacteroidetes |
| 103 | *Parabacteroides johnsonii* | Bacteroidetes |
| 104 | *Parabacteroides merdae* | Bacteroidetes |
| 105 | *Paraprevotella clara* | Bacteroidetes |
| 106 | *Parasutterella excrementihominis* | Proteobacteria |
| 107 | *Pediococcus parvulus* | Firmicutes |
| 108 | *Prevotella copri* | Bacteroidetes |
| 109 | *Pseudoflavonifractor capillosus* | Firmicutes |
| 110 | *Romboutsia timonensis* | Firmicutes |
| 111 | *Roseburia faecis* | Firmicutes |
| 112 | *Roseburia intestinalis* | Firmicutes |
| 113 | *Roseburia inulinivorans* | Firmicutes |
| 114 | *Ruminococcaceae bacterium* | Firmicutes |
| 115 | *Ruminococcus bromii* | Firmicutes |
| 116 | *Ruminococcus champanellensis* | Firmicutes |
| 117 | *Ruminococcus gnavus* | Firmicutes |
| 118 | *Ruminococcus lactaris* | Firmicutes |
| 119 | *Ruminococcus torques* | Firmicutes |
| 120 | *Sellimonas intestinalis* | Firmicutes |
| 121 | *Streptococcus equinus* | Firmicutes |
| 122 | *Streptococcus thermophilus* | Firmicutes |
| 123 | *Sutterella wadsworthensis* | Proteobacteria |
| 124 | *Turicibacter sanguinis* | Firmicutes |
| 125 | *Turicimonas muris* | Proteobacteria |
| 126 | *Tyzzerella nexilis* | Firmicutes |
| 127 | *Victivallis vadensis* | Lentisphaerae |

## Table S6. List of species with modest prevalence (≥50%) and mean relative abundance (≥0.1%) in the different dietary groups

|  | **Omnivorous group** | |  | **Vegetarian group** | |
| --- | --- | --- | --- | --- | --- |
| **Species** | **Prevalence (%)** | **Mean relative abundance (%)** | **Species** | **Prevalence (%)** | **Mean relative abundance (%)** |
| *Prevotella copri* | 86.9 | 25.9 | *Prevotella copri* | 90.5 | 34.4 |
| *Faecalibacterium prausnitzii* | 96.0 | 3.2 | *Escherichia coli* | 88.1 | 2.4 |
| *Eubacterium rectale* | 87.9 | 1.7 | *Faecalibacterium prausnitzii* | 95.2 | 1.4 |
| *Bacteroides ovatus* | 73.7 | 1.3 | *Eubacterium rectale* | 85.7 | 1.3 |
| *Bacteroides uniformis* | 72.7 | 1.3 | *Bifidobacterium catenulatum* | 54.7 | 1.1 |
| *Escherichia coli* | 79.8 | 1.2 | *Bifidobacterium adolescentis* | 76.2 | 1.0 |
| *Bifidobacterium adolescentis* | 75.8 | 1.1 | *Roseburia faecis* | 85.7 | 0.9 |
| *Roseburia faecis* | 91.9 | 1.0 | *Bacteroides xylanisolvens* | 52.4 | 0.8 |
| *Alistipes putredinis* | 52.5 | 0.9 | *Ruminococcaceae bacterium* | 59.5 | 0.5 |
| *Ruminococcus bromii* | 62.6 | 0.9 | *Roseburia inulinivorans* | 76.2 | 0.4 |
| *Roseburia inulinivorans* | 89.9 | 0.8 | *Bacteroides ovatus* | 69.0 | 0.3 |
| *Ruminococcaceae bacterium* | 68.7 | 0.8 | *Dorea longicatena* | 69.0 | 0.3 |
| *Bifidobacterium catenulatum* | 57.6 | 0.7 | *Lachnospiraceae bacterium* | 76.2 | 0.3 |
| *Bifidobacterium longum* | 66.7 | 0.5 | *Bifidobacterium longum* | 61.9 | 0.2 |
| *Bacteroides caccae* | 58.6 | 0.5 | *Collinsella aerofaciens* | 71.4 | 0.2 |
| *Bacteroides xylanisolvens* | 56.6 | 0.5 | *Parabacteroides merdae* | 57.1 | 0.2 |
| *Collinsella aerofaciens* | 70.7 | 0.4 | *Parabacteroides distasonis* | 66.7 | 0.2 |
| *Gemmiger formicilis* | 77.8 | 0.4 | *Coprococcus eutactus* | 61.9 | 0.2 |
| *Lachnospiraceae bacterium* | 81.8 | 0.3 | *Klebsiella pneumoniae* | 57.1 | 0.2 |
| *Blautia wexlerae* | 59.6 | 0.3 | *Odoribacter splanchnicus* | 52.4 | 0.1 |
| *Coprococcus eutactus* | 63.6 | 0.3 | *Alistipes shahii* | 54.8 | 0.1 |
| *Dorea longicatena* | 67.7 | 0.2 | *Roseburia intestinalis* | 52.4 | 0.1 |
| *Roseburia intestinalis* | 52.5 | 0.2 | *Gemmiger formicilis* | 64.3 | 0.1 |
| *Parabacteroides distasonis* | 70.7 | 0.2 |  |  |  |
| *Alistipes shahii* | 54.5 | 0.2 |  |  |  |
| *Odoribacter splanchnicus* | 65.7 | 0.2 |  |  |  |
| *Bacteroides thetaiotaomicron* | 58.6 | 0.1 |  |  |  |
| *Ruminococcus torques* | 55.5 | 0.1 |  |  |  |
| *Fusicatenibacter saccharivorans* | 54.5 | 0.1 |  |  |  |

## Table S7. Significantly abundant B-vitamin producers in vegetarian with respect to omnivorous dietary group (FDR p-value <= 0.05)

| **Species** | **baseMean** | **log_2_FoldChange** |
| --- | --- | --- |
| *Campylobacter upsaliensis* | 140.6 | -30.0 |
| *Holdemanella biformis* | 3552.6 | 14.8 |
| *Anaerostipes hadrus* | 4789.8 | 10.0 |
| *Eubacterium ventriosum* | 1120.5 | 12.9 |
| *Parasutterella excrementihominis* | 2484.0 | 14.8 |
| *Coprobacter fastidiosus* | 0.7 | -24.4 |
| *Parabacteroides goldsteinii* | 489.5 | 22.1 |
| *Coprococcus catus* | 2730.9 | 7.4 |
| *Ruminococcus lactaris* | 2026.3 | 11.8 |
| *Blautia glucerasea* | 214.4 | 11.5 |
| *Lactobacillus acidophilus* | 7.8 | -14.4 |
| *Clostridium symbiosum* | 184.3 | 12.3 |
| *Intestinimonas butyriciproducens* | 54.5 | -14.2 |
| *Clostridium leptum* | 3.6 | 13.8 |
| *Bacteroides intestinalis* | 87.8 | 12.9 |
| *Ruminococcus champanellensis* | 726.4 | 12.1 |

## **Table S8. Species with modest prevalence (≥50%) and abundance (≥0.1%) in each of the lifestyle/location groups**

|  | **Urban (Bhopal)** | |  | **Urban (Kasargod)** | |  | **Tribal** | |
| --- | --- | --- | --- | --- | --- | --- | --- | --- |
| **Species** | **Mean relative abundance** | **Prevalence** | **Species** | **Mean relative abundance** | **Prevalence** | **Species** | **Mean relative abundance** | **Prevalence** |
| *Prevotella copri* | 35.0 | 88.7 | *Prevotella copri* | 19.3 | 80.7 | *Prevotella copri* | 33.8 | 100.0 |
| *Escherichia coli* | 2.1 | 86.8 | *Faecalibacterium prausnitzii* | 3.9 | 96.5 | *Faecalibacterium prausnitzii* | 2.9 | 100.0 |
| *Faecalibacterium prausnitzii* | 1.2 | 92.48 | *Bacteroides ovatus* | 2.2 | 82.5 | *Bifidobacterium adolescentis* | 2.6 | 100.0 |
| *Eubacterium rectale* | 1.1 | 79.2 | *Bacteroides uniformis* | 1.8 | 80.7 | *Bifidobacterium catenulatum* | 2.1 | 93.5 |
| *Bacteroides uniformis* | 0.8 | 54.7 | *Eubacterium rectale* | 1.8 | 87.7 | *Eubacterium rectale* | 2.0 | 100.0 |
| *Bifidobacterium catenulatum* | 0.7 | 50.9 | *Escherichia coli* | 1.68 | 77.2 | *Roseburia faecis* | 1.7 | 100.0 |
| *Klebsiella pneumoniae* | 0.7 | 66.0 | *Ruminococcus bromii* | 1.48 | 70.2 | *Bifidobacterium angulatum* | 1.4 | 83.9 |
| *Ruminococcaceae bacterium* | 0.7 | 60.4 | *Alistipes putredinis* | 1.3 | 52.6 | *Collinsella aerofaciens* | 0.8 | 100.0 |
| *Roseburia faecis* | 0.6 | 81.1 | *Roseburia inulinivorans* | 1.2 | 86.0 | *Bifidobacterium longum* | 0.8 | 96.8 |
| *Bifidobacterium adolescentis* | 0.5 | 73.6 | *Bacteroides fragilis* | 1.2 | 61.4 | *Lachnospiraceae bacterium* | 0.7 | 100.0 |
| *Bacteroides caccae* | 0.4 | 50.9 | *Roseburia faecis* | 1.0 | 93.0 | *Blautia wexlerae* | 0.7 | 100.0 |
| *Roseburia inulinivorans* | 0.3 | 77.4 | *Ruminococcaceae bacterium* | 0.8 | 63.2 | *Escherichia coli* | 0.6 | 83.9 |
| *Bacteroides ovatus* | 0.3 | 66.0 | *Bacteroides caccae* | 0.8 | 70.2 | *Dorea longicatena* | 0.5 | 100.0 |
| *Parabacteroides distasonis* | 0.2 | 66.0 | *Bacteroides xylanisolvens* | 0.8 | 56.1 | *Ruminococcaceae bacterium* | 0.4 | 80.6 |
| *Parabacteroides merdae* | 0.2 | 56.6 | *Bifidobacterium adolescentis* | 0.8 | 64.9 | *Coprococcus eutactus* | 0.4 | 83.9 |
| *Dorea longicatena* | 0.2 | 52.8 | *Bifidobacterium longum* | 0.6 | 57.9 | *Ruminococcus torques* | 0.3 | 100.0 |
| *Alistipes shahii* | 0.2 | 54.7 | *Gemmiger formicilis* | 0.5 | 75.4 | *Fusicatenibacter saccharivorans* | 0.3 | 100.0 |
| *Bifidobacterium longum* | 0.1 | 54.7 | *Coprococcus eutactus* | 0.3 | 64.9 | *Ruminococcus bromii* | 0.3 | 67.7 |
| *Odoribacter splanchnicus* | 0.1 | 58.5 | *Collinsella aerofaciens* | 0.3 | 59.6 | *Gemmiger formicilis* | 0.3 | 100.0 |
| *Lachnospiraceae bacterium* | 0.1 | 71.7 | *Lachnospiraceae bacterium* | 0.3 | 77.2 | *Alistipes putredinis* | 0.3 | 58.1 |
| *Collinsella aerofaciens* | 0.1 | 66.0 | *Blautia wexlerae* | 0.3 | 56.1 | *Roseburia intestinalis* | 0.2 | 80.1 |
| *Gemmiger formicilis* | 0.1 | 56.6 | *Bacteroides thetaiotaomicron* | 0.3 | 71.9 | *Roseburia inulinivorans* | 0.2 | 100.0 |
|  |  |  | *Odoribacter splanchnicus* | 0.2 | 68.4 | *Anaerostipes hadrus* | 0.2 | 100.0 |
|  |  |  | *Parabacteroides distasonis* | 0.2 | 73.7 | *Sutterella wadsworthensis* | 0.1 | 80.6 |
|  |  |  | *Alistipes shahii* | 0.2 | 52.6 | *Coprococcus catus* | 0.1 | 100.0 |
|  |  |  | *Dorea longicatena* | 0.1 | 64.9 | *Holdemanella biformis* | 0.1 | 71.0 |
|  |  |  |  |  |  | *Coprococcus comes* | 0.1 | 90.3 |
|  |  |  |  |  |  | *Eubacterium siraeum* | 0.1 | 54.8 |
|  |  |  |  |  |  | *Parabacteroides distasonis* | 0.1 | 67.7 |
|  |  |  |  |  |  | *Blautia schinkii* | 0.1 | 67.7 |
|  |  |  |  |  |  | *Blautia obeum* | 0.1 | 100.0 |
|  |  |  |  |  |  | *Bacteroides uniformis* | 0.1 | 54.8 |

##

## Table S9. Significantly prevalent species between any two lifestyle/location groups (p-adjusted <=0.05).

| **Species** | **Prevalence** | |
| --- | --- | --- |
|  | **Tribal** | **Urban (Bhopal)** |
| *Bifidobacterium adolescentis* | 100.0 | 73.6 |
| *Collinsella aerofaciens* | 100.0 | 66.0 |
| *Bifidobacterium longum* | 96.8 | 54.7 |
| *Bifidobacterium catenulatum* | 93.5 | 50.9 |
| *Bifidobacterium angulatum* | 83.9 | 17.0 |
| *Anaerostipes hadrus* | 100.0 | 3.8 |
| *Blautia obeum* | 100.0 | 11.3 |
| *Blautia wexlerae* | 100.0 | 30.2 |
| *Coprococcus catus* | 100.0 | 3.8 |
| *Dorea longicatena* | 100.0 | 52.8 |
| *Eubacterium rectale* | 100.0 | 79.2 |
| *Fusicatenibacter saccharivorans* | 100.0 | 15.1 |
| *Gemmiger formicilis* | 100.0 | 56.6 |
| *Lachnospiraceae bacterium* | 100.0 | 71.7 |
| *Roseburia faecis* | 100.0 | 81.1 |
| *Ruminococcus torques* | 100.0 | 34.0 |
| *Roseburia inulinivorans* | 100.0 | 77.4 |
| *Coprococcus comes* | 90.3 | 52.8 |
| *Coprococcus eutactus* | 83.9 | 49.1 |
| *Roseburia intestinalis* | 80.6 | 41.5 |
| *Holdemanella biformis* | 71.0 | 9.4 |
| *Blautia schinkii* | 67.7 | 3.8 |
| *Ruminococcus bromii* | 67.7 | 39.6 |
| *Eubacterium siraeum* | 54.8 | 22.6 |
| *Sutterella wadsworthensis* | 80.6 | 43.4 |
| *Klebsiella pneumoniae* | 16.1 | 66.0 |
|  | **Tribal** | **Urban (Kasargod)** |
| *Bifidobacterium adolescentis* | 100.0 | 64.9 |
| *Collinsella aerofaciens* | 100.0 | 59.6 |
| *Bifidobacterium longum* | 96.8 | 57.9 |
| *Bifidobacterium catenulatum* | 93.5 | 42.1 |
| *Bifidobacterium angulatum* | 83.9 | 1.8 |
| *Prevotella copri* | 100.0 | 80.7 |
| *Bacteroides uniformis* | 54.8 | 80.7 |
| *Bacteroides thetaiotaomicron* | 38.7 | 71.9 |
| *Bacteroides caccae* | 32.3 | 70.2 |
| *Bacteroides fragilis* | 32.3 | 61.4 |
| *Anaerostipes hadrus* | 100.0 | 28.1 |
| *Blautia obeum* | 100.0 | 33.3 |
| *Blautia wexlerae* | 100.0 | 56.1 |
| *Coprococcus catus* | 100.0 | 19.3 |
| *Dorea longicatena* | 100.0 | 64.9 |
| *Fusicatenibacter saccharivorans* | 100.0 | 45.6 |
| *Gemmiger formicilis* | 100.0 | 75.4 |
| *Lachnospiraceae bacterium* | 100.0 | 77.2 |
| *Ruminococcus torques* | 100.0 | 45.6 |
| *Coprococcus comes* | 90.3 | 31.6 |
| *Roseburia intestinalis* | 80.6 | 47.4 |
| *Holdemanella biformis* | 71.0 | 8.8 |
| *Blautia schinkii* | 67.7 | 3.5 |
| *Eubacterium siraeum* | 54.8 | 26.3 |
| *Sutterella wadsworthensis* | 80.6 | 33.3 |
|  | **Urban - Central India (Bhopal)** | **Urban - Southern India (Kasargod)** |
| *Bifidobacterium angulatum* | 17.0 | 1.8 |
| *Bacteroides uniformis* | 54.7 | 80.7 |
| *Bacteroides thetaiotaomicron* | 39.6 | 71.9 |
| *Bacteroides caccae* | 50.9 | 70.2 |
| *Bacteroides fragilis* | 35.8 | 61.4 |
| *Anaerostipes hadrus* | 3.8 | 28.1 |
| *Blautia obeum* | 11.3 | 33.3 |
| *Blautia wexlerae* | 30.2 | 56.1 |
| *Coprococcus catus* | 3.8 | 19.3 |
| *Fusicatenibacter saccharivorans* | 15.1 | 45.6 |
| *Gemmiger formicilis* | 56.6 | 75.4 |
| *Coprococcus comes* | 52.8 | 31.6 |
| *Ruminococcus bromii* | 39.6 | 70.2 |
| *Klebsiella pneumoniae* | 66.0 | 38.6 |

##

## Table S10. Differentially abundant species between any two lifestyle/location groups.

| **Urban (Bhopal) w.r.t. Tribal** | | **Urban (Kasargod) w.r.t. Tribal** | | **Urban (Kasargod) w.r.t. Urban (Bhopal)** | |
| --- | --- | --- | --- | --- | --- |
| **Species** | **Log2 fold change** | **Species** | **Log2 Fold Change** | **Species** | **Log2 Fold Change** |
| *Blautia schinkii* | -20.3 | *Bifidobacterium angulatum* | -12.6 | *Bifidobacterium angulatum* | -8.9 |
| *Anaerostipes hadrus* | -15.3 | *Blautia schinkii* | -7.8 | *Coprococcus catus* | 8.5 |
| *Coprococcus catus* | -14.7 | *Coprococcus catus* | -6.2 | *Blautia schinkii* | 12.4 |
| *Holdemanella biformis* | -14.7 | *Bifidobacterium catenulatum* | -5.8 | *Anaerostipes hadrus* | 13.1 |
| *Fusicatenibacter saccharivorans* | -5.4 | *Fusicatenibacter saccharivorans* | -4.7 | *Holdemanella biformis* | 14.0 |
| *Blautia wexlerae* | -3.7 | *Ruminococcus torques* | -3.2 |  |  |
| *Bifidobacterium catenulatum* | -3.3 | *Faecalibacterium prausnitzii* | 1.9 |  |  |
| *Lachnospiraceae bacterium* | -2.0 | *Ruminococcaceae bacterium* | 2.9 |  |  |
| *Escherichia coli* | 2.8 | *Bacteroides thetaiotaomicron* | 3.7 |  |  |
| *Bacteroides uniformis* | 3.5 | *Bacteroides uniformis* | 4.0 |  |  |
| *Bacteroides ovatus* | 3.6 | *Bacteroides ovatus* | 4.3 |  |  |
| *Bacteroides caccae* | 5.3 | *Bacteroides caccae* | 5.3 |  |  |
| *Klebsiella pneumoniae* | 9.7 | *Klebsiella pneumoniae* | 6.0 |  |  |

## Table S11. Effect of **lifestyle/location** on the **abundance** of B-vitamin biosynthetic pathways (reference: Tribal).

| **feature** | **metadata** | **value** | **coef** | **stderr** | **N** | **pval** | **qval** |
| --- | --- | --- | --- | --- | --- | --- | --- |
| thiamin | location | Bhopal | -0.4 | 0.09 | 141 | 0 | 0 |
| riboflavin | location | Bhopal | -0.5 | 0.13 | 141 | 0 | 0.01 |
| thiamin | location | Kasaragod | -0.29 | 0.08 | 141 | 0 | 0.01 |
| pantothenate | location | Bhopal | -1.03 | 0.29 | 141 | 0 | 0.01 |
| niacin | location | Kasaragod | -0.34 | 0.12 | 141 | 0 | 0.04 |
| biotin | location | Bhopal | -0.62 | 0.21 | 141 | 0 | 0.04 |
| folate | location | Kasaragod | -0.94 | 0.33 | 141 | 0.01 | 0.04 |
| riboflavin | location | Kasaragod | -0.33 | 0.12 | 141 | 0.01 | 0.06 |
| thiamin | Gender | Male | -0.14 | 0.06 | 141 | 0.02 | 0.13 |
| niacin | Gender | Male | 0.2 | 0.09 | 141 | 0.02 | 0.13 |
| pantothenate | location | Kasaragod | -0.59 | 0.27 | 141 | 0.03 | 0.16 |
| pyridoxine | location | Bhopal | -0.5 | 0.24 | 141 | 0.04 | 0.16 |
| pyridoxine | Gender | Male | -0.34 | 0.16 | 141 | 0.04 | 0.16 |
| cobalamin | location | Bhopal | -0.5 | 0.24 | 141 | 0.04 | 0.16 |
| cobalamin | age | Young_adult | 0.44 | 0.23 | 141 | 0.06 | 0.22 |

**Table S12. Uniprot ID and annotation of the genes that are part of B-vitamin biosynthesis pathways.**

| **Thiamin biosynthesis pathway (THISYN-PWY)** |  |
| --- | --- |
| Uniprot Ids | Function |
| P0A6B7 | Cysteine desulfurase IscS |
| P0AGG0 | Thiamine-monophosphate kinase |
| P30136 | Phosphomethylpyrimidine synthase |
| P30137 | Thiamine-phosphate synthase |
| P30138 | Sulfur carrier protein ThiS adenylyltransferase |
| P30139 | Thiazole synthase |
| P30140 | 2-iminoacetate synthase |
| P77488 | 1-deoxy-D-xylulose-5-phosphate synthase |
| P77718 | tRNA sulfurtransferase |
| P76422 | Hydroxymethylpyrimidine/phosphomethylpyrimidine kinase |
|  |  |
| **Riboflavin biosynthesis pathway (RIBOSYN2-PWY)** |  |
| Uniprot Ids | Function |
| P0A7I7 | GTP cyclohydrolase-2 |
| P0A7J0 | 3,4-dihydroxy-2-butanone 4-phosphate synthase |
| P0ADP0 | 5-amino-6-(5-phospho-D-ribitylamino)uracil phosphatase YigB |
| P0AFU8 | Riboflavin synthase |
| P0AG40 | Bifunctional riboflavin kinase/FMN adenylyltransferase |
| P25539 | Riboflavin biosynthesis protein RibD |
| P61714 | 6,7-dimethyl-8-ribityllumazine synthase |
| P75809 | 5-amino-6-(5-phospho-D-ribitylamino)uracil phosphatase YbjI |
|  |  |
| **Niacin biosynthesis pathway (PYRIDNUCSYN-PWY)** |  |
| Uniprot Ids | Function |
| P10902 | L-aspartate oxidase |
| P11458 | Quinolinate synthase |
| P30011 | Nicotinate-nucleotide pyrophosphorylase [carboxylating] |
| P0A752 | Nicotinate-nucleotide adenylyltransferase |
| P18843 | NH(3)-dependent NAD(+) synthetase |
|  |  |
| **Pantothenate biosynthesis pathway (PANTO-PWY)** |  |
| Uniprot Ids | Function |
| P31057 | 3-methyl-2-oxobutanoate hydroxymethyltransferase |
| P0A9J4 | 2-dehydropantoate 2-reductase |
| P05793 | Ketol-acid reductoisomerase (NADP(+)) |
| P31663 | Pantothenate synthetase |
| P0A6I3 | Pantothenate kinase |
|  |  |
| **Pyridoxine biosynthesis pathway (PYRIDOXSYN-PWY)** |  |
| Uniprot Ids | Function |
| P05459 | Erythronate-4-phosphate dehydrogenase |
| P0A794 | Pyridoxine 5'-phosphate synthase |
| P0A9B6 | D-erythrose-4-phosphate dehydrogenase |
| P0AFI7 | Pyridoxine/pyridoxamine 5'-phosphate oxidase |
| P19624 | 4-hydroxythreonine-4-phosphate dehydrogenase |
| P23721 | Phosphoserine aminotransferase |
| P77488 | 1-deoxy-D-xylulose-5-phosphate synthase |
|  |  |
| **Biotin biosynthesis pathway (BIOTIN-BIOSYNTHESIS-PWY)** |  |
| Uniprot Ids | Function |
| P12999 | Malonyl-[acyl-carrier protein] O-methyltransferase |
| P0AEK2 | 3-oxoacyl-[acyl-carrier-protein] reductase FabG |
| P0A6Q6 | 3-hydroxyacyl-[acyl-carrier-protein] dehydratase FabZ |
| P0AEK4 | Enoyl-[acyl-carrier-protein] reductase [NADH] FabI |
| P0AAI5 | 3-oxoacyl-[acyl-carrier-protein] synthase 2 |
| P0A953 | 3-oxoacyl-[acyl-carrier-protein] synthase 1 |
| P13001 | Pimeloyl-[acyl-carrier protein] methyl ester esterase |
| P12998 | 8-amino-7-oxononanoate synthase |
| P12995 | Adenosylmethionine-8-amino-7-oxononanoate aminotransferase |
| P0A6E9 | ATP-dependent dethiobiotin synthetase BioD 2 |
| P13000 | ATP-dependent dethiobiotin synthetase BioD 1 |
| P12996 | Biotin synthase |
|  |  |
| **Folate biosynthesis pathway (FOLSYN-PWY)** |  |
| Uniprot Ids | Function |
| P0A6T5 | GTP cyclohydrolase 1 |
| P05041 | Aminodeoxychorismate synthase component 1 |
| P00903 | Aminodeoxychorismate synthase component 2 |
| P28305 | Aminodeoxychorismate lyase |
| P0AFC0 | Dihydroneopterin triphosphate diphosphatase |
| P0AC16 | Dihydroneopterin aldolase |
| P26281 | 2-amino-4-hydroxy-6-hydroxymethyldihydropteridine pyrophosphokinase |
| P0AC13 | Dihydropteroate synthase |
| P08192 | Dihydrofolate synthase/folylpolyglutamate synthase |
| P0ABQ4 | Dihydrofolate reductase |
| P0AFS3 | Dihydromonapterin reductase |
|  |  |
| **Cobalamin biosynthesis pathway (PWY-5507)** |  |
| Uniprot Ids | Function |
| Q9XDM4 | L-threonine kinase |
| P97084 | Threonine-phosphate decarboxylase |
| P0CL07 | Glutamate-1-semialdehyde 2,1-aminomutase |
| Q05603 | Nicotinate-nucleotide--dimethylbenzimidazole phosphoribosyltransferase |
| P25924 | Siroheme synthase |
| Q05592 | Sirohydrochlorin cobaltochelatase |
| Q05593 | Cobalt-precorrin-2 C(20)-methyltransferase |
| Q05590 | Probable cobalt-factor III C(17)-methyltransferase |
| P0A2G9 | Cobalt-precorrin-4 C(11)-methyltransferase |
| Q05631 | Cobalt-precorrin-5A hydrolase |
| Q05628 | Cobalt-precorrin-5B C(1)-methyltransferase |
| Q05591 | Cobalt-precorrin-6A reductase |
| Q05632 | Cobalt-precorrin-6B C(15)-methyltransferase (decarboxylating) |
| P0A2H1 | Cobalt-precorrin-7 C(5)-methyltransferase |
| Q05601 | Cobalt-precorrin-8 methylmutase |
| P29946 | Cobyrinate a,c-diamide synthase |
| P31570 | Corrinoid adenosyltransferase CobA |
| Q8ZQX1 | Flavodoxin 1 |
| Q05597 | Cobyric acid synthase |
| Q05600 | Cobalamin biosynthesis protein CbiB |
| Q05599 | Bifunctional adenosylcobalamin biosynthesis protein CobU |
| Q05602 | Adenosylcobinamide-GDP ribazoletransferase |
| P39701 | Adenosylcobalamin/alpha-ribazole phosphatase |

## Table S13. **Taxonomic annotation of MAGs**.

| **Bins** | **Taxa level** | **Taxonomy** |
| --- | --- | --- |
| MAG_100 | Species | k__Bacteria\|p__Firmicutes\|c__Negativicutes\|o__Veillonellales\|f__Veillonellaceae\|g__Veillonella\|s__Veillonella_seminalis\|t__SGB6923 |
| MAG_111 | Family | k__Bacteria\|p__Firmicutes\|c__Clostridia\|o__Clostridiales\|f__Lachnospiraceae\|g__GGB3420\|s__GGB3420_SGB4539\|t__SGB4539 |
| MAG_116 | Other | k__Bacteria\|p__Firmicutes\|c__CFGB2834\|o__OFGB2834\|f__FGB2834\|g__GGB9061\|s__GGB9061_SGB13979\|t__SGB13979 |
| MAG_117 | Other | k__Bacteria\|p__Firmicutes\|c__CFGB3170\|o__OFGB3170\|f__FGB3170\|g__GGB10690\|s__GGB10690_SGB17345\|t__SGB17345 |
| MAG_121 | Species | k__Bacteria\|p__Bacteroidetes\|c__Bacteroidia\|o__Bacteroidales\|f__Tannerellaceae\|g__Parabacteroides\|s__Parabacteroides_merdae\|t__SGB1949 |
| MAG_122 | Other | k__Bacteria\|p__Firmicutes\|c__CFGB1195\|o__OFGB1195\|f__FGB1195\|g__GGB2949\|s__GGB2949_SGB3926\|t__SGB3926 |
| MAG_131 | Family | k__Bacteria\|p__Firmicutes\|c__Clostridia\|o__Clostridiales\|f__Ruminococcaceae\|g__GGB3341\|s__GGB3341_SGB4420\|t__SGB4420 |
| MAG_133 | Species | k__Bacteria\|p__Firmicutes\|c__Clostridia\|o__Clostridiales\|f__Clostridiales_unclassified\|g__Clostridiales_unclassified\|s__Bacteroides_pectinophilus\|t__SGB5068 |
| MAG_140 | Species | k__Bacteria\|p__Actinobacteria\|c__Coriobacteriia\|o__Coriobacteriales\|f__Atopobiaceae\|g__Parolsenella\|s__Parolsenella_catena\|t__SGB14379 |
| MAG_141 | Species | k__Bacteria\|p__Proteobacteria\|c__Betaproteobacteria\|o__Burkholderiales\|f__Sutterellaceae\|g__Sutterella\|s__Sutterella_wadsworthensis\|t__SGB9283 |
| MAG_145 | Other | k__Bacteria\|p__Firmicutes\|c__CFGB3005\|o__OFGB3005\|f__FGB3005\|g__GGB9468\|s__GGB9468_SGB14861\|t__SGB14861 |
| MAG_149 | Other | k__Bacteria\|p__Firmicutes\|c__CFGB2837\|o__OFGB2837\|f__FGB2837\|g__GGB9081\|s__GGB9081_SGB14005\|t__SGB14005 |
| MAG_150 | Family | k__Bacteria\|p__Bacteroidetes\|c__Bacteroidia\|o__Bacteroidales\|f__Prevotellaceae\|g__GGB1246\|s__GGB1246_SGB1667\|t__SGB1667 |
| MAG_153 | Species | k__Bacteria\|p__Firmicutes\|c__Negativicutes\|o__Acidaminococcales\|f__Acidaminococcaceae\|g__Acidaminococcus\|s__Acidaminococcus_intestini\|t__SGB5736 |
| MAG_157 | Family | k__Bacteria\|p__Firmicutes\|c__Clostridia\|o__Clostridiales\|f__Eubacteriaceae\|g__GGB3278\|s__GGB3278_SGB4328\|t__SGB4328 |
| MAG_158 | Species | k__Bacteria\|p__Firmicutes\|c__Bacilli\|o__Lactobacillales\|f__Lactobacillaceae\|g__Lactobacillus\|s__Lactobacillus_delbrueckii\|t__SGB7020 |
| MAG_161 | Species | k__Bacteria\|p__Bacteroidetes\|c__Bacteroidia\|o__Bacteroidales\|f__Bacteroidaceae\|g__Bacteroides\|s__Bacteroides_coprophilus\|t__SGB1888 |
| MAG_164 | Other | k__Bacteria\|p__Firmicutes\|c__CFGB1479\|o__OFGB1479\|f__FGB1479\|g__GGB3751\|s__GGB3751_SGB5099\|t__SGB5099 |
| MAG_175 | Family | k__Bacteria\|p__Firmicutes\|c__Clostridia\|o__Clostridiales\|f__Ruminococcaceae\|g__GGB9608\|s__GGB9608_SGB15041\|t__SGB15041 |
| MAG_176 | Family | k__Bacteria\|p__Firmicutes\|c__Clostridia\|o__Clostridiales\|f__Lachnospiraceae\|g__GGB3607\|s__GGB3607_SGB4871\|t__SGB4871 |
| MAG_178 | Other | k__Bacteria\|p__Bacteroidetes\|c__CFGB529\|o__OFGB529\|f__FGB529\|g__GGB1093\|s__GGB1093_SGB1404\|t__SGB1404 |
| MAG_181 | Other | k__Bacteria\|p__Firmicutes\|c__CFGB1215\|o__OFGB1215\|f__FGB1215\|g__GGB2975\|s__GGB2975_SGB3957\|t__SGB3957 |
| MAG_184 | Other | k__Bacteria\|p__Firmicutes\|c__CFGB1325\|o__OFGB1325\|f__FGB1325\|g__GGB3175\|s__GGB3175_SGB4191\|t__SGB4191 |
| MAG_190 | Other | k__Bacteria\|p__Firmicutes\|c__CFGB1311\|o__OFGB1311\|f__FGB1311\|g__GGB3139\|s__GGB3139_SGB4152\|t__SGB4152 |
| MAG_197 | Other | k__Bacteria\|p__Firmicutes\|c__CFGB2834\|o__OFGB2834\|f__FGB2834\|g__GGB9060\|s__GGB9060_SGB13977\|t__SGB13977 |
| MAG_199 | Family | k__Bacteria\|p__Proteobacteria\|c__Betaproteobacteria\|o__Burkholderiales\|f__Sutterellaceae\|g__GGB6578\|s__GGB6578_SGB9299\|t__SGB9299 |
| MAG_201 | Other | k__Bacteria\|p__Firmicutes\|c__CFGB2105\|o__OFGB2105\|f__FGB2105\|g__GGB5978\|s__GGB5978_SGB8595\|t__SGB8595 |
| MAG_202 | Family | k__Bacteria\|p__Firmicutes\|c__Negativicutes\|o__Selenomonadales\|f__Selenomonadaceae\|g__GGB4979\|s__GGB4979_SGB6973\|t__SGB6973 |
| MAG_203 | Family | k__Bacteria\|p__Bacteroidetes\|c__Bacteroidia\|o__Bacteroidales\|f__Tannerellaceae\|g__GGB1405\|s__GGB1405_SGB1928\|t__SGB1928 |
| MAG_206 | Species | k__Bacteria\|p__Bacteroidetes\|c__Bacteroidia\|o__Bacteroidales\|f__Prevotellaceae\|g__Paraprevotella\|s__Paraprevotella_clara\|t__SGB1798 |
| MAG_21 | Family | k__Bacteria\|p__Actinobacteria\|c__Actinobacteria\|o__Corynebacteriales\|f__Nocardiaceae\|g__GGB38239\|s__GGB38239_SGB47716\|t__SGB47716 |
| MAG_210 | Species | k__Bacteria\|p__Firmicutes\|c__Firmicutes_unclassified\|o__Firmicutes_unclassified\|f__Firmicutes_unclassified\|g__Firmicutes_unclassified\|s__Firmicutes_bacterium_AF16_15\|t__SGB4993 |
| MAG_217 | Family | k__Bacteria\|p__Firmicutes\|c__Clostridia\|o__Clostridiales\|f__Eubacteriaceae\|g__GGB3280\|s__GGB3280_SGB4333\|t__SGB4333 |
| MAG_229 | Other | k__Bacteria\|p__Firmicutes\|c__CFGB3069\|o__OFGB3069\|f__FGB3069\|g__GGB9762\|s__GGB9762_SGB15377\|t__SGB15377 |
| MAG_23 | Species | k__Bacteria\|p__Bacteroidetes\|c__Bacteroidia\|o__Bacteroidales\|f__Bacteroidaceae\|g__Bacteroides\|s__Bacteroides_thetaiotaomicron\|t__SGB1861 |
| MAG_248 | Species | k__Archaea\|p__Euryarchaeota\|c__Methanobacteria\|o__Methanobacteriales\|f__Methanobacteriaceae\|g__Methanobrevibacter\|s__Methanobrevibacter_smithii\|t__SGB714 |
| MAG_251 | Species | k__Bacteria\|p__Firmicutes\|c__Clostridia\|o__Clostridiales\|f__Ruminococcaceae\|g__Ruminococcus\|s__Ruminococcus_sp_JE7A12\|t__SGB4272 |
| MAG_252 | Species | k__Bacteria\|p__Firmicutes\|c__Clostridia\|o__Clostridiales\|f__Ruminococcaceae\|g__Flavonifractor\|s__Flavonifractor_plautii\|t__SGB15132 |
| MAG_264 | Other | k__Bacteria\|p__Firmicutes\|c__CFGB1877\|o__OFGB1877\|f__FGB1877\|g__GGB4978\|s__GGB4978_SGB6970\|t__SGB6970 |
| MAG_275 | Family | k__Bacteria\|p__Firmicutes\|c__Clostridia\|o__Clostridiales\|f__Ruminococcaceae\|g__GGB13533\|s__GGB13533_SGB20850\|t__SGB20850 |
| MAG_279 | Species | k__Bacteria\|p__Firmicutes\|c__Clostridia\|o__Clostridiales\|f__Clostridiaceae\|g__Clostridium\|s__Clostridium_sp_AF15_49\|t__SGB5111 |
| MAG_289 | Family | k__Bacteria\|p__Firmicutes\|c__Clostridia\|o__Clostridiales\|f__Clostridiaceae\|g__GGB3486\|s__GGB3486_SGB4658\|t__SGB4658 |
| MAG_29 | Other | k__Bacteria\|p__Firmicutes\|c__CFGB1778\|o__OFGB1778\|f__FGB1778\|g__GGB4700\|s__GGB4700_SGB6506\|t__SGB6506 |
| MAG_290 | Species | k__Bacteria\|p__Firmicutes\|c__Clostridia\|o__Clostridiales\|f__Ruminococcaceae\|g__Subdoligranulum\|s__Subdoligranulum_sp_APC924_74\|t__SGB15286 |
| MAG_296 | Species | k__Bacteria\|p__Firmicutes\|c__Clostridia\|o__Clostridiales\|f__Lachnospiraceae\|g__Mediterraneibacter\|s__Mediterraneibacter_sp_gm002\|t__SGB4553 |
| MAG_302 | Family | k__Bacteria\|p__Firmicutes\|c__Clostridia\|o__Clostridiales\|f__Lachnospiraceae\|g__GGB3733\|s__GGB3733_SGB5066\|t__SGB5066 |
| MAG_308 | Other | k__Bacteria\|p__Proteobacteria\|c__CFGB1061\|o__OFGB1061\|f__FGB1061\|g__GGB2730\|s__GGB2730_SGB3672\|t__SGB3672 |
| MAG_312 | Other | k__Bacteria\|p__Actinobacteria\|c__CFGB3176\|o__OFGB3176\|f__FGB3176\|g__GGB10708\|s__GGB10708_SGB17364\|t__SGB17364 |
| MAG_316 | Other | k__Bacteria\|p__Firmicutes\|c__CFGB2932\|o__OFGB2932\|f__FGB2932\|g__GGB9258\|s__GGB9258_SGB14205\|t__SGB14205 |
| MAG_318 | Family | k__Bacteria\|p__Firmicutes\|c__Clostridia\|o__Clostridiales\|f__Eubacteriaceae\|g__GGB3746\|s__GGB3746_SGB5089\|t__SGB5089 |
| MAG_325 | Other | k__Bacteria\|p__Firmicutes\|c__CFGB1311\|o__OFGB1311\|f__FGB1311\|g__GGB3141\|s__GGB3141_SGB4154\|t__SGB4154 |
| MAG_327 | Other | k__Bacteria\|p__Bacteroidetes\|c__CFGB630\|o__OFGB630\|f__FGB630\|g__GGB1497\|s__GGB1497_SGB2076\|t__SGB2076 |
| MAG_328 | Other | k__Bacteria\|p__Firmicutes\|c__CFGB2932\|o__OFGB2932\|f__FGB2932\|g__GGB9261\|s__GGB9261_SGB14209\|t__SGB14209 |
| MAG_34 | Species | k__Bacteria\|p__Bacteroidetes\|c__Bacteroidia\|o__Bacteroidales\|f__Rikenellaceae\|g__Alistipes\|s__Alistipes_obesi\|t__SGB2290 |
| MAG_348 | Other | k__Bacteria\|p__Firmicutes\|c__CFGB1787\|o__OFGB1787\|f__FGB1787\|g__GGB4750\|s__GGB4750_SGB6579\|t__SGB6579 |
| MAG_35 | Species | k__Bacteria\|p__Firmicutes\|c__Firmicutes_unclassified\|o__Firmicutes_unclassified\|f__Firmicutes_unclassified\|g__Firmicutes_unclassified\|s__Firmicutes_bacterium_AF36_3BH\|t__SGB4269 |
| MAG_350 | Other | k__Bacteria\|p__Firmicutes\|c__CFGB1778\|o__OFGB1778\|f__FGB1778\|g__GGB4704\|s__GGB4704_SGB6511\|t__SGB6511 |
| MAG_352 | Species | k__Bacteria\|p__Firmicutes\|c__Negativicutes\|o__Acidaminococcales\|f__Acidaminococcaceae\|g__Phascolarctobacterium\|s__Phascolarctobacterium_faecium\|t__SGB5792 |
| MAG_354 | Species | k__Bacteria\|p__Firmicutes\|c__Clostridia\|o__Clostridiales\|f__Clostridiaceae\|g__Clostridium\|s__Clostridium_sp_AF02_29\|t__SGB4705 |
| MAG_356 | Family | k__Bacteria\|p__Bacteroidetes\|c__Bacteroidia\|o__Bacteroidales\|f__Prevotellaceae\|g__GGB1147\|s__GGB1147_SGB1473\|t__SGB1473 |
| MAG_36 | Species | k__Bacteria\|p__Firmicutes\|c__Clostridia\|o__Clostridiales\|f__Lachnospiraceae\|g__Coprococcus\|s__Coprococcus_sp_OM04_5BH\|t__SGB5115 |
| MAG_363 | Family | k__Bacteria\|p__Elusimicrobia\|c__Elusimicrobia\|o__Elusimicrobiales\|f__Elusimicrobiaceae\|g__GGB12695\|s__GGB12695_SGB19692\|t__SGB19692 |
| MAG_374 | Other | k__Bacteria\|p__Bacteroidetes\|c__CFGB619\|o__OFGB619\|f__FGB619\|g__GGB1458\|s__GGB1458_SGB2021\|t__SGB2021 |
| MAG_385 | Species | k__Bacteria\|p__Proteobacteria\|c__Deltaproteobacteria\|o__Desulfovibrionales\|f__Desulfovibrionaceae\|g__Bilophila\|s__Bilophila_wadsworthia\|t__SGB15452 |
| MAG_386 | Family | k__Bacteria\|p__Firmicutes\|c__Clostridia\|o__Clostridiales\|f__Clostridiaceae\|g__GGB3617\|s__GGB3617_SGB4891\|t__SGB4891 |
| MAG_389 | Family | k__Bacteria\|p__Firmicutes\|c__Clostridia\|o__Clostridiales\|f__Ruminococcaceae\|g__GGB3340\|s__GGB3340_SGB4419\|t__SGB4419 |
| MAG_39 | Other | k__Bacteria\|p__Firmicutes\|c__CFGB1311\|o__OFGB1311\|f__FGB1311\|g__GGB3140\|s__GGB3140_SGB4153\|t__SGB4153 |
| MAG_392 | Other | k__Bacteria\|p__Bacteroidetes\|c__CFGB544\|o__OFGB544\|f__FGB544\|g__GGB1123\|s__GGB1123_SGB1437\|t__SGB1437 |
| MAG_399 | Species | k__Bacteria\|p__Firmicutes\|c__Clostridia\|o__Clostridiales\|f__Clostridiaceae\|g__Clostridium\|s__Clostridium_sp_AF36_4\|t__SGB4644 |
| MAG_4 | Species | k__Bacteria\|p__Firmicutes\|c__Bacilli\|o__Lactobacillales\|f__Enterococcaceae\|g__Enterococcus\|s__Enterococcus_faecium\|t__SGB7968 |
| MAG_402 | Other | k__Bacteria\|p__Proteobacteria\|c__CFGB2116\|o__OFGB2116\|f__FGB2116\|g__GGB6037\|s__GGB6037_SGB8671\|t__SGB8671 |
| MAG_404 | Family | k__Bacteria\|p__Firmicutes\|c__Clostridia\|o__Clostridiales\|f__Clostridiaceae\|g__GGB3623\|s__GGB3623_SGB4900\|t__SGB4900 |
| MAG_408 | Species | k__Bacteria\|p__Firmicutes\|c__Clostridia\|o__Clostridiales\|f__Ruminococcaceae\|g__Ruthenibacterium\|s__Ruthenibacterium_lactatiformans\|t__SGB15271 |
| MAG_418 | Species | k__Bacteria\|p__Firmicutes\|c__Clostridia\|o__Clostridiales\|f__Lachnospiraceae\|g__Anaerobutyricum\|s__Anaerobutyricum_hallii\|t__SGB4532 |
| MAG_420 | Family | k__Bacteria\|p__Firmicutes\|c__Clostridia\|o__Clostridiales\|f__Clostridiaceae\|g__GGB9568\|s__GGB9568_SGB14980\|t__SGB14980 |
| MAG_426 | Species | k__Bacteria\|p__Firmicutes\|c__Erysipelotrichia\|o__Erysipelotrichales\|f__Erysipelotrichaceae\|g__Erysipelatoclostridium\|s__Erysipelatoclostridium_ramosum\|t__SGB6744 |
| MAG_430 | Species | k__Bacteria\|p__Firmicutes\|c__Clostridia\|o__Clostridiales\|f__Ruminococcaceae\|g__Ruminococcaceae_unclassified\|s__Eubacterium_siraeum\|t__SGB4198 |
| MAG_438 | Other | k__Bacteria\|p__Firmicutes\|c__CFGB1332\|o__OFGB1332\|f__FGB1332\|g__GGB3215\|s__GGB3215_SGB4247\|t__SGB4247 |
| MAG_439 | Genus | k__Bacteria\|p__Firmicutes\|c__Clostridia\|o__Clostridiales\|f__Eubacteriaceae\|g__Eubacterium\|s__Eubacterium_SGB4329\|t__SGB4329 |
| MAG_440 | Other | k__Bacteria\|p__Proteobacteria\|c__CFGB4212\|o__OFGB4212\|f__FGB4212\|g__GGB12502\|s__GGB12502_SGB19434\|t__SGB19434 |
| MAG_441 | Family | k__Bacteria\|p__Firmicutes\|c__Clostridia\|o__Clostridiales\|f__Eubacteriaceae\|g__GGB3737\|s__GGB3737_SGB5071\|t__SGB5071 |
| MAG_444 | Species | k__Bacteria\|p__Firmicutes\|c__Clostridia\|o__Clostridiales\|f__Clostridiaceae\|g__Clostridium\|s__Clostridium_sp_AM49_4BH\|t__SGB4652 |
| MAG_445 | Species | k__Bacteria\|p__Firmicutes\|c__Clostridia\|o__Clostridiales\|f__Lachnospiraceae\|g__Mediterraneibacter\|s__Ruminococcus_torques\|t__SGB4608 |
| MAG_451 | Species | k__Bacteria\|p__Firmicutes\|c__Clostridia\|o__Clostridiales\|f__Peptostreptococcaceae\|g__Romboutsia\|s__Romboutsia_timonensis\|t__SGB6148 |
| MAG_454 | Species | k__Bacteria\|p__Bacteroidetes\|c__Bacteroidia\|o__Bacteroidales\|f__Tannerellaceae\|g__Parabacteroides\|s__Parabacteroides_distasonis\|t__SGB1934 |
| MAG_456 | Family | k__Bacteria\|p__Firmicutes\|c__Clostridia\|o__Clostridiales\|f__Eubacteriaceae\|g__GGB3744\|s__GGB3744_SGB5087\|t__SGB5087 |
| MAG_46 | Family | k__Bacteria\|p__Elusimicrobia\|c__Elusimicrobia\|o__Elusimicrobiales\|f__Elusimicrobiaceae\|g__GGB12696\|s__GGB12696_SGB19694\|t__SGB19694 |
| MAG_460 | Species | k__Bacteria\|p__Proteobacteria\|c__Deltaproteobacteria\|o__Desulfovibrionales\|f__Desulfovibrionaceae\|g__Desulfovibrio\|s__Desulfovibrio_piger\|t__SGB15467 |
| MAG_461 | Other | k__Bacteria\|p__Firmicutes\|c__CFGB2837\|o__OFGB2837\|f__FGB2837\|g__GGB9082\|s__GGB9082_SGB14007\|t__SGB14007 |
| MAG_463 | Other | k__Bacteria\|p__Proteobacteria\|c__CFGB2163\|o__OFGB2163\|f__FGB2163\|g__GGB6127\|s__GGB6127_SGB8773\|t__SGB8773 |
| MAG_478 | Other | k__Bacteria\|p__Firmicutes\|c__CFGB1218\|o__OFGB1218\|f__FGB1218\|g__GGB2983\|s__GGB2983_SGB3965\|t__SGB3965 |
| MAG_480 | Species | k__Bacteria\|p__Firmicutes\|c__Clostridia\|o__Clostridiales\|f__Lachnospiraceae\|g__Butyrivibrio\|s__Butyrivibrio_crossotus\|t__SGB5065 |
| MAG_487 | Other | k__Bacteria\|p__Actinobacteria\|c__CFGB2992\|o__OFGB2992\|f__FGB2992\|g__GGB9411\|s__GGB9411_SGB14765\|t__SGB14765 |
| MAG_49 | Other | k__Bacteria\|p__Proteobacteria\|c__CFGB2402\|o__OFGB2402\|f__FGB2402\|g__GGB6593\|s__GGB6593_SGB9323\|t__SGB9323 |
| MAG_494 | Species | k__Bacteria\|p__Firmicutes\|c__Clostridia\|o__Clostridiales\|f__Clostridiales_unclassified\|g__Clostridiales_unclassified\|s__Clostridiales_bacterium_KLE1615\|t__SGB5090 |
| MAG_495 | Other | k__Bacteria\|p__Firmicutes\|c__CFGB2873\|o__OFGB2873\|f__FGB2873\|g__GGB9172\|s__GGB9172_SGB14110\|t__SGB14110 |
| MAG_5 | Family | k__Bacteria\|p__Firmicutes\|c__Erysipelotrichia\|o__Erysipelotrichales\|f__Erysipelotrichaceae\|g__GGB4887\|s__GGB4887_SGB6836\|t__SGB6836 |
| MAG_505 | Other | k__Bacteria\|p__Firmicutes\|c__CFGB2984\|o__OFGB2984\|f__FGB2984\|g__GGB9345\|s__GGB9345_SGB14311\|t__SGB14311 |
| MAG_51 | Species | k__Bacteria\|p__Bacteroidetes\|c__Bacteroidia\|o__Bacteroidales\|f__Barnesiellaceae\|g__Barnesiella\|s__Barnesiella_intestinihominis\|t__SGB1965 |
| MAG_516 | Species | k__Bacteria\|p__Bacteroidetes\|c__Bacteroidia\|o__Bacteroidales\|f__Bacteroidaceae\|g__Bacteroides\|s__Bacteroides_coprocola\|t__SGB1891 |
| MAG_522 | Other | k__Bacteria\|p__Firmicutes\|c__CFGB3069\|o__OFGB3069\|f__FGB3069\|g__GGB9767\|s__GGB9767_SGB15385\|t__SGB15385 |
| MAG_533 | Species | k__Bacteria\|p__Proteobacteria\|c__Betaproteobacteria\|o__Burkholderiales\|f__Sutterellaceae\|g__Duodenibacillus\|s__Duodenibacillus_massiliensis\|t__SGB9273 |
| MAG_538 | Species | k__Bacteria\|p__Firmicutes\|c__Clostridia\|o__Clostridiales\|f__Lachnospiraceae\|g__Blautia\|s__Ruminococcus_gnavus\|t__SGB4584 |
| MAG_539 | Species | k__Bacteria\|p__Firmicutes\|c__Clostridia\|o__Clostridiales\|f__Lachnospiraceae\|g__Dorea\|s__Dorea_sp_AF36_15AT\|t__SGB4552 |
| MAG_555 | Species | k__Bacteria\|p__Firmicutes\|c__Clostridia\|o__Clostridiales\|f__Lachnospiraceae\|g__Anaerotignum\|s__Anaerotignum_faecicola\|t__SGB5190 |
| MAG_56 | Other | k__Bacteria\|p__Firmicutes\|c__CFGB1311\|o__OFGB1311\|f__FGB1311\|g__GGB3142\|s__GGB3142_SGB4155\|t__SGB4155 |
| MAG_560 | Species | k__Bacteria\|p__Firmicutes\|c__Clostridia\|o__Clostridiales\|f__Lachnospiraceae\|g__Lachnospiraceae_unclassified\|s__Lachnospiraceae_bacterium_AM48_27BH\|t__SGB4706 |
| MAG_565 | Species | k__Bacteria\|p__Bacteroidetes\|c__Bacteroidia\|o__Bacteroidales\|f__Odoribacteraceae\|g__Odoribacter\|s__Odoribacter_splanchnicus\|t__SGB1790 |
| MAG_567 | Species | k__Bacteria\|p__Bacteroidetes\|c__Bacteroidia\|o__Bacteroidales\|f__Bacteroidaceae\|g__Bacteroides\|s__Bacteroides_fragilis\|t__SGB1855 |
| MAG_57 | Species | k__Bacteria\|p__Firmicutes\|c__Clostridia\|o__Clostridiales\|f__Lachnospiraceae\|g__Blautia\|s__Blautia_sp_OF03_15BH\|t__SGB4779 |
| MAG_572 | Other | k__Bacteria\|p__Firmicutes\|c__CFGB1311\|o__OFGB1311\|f__FGB1311\|g__GGB3140\|s__GGB3140_SGB4153\|t__SGB4153 |
| MAG_58 | Family | k__Bacteria\|p__Firmicutes\|c__Clostridia\|o__Clostridiales\|f__Lachnospiraceae\|g__GGB3646\|s__GGB3646_SGB4953\|t__SGB4953 |
| MAG_581 | Family | k__Bacteria\|p__Firmicutes\|c__Clostridia\|o__Clostridiales\|f__Ruminococcaceae\|g__GGB9737\|s__GGB9737_SGB15310\|t__SGB15310 |
| MAG_583 | Other | k__Bacteria\|p__Firmicutes\|c__CFGB1355\|o__OFGB1355\|f__FGB1355\|g__GGB3305\|s__GGB3305_SGB4368\|t__SGB4368 |
| MAG_587 | Species | k__Bacteria\|p__Firmicutes\|c__Bacilli\|o__Lactobacillales\|f__Lactobacillaceae\|g__Lactobacillus\|s__Lactobacillus_ruminis\|t__SGB7061 |
| MAG_593 | Other | k__Bacteria\|p__Firmicutes\|c__CFGB1418\|o__OFGB1418\|f__FGB1418\|g__GGB3473\|s__GGB3473_SGB4636\|t__SGB4636 |
| MAG_597 | Species | k__Bacteria\|p__Spirochaetes\|c__Spirochaetia\|o__Spirochaetales\|f__Spirochaetaceae\|g__Treponema\|s__Treponema_succinifaciens\|t__SGB3546 |
| MAG_6 | Other | k__Bacteria\|p__Firmicutes\|c__CFGB343\|o__OFGB343\|f__FGB343\|g__GGB781\|s__GGB781_SGB1024\|t__SGB1024 |
| MAG_60 | Species | k__Bacteria\|p__Firmicutes\|c__Clostridia\|o__Clostridiales\|f__Lachnospiraceae\|g__Lachnospiraceae_unclassified\|s__Lachnospiraceae_bacterium_OM04_12BH\|t__SGB4893 |
| MAG_601 | Family | k__Bacteria\|p__Firmicutes\|c__Clostridia\|o__Clostridiales\|f__Clostridiaceae\|g__GGB3612\|s__GGB3612_SGB4881\|t__SGB4881 |
| MAG_604 | Other | k__Bacteria\|p__Firmicutes\|c__CFGB2849\|o__OFGB2849\|f__FGB2849\|g__GGB9120\|s__GGB9120_SGB14053\|t__SGB14053 |
| MAG_61 | Species | k__Bacteria\|p__Actinobacteria\|c__Actinobacteria\|o__Bifidobacteriales\|f__Bifidobacteriaceae\|g__Bifidobacterium\|s__Bifidobacterium_bifidum\|t__SGB17256 |
| MAG_613 | Other | k__Bacteria\|p__Firmicutes\|c__CFGB2984\|o__OFGB2984\|f__FGB2984\|g__GGB9347\|s__GGB9347_SGB14313\|t__SGB14313 |
| MAG_615 | Species | k__Bacteria\|p__Firmicutes\|c__Clostridia\|o__Clostridiales\|f__Ruminococcaceae\|g__Ruminococcaceae_unclassified\|s__Ruminococcaceae_bacterium\|t__SGB4391 |
| MAG_620 | Family | k__Bacteria\|p__Firmicutes\|c__Clostridia\|o__Clostridiales\|f__Lachnospiraceae\|g__GGB3588\|s__GGB3588_SGB4808\|t__SGB4808 |
| MAG_631 | Other | k__Bacteria\|p__Firmicutes\|c__CFGB3068\|o__OFGB3068\|f__FGB3068\|g__GGB9760\|s__GGB9760_SGB15373\|t__SGB15373 |
| MAG_632 | Species | k__Bacteria\|p__Bacteroidetes\|c__Bacteroidia\|o__Bacteroidales\|f__Bacteroidaceae\|g__Bacteroides\|s__Bacteroides_fragilis\|t__SGB1853 |
| MAG_636 | Other | k__Bacteria\|p__Bacteroidetes\|c__CFGB654\|o__OFGB654\|f__FGB654\|g__GGB1617\|s__GGB1617_SGB2214\|t__SGB2214 |
| MAG_638 | Species | k__Bacteria\|p__Firmicutes\|c__Clostridia\|o__Clostridiales\|f__Lachnospiraceae\|g__Roseburia\|s__Roseburia_sp_AF02_12\|t__SGB4938 |
| MAG_64 | Species | k__Bacteria\|p__Proteobacteria\|c__Betaproteobacteria\|o__Burkholderiales\|f__Sutterellaceae\|g__Parasutterella\|s__Parasutterella_excrementihominis\|t__SGB9262 |
| MAG_640 | Species | k__Bacteria\|p__Firmicutes\|c__Bacilli\|o__Lactobacillales\|f__Streptococcaceae\|g__Streptococcus\|s__Streptococcus_equinus\|t__SGB8022 |
| MAG_658 | Other | k__Bacteria\|p__Firmicutes\|c__CFGB1776\|o__OFGB1776\|f__FGB1776\|g__GGB4669\|s__GGB4669_SGB6458\|t__SGB6458 |
| MAG_668 | Other | k__Bacteria\|p__Firmicutes\|c__CFGB2886\|o__OFGB2886\|f__FGB2886\|g__GGB9192\|s__GGB9192_SGB14131\|t__SGB14131 |
| MAG_669 | Family | k__Bacteria\|p__Bacteroidetes\|c__Bacteroidia\|o__Bacteroidales\|f__Prevotellaceae\|g__GGB1267\|s__GGB1267_SGB1701\|t__SGB1701 |
| MAG_671 | Other | k__Bacteria\|p__Firmicutes\|c__CFGB2837\|o__OFGB2837\|f__FGB2837\|g__GGB9083\|s__GGB9083_SGB14011\|t__SGB14011 |
| MAG_676 | Other | k__Bacteria\|p__Proteobacteria\|c__CFGB1062\|o__OFGB1062\|f__FGB1062\|g__GGB2734\|s__GGB2734_SGB3677\|t__SGB3677 |
| MAG_677 | Family | k__Bacteria\|p__Proteobacteria\|c__Betaproteobacteria\|o__Burkholderiales\|f__Sutterellaceae\|g__GGB6565\|s__GGB6565_SGB9274\|t__SGB9274 |
| MAG_683 | Species | k__Bacteria\|p__Firmicutes\|c__Clostridia\|o__Clostridiales\|f__Clostridiaceae\|g__Clostridium\|s__Clostridium_sp_AF34_13\|t__SGB4659 |
| MAG_684 | Species | k__Bacteria\|p__Firmicutes\|c__Clostridia\|o__Clostridiales\|f__Ruminococcaceae\|g__Ruminococcus\|s__Ruminococcus_callidus\|t__SGB4422 |
| MAG_685 | Species | k__Bacteria\|p__Bacteroidetes\|c__Bacteroidia\|o__Bacteroidales\|f__Rikenellaceae\|g__Alistipes\|s__Alistipes_indistinctus\|t__SGB2325 |
| MAG_692 | Species | k__Bacteria\|p__Proteobacteria\|c__Betaproteobacteria\|o__Burkholderiales\|f__Comamonadaceae\|g__Comamonas\|s__Comamonas_kerstersii\|t__SGB12676 |
| MAG_694 | Other | k__Bacteria\|p__Firmicutes\|c__CFGB1446\|o__OFGB1446\|f__FGB1446\|g__GGB3574\|s__GGB3574_SGB4782\|t__SGB4782 |
| MAG_699 | Other | k__Bacteria\|p__Firmicutes\|c__CFGB2982\|o__OFGB2982\|f__FGB2982\|g__GGB9342\|s__GGB9342_SGB14306\|t__SGB14306 |
| MAG_704 | Other | k__Bacteria\|p__Tenericutes\|c__CFGB1777\|o__OFGB1777\|f__FGB1777\|g__GGB4689\|s__GGB4689_SGB6487\|t__SGB6487 |
| MAG_713 | Family | k__Bacteria\|p__Bacteroidetes\|c__Bacteroidia\|o__Bacteroidales\|f__Prevotellaceae\|g__GGB1145\|s__GGB1145_SGB1471\|t__SGB1471 |
| MAG_714 | Other | k__Bacteria\|p__Firmicutes\|c__CFGB1355\|o__OFGB1355\|f__FGB1355\|g__GGB3306\|s__GGB3306_SGB4373\|t__SGB4373 |
| MAG_717 | Family | k__Bacteria\|p__Firmicutes\|c__Clostridia\|o__Clostridiales\|f__Clostridiaceae\|g__GGB3614\|s__GGB3614_SGB4886\|t__SGB4886 |
| MAG_720 | Family | k__Bacteria\|p__Firmicutes\|c__Clostridia\|o__Clostridiales\|f__Eubacteriaceae\|g__GGB3281\|s__GGB3281_SGB4335\|t__SGB4335 |
| MAG_725 | Species | k__Bacteria\|p__Firmicutes\|c__Clostridia\|o__Clostridiales\|f__Lachnospiraceae\|g__Coprococcus\|s__Coprococcus_comes\|t__SGB4577 |
| MAG_726 | Genus | k__Bacteria\|p__Firmicutes\|c__Clostridia\|o__Clostridiales\|f__Eubacteriaceae\|g__Eubacterium\|s__Eubacterium_SGB4290\|t__SGB4290 |
| MAG_8 | Other | k__Bacteria\|p__Firmicutes\|c__CFGB337\|o__OFGB337\|f__FGB337\|g__GGB765\|s__GGB765_SGB1005\|t__SGB1005 |
| MAG_83 | Family | k__Bacteria\|p__Lentisphaerae\|c__Lentisphaeria\|o__Victivallales\|f__Victivallaceae\|g__GGB6512\|s__GGB6512_SGB9198\|t__SGB9198 |
| MAG_90 | Other | k__Bacteria\|p__Proteobacteria\|c__CFGB2394\|o__OFGB2394\|f__FGB2394\|g__GGB6544\|s__GGB6544_SGB9243\|t__SGB9243 |
| MAG_96 | Family | k__Bacteria\|p__Firmicutes\|c__Clostridia\|o__Clostridiales\|f__Clostridiaceae\|g__GGB3480\|s__GGB3480_SGB4648\|t__SGB4648 |
| MAG_98 | Other | k__Bacteria\|p__Bacteroidetes\|c__CFGB668\|o__OFGB668\|f__FGB668\|g__GGB1663\|s__GGB1663_SGB2279\|t__SGB2279 |

## Table S14. Significantly prevalent species in Chinese or Indian cohorts (adj p-value <= 0.05). The species highlighted in bold have a significantly higher prevalence in the Indian cohort.

| **Species** | **China** | **India** |
| --- | --- | --- |
| ***Bifidobacterium adolescentis*** | **22** | **76** |
| ***Eubacterium rectale*** | **56** | **87** |
| ***Prevotella copri*** | **62** | **88** |
| ***Roseburia faecis*** | **64** | **90** |
| ***Coprococcus eutactus*** | **32** | **63** |
| *Bacteroides cellulosilyticus* | 76 | 6 |
| *Parabacteroides goldsteinii* | 50 | 8 |
| *Bacteroides intestinalis* | 64 | 9 |
| *Butyricimonas virosa* | 76 | 11 |
| *Bacteroides eggerthii* | 62 | 11 |
| *Bacteroides finegoldii* | 54 | 11 |
| *Ruminococcus gnavus* | 76 | 14 |
| *Blautia glucerasea* | 54 | 15 |
| *Bacteroides nordii* | 70 | 18 |
| *Alistipes indistinctus* | 50 | 22 |
| *Flavonifractor plautii* | 78 | 24 |
| *Alistipes onderdonkii* | 54 | 26 |
| *Eubacterium ventriosum* | 62 | 27 |
| *Eubacterium ramulus* | 72 | 28 |
| *Romboutsia timonensis* | 84 | 28 |
| *Bacteroides stercoris* | 82 | 32 |
| *Anaerostipes hadrus* | 96 | 35 |
| *Bilophila wadsworthia* | 84 | 37 |
| *Blautia obeum* | 82 | 40 |
| *Fusicatenibacter saccharivorans* | 78 | 46 |
| *Bacteroides thetaiotaomicron* | 96 | 52 |
| *Ruminococcus torques* | 90 | 53 |
| *Bacteroides caccae* | 92 | 55 |
| *Bacteroides xylanisolvens* | 96 | 55 |
| *Blautia wexlerae* | 96 | 56 |
| *Bacteroides uniformis* | 100 | 65 |
| *Parabacteroides distasonis* | 98 | 70 |
| *Bacteroides ovatus* | 100 | 72 |

## Table S15. Significantly abundant species in Indian cohorts as compared to the Chinese cohort.

| **Species** | **log2FoldChange** |
| --- | --- |
| *Bacteroides eggerthii* | -17.1 |
| *Bacteroides intestinalis* | -8.0 |
| *Ruminococcus gnavus* | -5.1 |
| *Bacteroides cellulosilyticus* | -4.6 |
| *Parabacteroides goldsteinii* | -3.9 |
| *Bacteroides finegoldii* | -3.8 |
| *Butyricimonas virosa* | -3.7 |
| *Bacteroides thetaiotaomicron* | -3.5 |
| *Eubacterium ventriosum* | -2.7 |
| *Bacteroides stercoris* | -2.7 |
| *Bacteroides uniformis* | -2.1 |
| *Anaerostipes hadrus* | -2.0 |
| *Bacteroides xylanisolvens* | -2 |
| *Parabacteroides distasonis* | -1.9 |
| *Escherichia coli* | 1.5 |
| *Dorea longicatena* | 1.6 |
| *Faecalibacterium prausnitzii* | 2.0 |
| *Roseburia faecis* | 2.4 |
| *Ruminococcaceae bacterium* | 2.7 |
| *Eubacterium rectale* | 2.8 |
| *Klebsiella pneumoniae* | 2.8 |
| *Prevotella copri* | 4.3 |
| *Bifidobacterium longum* | 4.6 |
| *Collinsella aerofaciens* | 5.1 |
| *Bifidobacterium adolescentis* | 8.9 |

## Table S16. Differentially **abundant** B-vitamin pathways between Chinese and Indian cohorts (reference China).

| **feature** | **metadata** | **value** | **coefficient** | **stderr** | **N** | **pval** | **qval** |
| --- | --- | --- | --- | --- | --- | --- | --- |
| Riboflavin | Nationality | Indian | -0.67 | 0.08 | 191 | 0 | 0 |
| Thiamine | Nationality | Indian | -0.43 | 0.05 | 191 | 0 | 0 |
| Pyridoxine | Nationality | Indian | -0.78 | 0.16 | 191 | 0 | 0 |
| Biotin | Nationality | Indian | -0.61 | 0.12 | 191 | 0 | 0 |
| Cobalamin | Nationality | Indian | -0.57 | 0.15 | 191 | 0 | 0 |
| Niacin | Gender | Male | 0.21 | 0.07 | 191 | 0 | 0.02 |
| Folate | Nationality | Indian | 0.67 | 0.22 | 191 | 0 | 0.02 |
| Niacin | age | Young_adult | 0.24 | 0.11 | 191 | 0.03 | 0.11 |
| Pyridoxine | Gender | Male | -0.32 | 0.14 | 191 | 0.02 | 0.11 |

## Table S17. Government of India’s Comprehensive National Nutrition Survey (CNNS-2019) data for two vitamins in different age groups.

|  | **Vitamins** | **Cobalamin deficients (in %)** | | | **Folate deficients (in %)** | | |
| --- | --- | --- | --- | --- | --- | --- | --- |
|  | **Age group (years)** | **1-4** | **5-9** | **10-19** | **1-4** | **5-9** | **10-19** |
| **Residence** | **Urban** | 14.4 | 15.6 | 30.3 | 24 | 31.8 | 42.1 |
|  | **Rural** | 13.6 | 17.7 | 31.1 | 23.1 | 27 | 34.9 |
| **Type of diet** | **Veg** | 15.8 | 22.1 | 37.1 | 18.4 | 22.9 | 30.1 |
|  | **Veg+egg** | 13.5 | 14.9 | 33.4 | 30.4 | 35.1 | 47.7 |
|  | **Non-veg** | 11.4 | 10.2 | 21.7 | 28.4 | 34.6 | 43.5 |
